# Supplementary material for: Mechanism of ribosome-associated mRNA degradation during tubulin autoregulation
Source: Mol Cell. 2023 Jul 6;83(13):2290–2302.e13. doi: 10.1016/j.molcel.2023.05.020 (PMC10403363; doi:10.1016/j.molcel.2023.05.020)
Supplement: Document S1. Figures S1–S9, Table S1, and supplemental references [file mmc1.pdf]

Molecular Cell, Volume *83*

## Supplemental information

# Mechanism of ribosome-associated

# mRNA degradation during tubulin autoregulation

**Markus Höpfler, Eva Absmeier, Sew-Yeu Peak-Chew, Evangelia Vartholomaiou, Lori A. Passmore, Ivana Gasic, and Ramanujan S. Hegde**

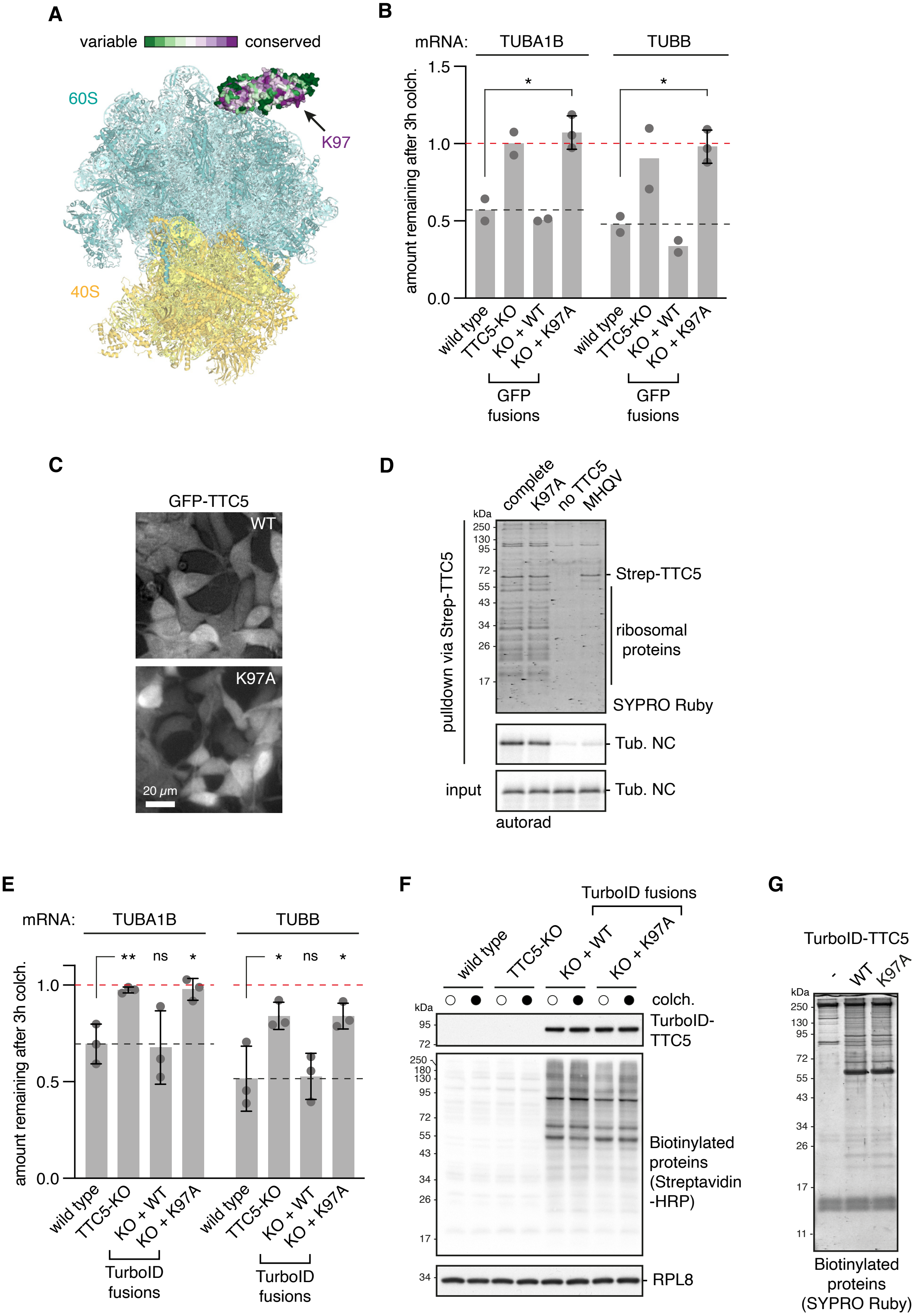

A
variable
conserved
60S
K97
40S
B
mRNA:
TUBA1B
TUBB
amount remaining after 3h colch.
1.5
1.0
0.5
0.0
*
*
wild type
TTC5-KO
KO + WT
KO + K97A
GFP fusions
C
GFP-TTC5
WT
K97A
20 μm
D
complete
K97A
no TTC5
MHQV
kDa
250
130
95
72
55
43
34
26
17
pulldown via Strep-TTC5
Strep-TTC5
ribosomal proteins
SYPRO Ruby
Tub. NC
input
autorad
E
mRNA:
TUBA1B
TUBB
amount remaining after 3h colch.
**
ns
*
TurboID fusions
F
TurboID fusions
wild type
TTC5-KO
KO + WT
KO + K97A
colch.
TurboID-TTC5
Biotinylated proteins (Streptavidin -HRP)
RPL8
G
TurboID-TTC5
-
WT
K97A
11
Biotinylated proteins (SYPRO Ruby)

**Figure S1. The TTC5 K97A mutation abolishes tubulin autoregulation, related to Fig. 1.**

(A) Model of TTC5 bound to the ribosome containing a 64 amino acid β-tubulin nascent chain from a previously published structure [S1] (PDB: 6T59) with the 40S subunit docked for reference. ConSurf [S2] was used to display the residue conservation of TTC5 on the surface. The highly conserved surface patch containing the K97 residue is indicated. This patch is not involved in either ribosome binding or nascent tubulin recognition, leading us to a hypothesis in which it is a docking site for downstream factors. (B) Tubulin autoregulation assay as described in Fig. 1B for TUBB, with TUBA1B reproduced from Fig. 1B for comparison. The red dashed line indicates the starting tubulin mRNA level prior to colchicine, arbitrarily set to a value of 1. The black dashed lines indicate the fold change in wild type (WT) cells for each tubulin subunit. Both TUBA1B and TUBB mRNA were quantified in all experiments in the manuscript. Because the results were comparable for both mRNAs throughout, we display only one tubulin gene in most figures for brevity and clarity. TTC5-KO cells were complemented by stable inducible expression with either GFP-tagged wild type (WT) TTC5 or the K97A mutant. Data show the mean from 2 independent experiments, one of which contained 2 replicates for the TTC5 K97A cell line. Error bars denote standard deviation (SD). Single asterisks denote significant stabilization of tubulin mRNA in KO + K97A cells compared to WT ($p<0.05$) (C) The complemented GFP-tagged cell lines from panel B were imaged using a wide-field microscope (Life Technologies EVOS FL) to verify that expression and localization were similar for both constructs. The same microscope settings were used for both images. (D) Reconstitution of TTC5 recruitment to tubulin ribosome nascent chains (RNCs). 94-residue β-tubulin (TUBB) nascent chains were produced in rabbit reticulocyte lysates in the presence of recombinant WT or K97A mutant Twin-Strep-tagged TTC5 (Strep-TTC5) as indicated. "MHQV" indicates a β-tubulin construct in which its TTC5-interacting MREI motif has been mutated. TTC5-associated proteins were enriched via its Strep tag, separated by SDS-PAGE and visualized by SYPRO Ruby staining for total protein (top) or autoradiography for the β-tubulin nascent chain (Tub. NC, bottom). (E) Autoregulation assay with the indicated WT, TTC5-KO, or TurboID-TTC5 rescue cell lines. Data show the mean ± SD from 2 independent experiments, one of which contained 2 replicates. Single and double asterisks indicate $p<0.05$ and $p<0.01$, respectively; "ns" indicates not significant. (F) Western blot analysis of cell lines used in panel E. Cells were pre-treated with 10 $\mu$M colchicine (colch.) for 30 minutes as indicated by filled circles, and 50 $\mu$M biotin was added for 2.5 h (all samples). (G) Biotinylated proteins enriched from parental, TurboID-TTC5 WT or K97A cell lines as detected by SYPRO Ruby staining. Samples prepared in the same way were used for the mass spectrometry experiment in Fig. 1C. 50 $\mu$M biotin was added 2.5h prior to harvesting of cells. To avoid over-expression of TurboID-TTC5 constructs in biotinylation experiments, we exploited the basal leaky expression from the tetracycline-regulated CMV promoter seen in the absence of added doxycycline.

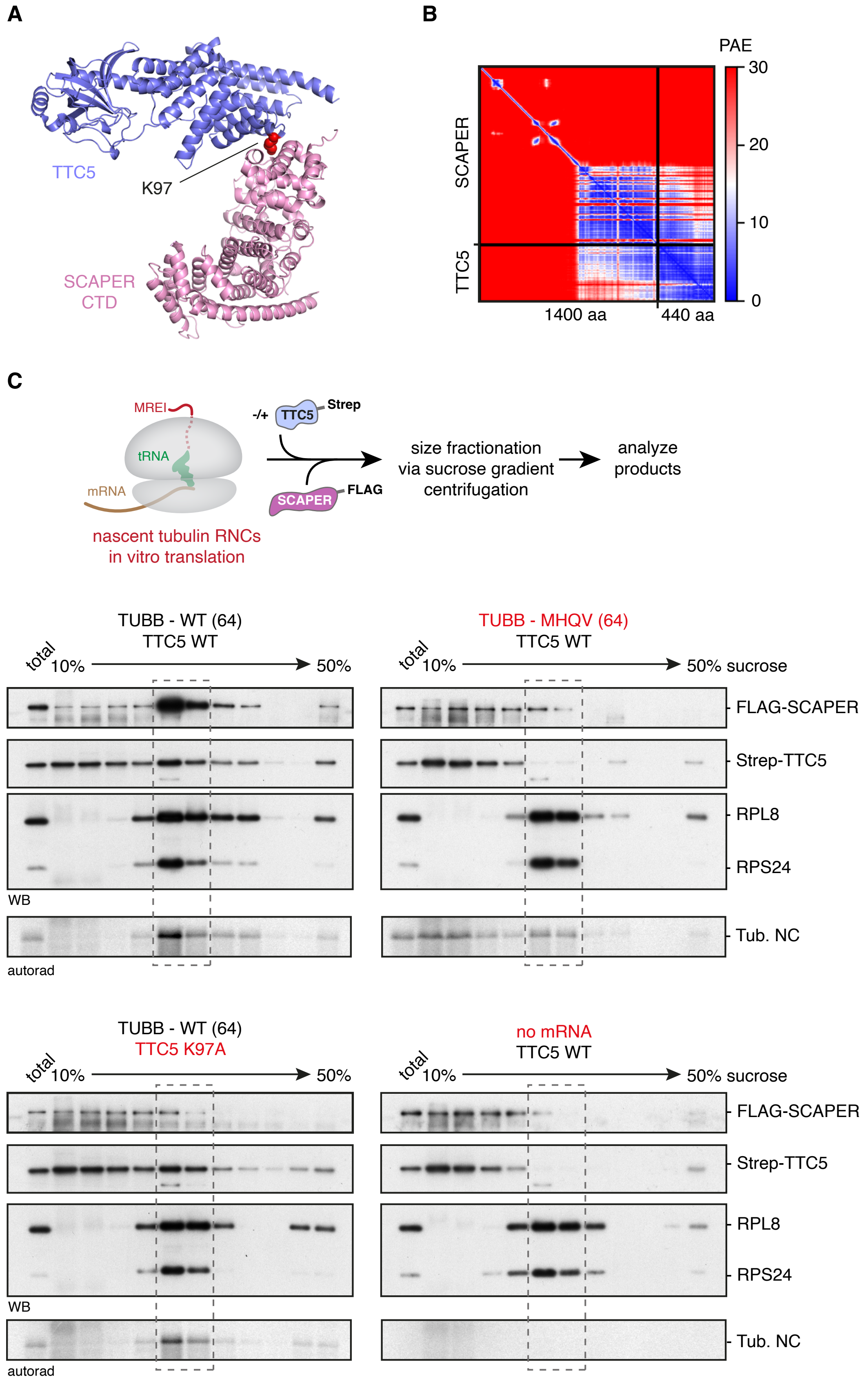
A
TTC5
K97
SCAPER
CTD
B
PAE
30
20
10
0
SCAPER
TTC5
1400 aa
440 aa
C
MREI
tRNA
mRNA
nascent tubulin RNCs
in vitro translation
-/+
TTC5
Strep
SCAPER
FLAG
size fractionation
via sucrose gradient
centrifugation
analyze
products
TUBB - WT (64)
TTC5 WT
total
10%
50%
TUBB - MHQV (64)
TTC5 WT
50% sucrose
FLAG-SCAPER
Strep-TTC5
RPL8
RPS24
WB
Tub. NC
autorad
TUBB - WT (64)
TTC5 K97A
no mRNA
TTC5 WT

**Figure S2. TTC5 recruits SCAPER to tubulin ribosome nascent chain complexes, related to Fig. 2.** (A) Top-ranked AlphaFold2 (AF2) multimer prediction of the interaction between TTC5 and SCAPER. The SCAPER C-terminal domain (CTD, residues 857–1400) is shown, with the rest of the molecule omitted for clarity. The TTC5 K97 residue is highlighted in red spheres. (B) Predicted Aligned Error (PAE) matrix of the TTC5-SCAPER AF2 multimer prediction shown in panel A. Lower PAE values (blue) indicate higher confidence in the relative positions and orientation between residue pairs in the model. Note that a strong signal is observed between TTC5 and the CTD of SCAPER. (C) 64-residue β-tubulin (TUBB) nascent chain complexes were produced by in vitro translation in the presence of SCAPER and TTC5 as in Fig. 2C. Reactions were then centrifuged through 10–50% sucrose gradients and fractions were analysed by SDS-PAGE, western blotting (WB), and autoradiography (autorad). An aliquot of the total reaction is analyzed in the first lane of each gradient. The peak ribosome-containing fractions, predominantly monosomes, are indicated by dashed boxes. Both the WT β-tubulin sequence and WT TTC5 are required for efficient recruitment of SCAPER to RNCs.

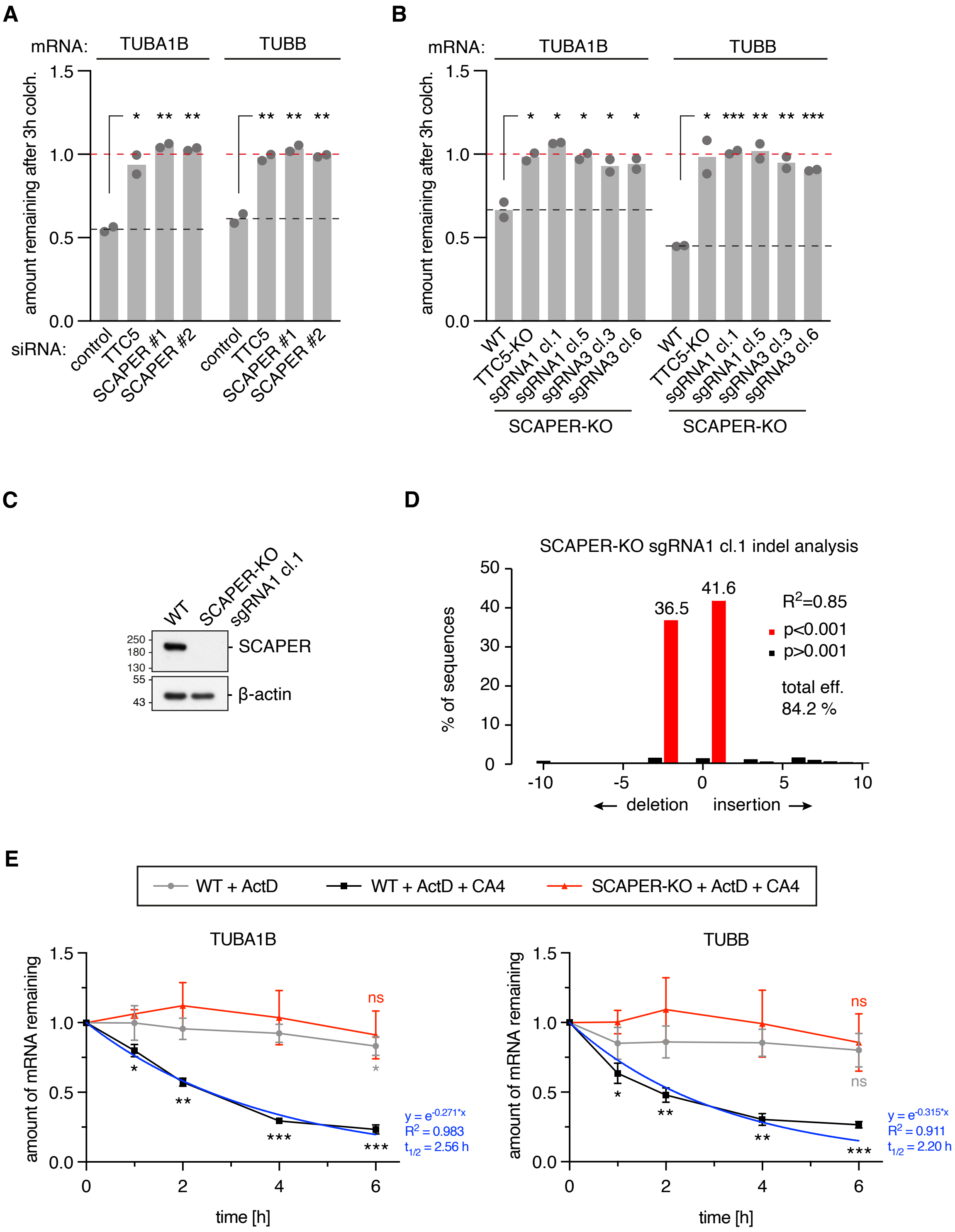

A
mRNA:
TUBA1B
TUBB
amount remaining after 3h colch.
siRNA:
control
TTC5
SCAPER #1
SCAPER #2
B
WT
TTC5-KO
sgRNA1 cl.1
sgRNA1 cl.5
sgRNA3 cl.3
sgRNA3 cl.6
SCAPER-KO
C
SCAPER-KO sgRNA1 cl.1
SCAPER
β-actin
D
SCAPER-KO sgRNA1 cl.1 indel analysis
% of sequences
36.5
41.6
R2=0.85
p<0.001
p>0.001
total eff. 84.2 %
deletion
insertion
E
WT + ActD
WT + ActD + CA4
SCAPER-KO + ActD + CA4
TUBA1B
TUBB
amount of mRNA remaining
time [h]
ns
y = e-0.271*x
R2 = 0.983
t1/2 = 2.56 h
y = e-0.315*x
R2 = 0.911
t1/2 = 2.20 h

**Figure S3. SCAPER is required for tubulin autoregulation, related to Fig. 2.**
(A) Tubulin mRNA levels were determined in the qPCR-based autoregulation assay for cells transfected with the indicated siRNAs for 72h. Samples from colchicine-treated cells (10 $\mu$M for 3h) were normalized to untreated control samples, arbitrarily set to a value of 1 (red dashed line). The black dashed lines indicate the fold change in wild type (WT) cells for each set of samples. Data show the mean from 2 independent experiments. (B) Autoregulation assays were performed using the indicated HEK293 T-REx cell lines or four different SCAPER KO clones (cl.) obtained from two different sgRNAs. Data show the mean from 2 independent experiments. Single, double, and triple asterisks indicate $p<0.05$, $p<0.01$, and $p<0.001$, respectively. (C) Whole cell lysates from HEK T-REx WT and SCAPER KO (sgRNA1 cl.1) were analyzed by SDS-PAGE and western blotting using anti-SCAPER and anti-$\beta$-actin antibodies. (D) Indel analysis for SCAPER KO sgRNA1 cl.1 using genomic DNA sequencing followed by TIDE analysis [S3]. This clone shows a 1-nucleotide insertion and a 2-nucleotide deletion for the two alleles. sgRNA1 targets exon 5 around codon 127. The same clone was used throughout the rest of the study and to generate rescue cell lines. (E) Quantification of mRNA decay rates in HEK T-REx WT and SCAPER KO cells after transcription shut-down with actinomycin D (ActD) for $\alpha$-tubulin (TUBA1B, top) and $\beta$-tubulin (TUBB, bottom). Cells were treated with 5 $\mu$g/ml ActD or 100 nM combretastatin A4 (CA4) from $t = 0$ as indicated. TUBA1B and TUBB mRNAs were quantified by RT-qPCR and normalized to GAPDH (a transcript that was stable for the observed period) and the $t = 0$ value. Statistical analysis was performed using a one-sample $t$-test. Values significantly different from 1 ($t = 0$ value) are indicated by single, double, and triple asterisks ($p<0.05$, $p<0.01$, and $p<0.001$, respectively; “ns” indicates not significant). For the WT + ActD + CA4 samples, an exponential decay curve was fitted to the data (blue curve and parameters). Data show mean ± SD from 3 replicates.

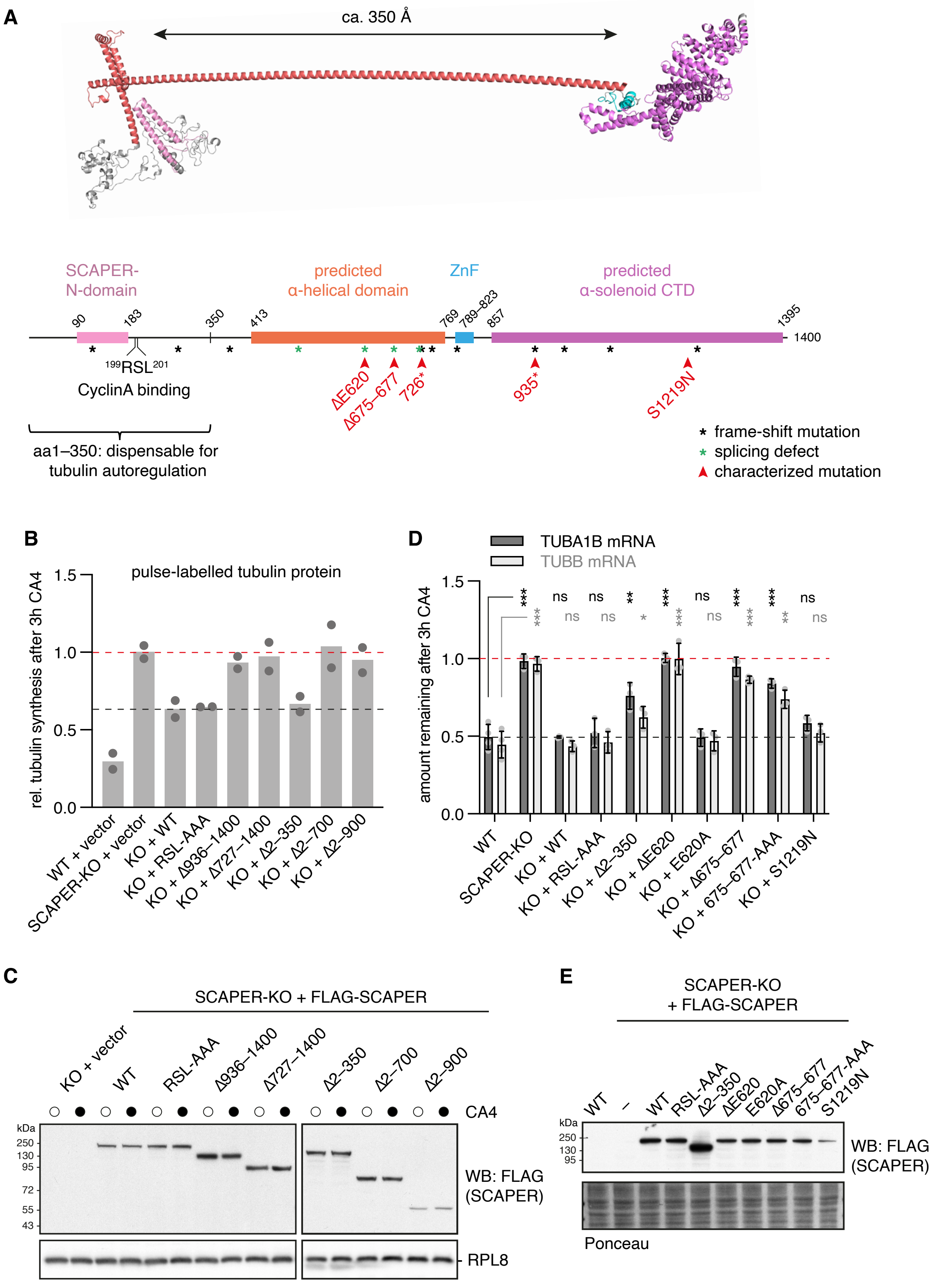
A
ca. 350 Å
SCAPER-
N-domain
predicted
α-helical domain
ZnF
predicted
α-solenoid CTD
90
183
350
413
769
789–823
857
1395
1400
199RSL201
CyclinA binding
aa1–350: dispensable for
tubulin autoregulation
ΔE620
Δ675–677
726*
935*
S1219N
frame-shift mutation
splicing defect
characterized mutation
B
pulse-labelled tubulin protein
rel. tubulin synthesis after 3h CA4
1.5
1.0
0.5
0.0
WT + vector
SCAPER-KO + vector
KO + WT
KO + RSL-AAA
KO + Δ936–1400
KO + Δ727–1400
KO + Δ2–350
KO + Δ2–700
KO + Δ2–900
D
TUBA1B mRNA
TUBB mRNA
amount remaining after 3h CA4
1.5
1.0
0.5
0.0
***
ns
ns
*
***
ns
***
***
ns
***
ns
ns
*
***
ns
***
**
ns
WT
SCAPER-KO
KO + WT
KO + RSL-AAA
KO + Δ2–350
KO + ΔE620
KO + E620A
KO + Δ675–677
KO + 675–677-AAA
KO + S1219N
C
SCAPER-KO + FLAG-SCAPER
KO + vector
WT
RSL-AAA
Δ936–1400
Δ727–1400
Δ2–350
Δ2–700
Δ2–900
CA4
kDa
250
130
95
72
55
43
WB: FLAG
(SCAPER)
RPL8
E
SCAPER-KO
+ FLAG-SCAPER
WT
–
WT
RSL-AAA
Δ2–350
ΔE620
E620A
Δ675–677
675–677-AAA
S1219N
kDa
250
130
95
WB: FLAG
(SCAPER)
Ponceau

**Figure S4. The SCAPER α-helical and C-terminal domains are critical for tubulin autoregulation, related to Fig. 2.**

(A) Top: Structural model of SCAPER predicted by trRosetta [S4]. The displayed model was stitched from two overlapping predictions. Subdomains are color-coded as in the schematic below. Bottom: Schematic of the SCAPER domain architecture with annotated and predicted features indicated (drawn to scale). Pathologic mutations [S5–S7] characterized in autoregulation assays in this study are indicated by red arrow heads. Positions of pathologic frameshift and splice site mutations are indicated by black and green asterisks, respectively. RSL: cyclin A binding motif ($Arg^{199}$-$Ser^{200}$-$Leu^{201}$); ZnF: Zinc finger; CTD: Carboxy-terminal domain. (B) Tubulin autoregulation was tested by pulse-labelling of newly synthesized proteins with $^{35}S$-methionine. Indicated cell lines were transiently transfected with FLAG-SCAPER encoding plasmids. Plotted is the ratio of $^{35}S$-labelled tubulins from cells after 3h combretastatin A4 (CA4) treatment versus untreated control conditions as quantified by autoradiography. RSL-AAA: mutation of the cyclin A binding site ($Arg^{199}$-$Ser^{200}$-$Leu^{201}$) to alanines. Numbers refer to SCAPER amino acid (aa) positions. Mean and individual data points from two independent experiments are plotted. We note that pathologic alleles with truncations after residue 726 and 935 have been reported to cause disease phenotypes such as intellectual disability, a Bardet-Biedl syndrome-like illness, and male infertility [S6, S8]. (C) Total protein analysis by western blotting of cells used in panel B. (D) Autoregulation assay with WT, SCAPER-KO and the indicated stable rescue cell lines. Note that part of the data is reproduced from Fig. 2D for comparison. Data show the mean ± SD from 2 independent experiments, one of which contained 2 replicates. For statistical comparisons against the WT cell line for each tubulin isoform, single, double, and triple asterisks indicate $p<0.05$, $p<0.01$, and $p<0.001$, respectively; “ns” indicates not significant. (E) Western blot analysis of SCAPER expression levels of cell lines used in panel D and Fig. 2D. Note that the SCAPER-S1219N variant shows lower expression levels. This indicates that the mutation might destabilize the protein, a potential explanation for the pathologic phenotype it has in a heterozygous patient with the ΔE620 mutation on the second allele [S5]. Under overexpression conditions, SCAPER-S1219N is functional for autoregulation (see panel D).

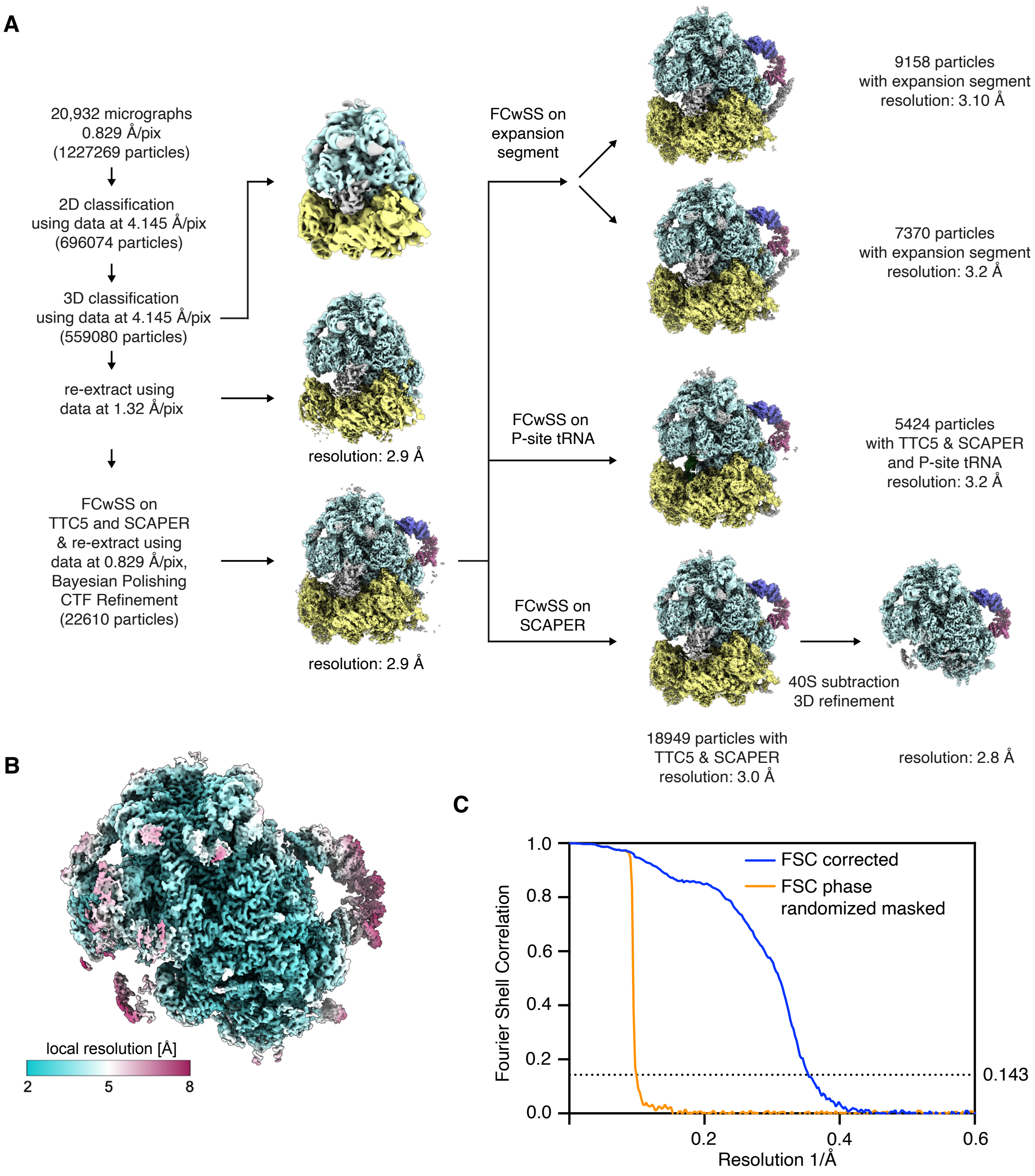


**Figure S5. Cryo-EM analysis of SCAPER bound to TTC5 and 80S ribosomes, related to Fig. 3.**
(A) RELION 4.0 classification and processing workflow for reconstruction of ribosomal particles from cryo-EM micrographs. FCwSS: Focussed classification with signal subtraction. (B) Map coloured according to the local resolution after final FCwSS around SCAPER and subtraction of the 40S subunit. (C) Gold-standard Fourier shell correlation (FSC) curve (blue) of the final 60S-TTC5-SCAPER map illustrating an overall resolution of 2.8 Å. The phase-randomized, masked FSC curve (orange) is also shown.

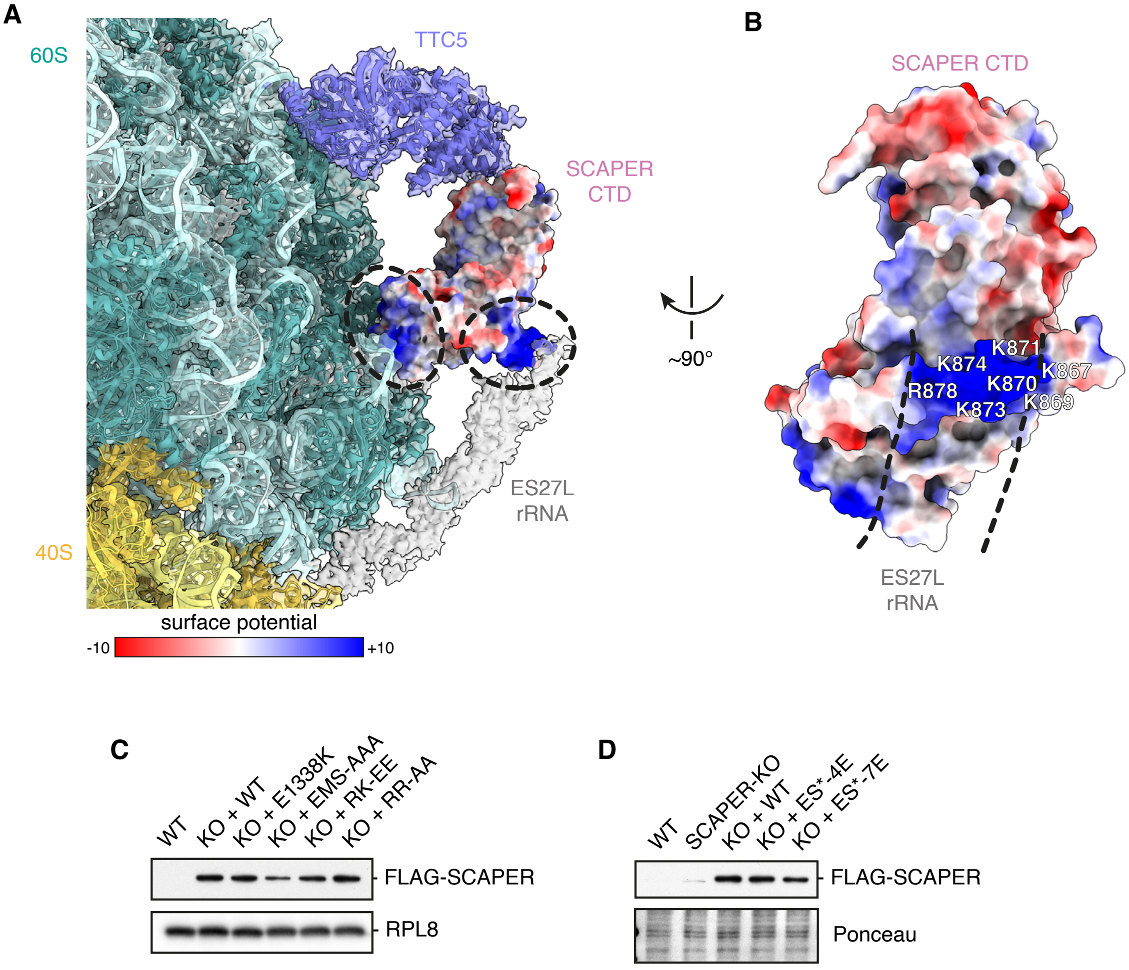


**Figure S6. SCAPER contacts with 28S rRNA expansion segment ES27L, related to Fig. 3.**
(A) View of the cryo-EM map with electrostatic surface potential [in kcal/(mol**e*)] displayed on the SCAPER CTD model. Note the two highly basic surface patches on SCAPER (dashed outlines) that contact the 60S body and ES27L rRNA. (B) Side-view of the SCAPER-CTD model from panel A with the path of the density for the rRNA expansion segment ES27L marked by dashed lines. Positively charged residues that were targeted by mutagenesis are indicated. (C, D) Western blot analysis of expression levels of FLAG-SCAPER constructs used in Fig. 3E and 3F, respectively. EMS-AAA: E1338A, M1339A, S1340A; RK-EE: R907E, K910E; RR-AA: R934A, R941A. SCAPER mutations targeting expansion segment contacts were as follows: ES*-4E: K867E, K870E, K873E and K874E; ES*-7E: as ES*-4E plus K869E, K871E and R878E.

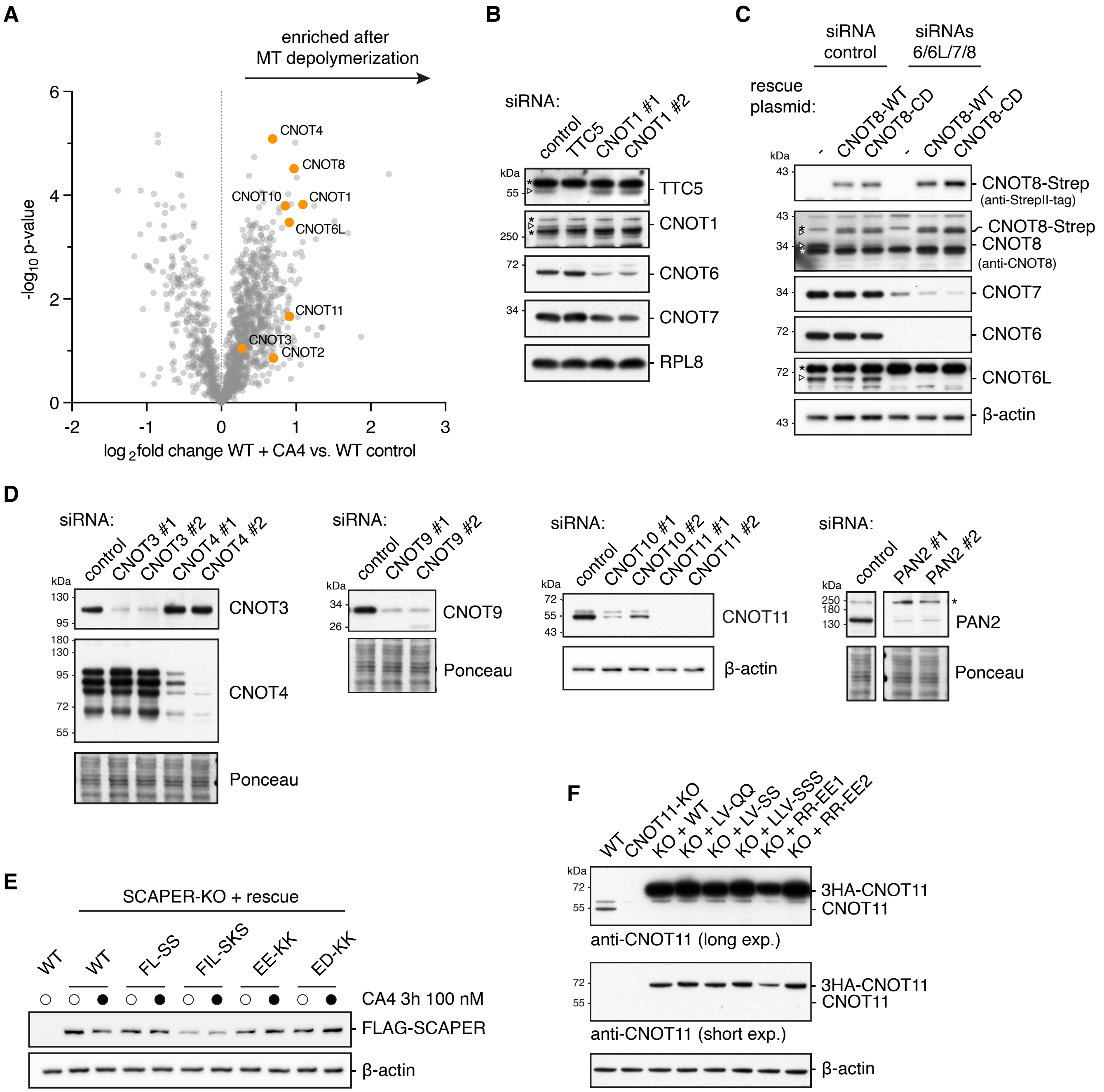
A
enriched after
MT depolymerization
CNOT4
CNOT8
CNOT10
CNOT1
CNOT6L
CNOT11
CNOT3
CNOT2
-log10 p-value
log 2 fold change WT + CA4 vs. WT control
B
siRNA:
control
TTC5
CNOT1 #1
CNOT1 #2
kDa
TTC5
CNOT1
CNOT6
CNOT7
RPL8
C
siRNA control
siRNAs 6/6L/7/8
rescue plasmid:
CNOT8-WT
CNOT8-CD
CNOT8-Strep
(anti-StrepII-tag)
CNOT8-Strep
CNOT8
(anti-CNOT8)
CNOT7
CNOT6
CNOT6L
β-actin
D
siRNA:
control
CNOT3 #1
CNOT3 #2
CNOT4 #1
CNOT4 #2
CNOT3
CNOT4
Ponceau
control
CNOT9 #1
CNOT9 #2
CNOT9
Ponceau
control
CNOT10 #1
CNOT10 #2
CNOT11 #1
CNOT11 #2
CNOT11
β-actin
control
PAN2 #1
PAN2 #2
PAN2
Ponceau
E
SCAPER-KO + rescue
WT
WT
FL-SS
FIL-SKS
EE-KK
ED-KK
CA4 3h 100 nM
FLAG-SCAPER
β-actin
F
WT
CNOT11-KO
KO + WT
KO + LV-QQ
KO + LV-SS
KO + LLV-SSS
KO + RR-EE1
KO + RR-EE2
3HA-CNOT11
CNOT11
anti-CNOT11 (long exp.)
3HA-CNOT11
CNOT11
anti-CNOT11 (short exp.)
β-actin

**Figure S7. The CCR4-NOT complex is required for tubulin autoregulation, related to Fig. 4 and 5.**
(A) Proximity labelling using HEK T-REx TurboID-SCAPER WT cells was performed in control conditions or after microtubule (MT) depolymerization with 100 nM Combretastatin A4 (CA4). Biotinylated proteins were enriched and analyzed by quantitative mass spectrometry. See also Supplementary Table S3. 3 replicates were analyzed for both conditions. (B) Western blot analysis of samples as used in Fig. 4D confirming knockdown of TTC5 and CNOT1. Note that depletion of the CNOT1 scaffolding subunit also leads to destabilization of other CCR4-NOT subunits (CNOT6, CNOT7). Specific bands are indicated by triangles for TTC5 and CNOT1; non-specific bands are indicated by asterisks. (C) Western blot analysis of samples used in Fig. 4E. Signals from blots with an antibody against endogenous CNOT8 (second panel) show both CNOT8 depletion after knockdown and expression of siRNA-resistant rescue constructs CNOT8-WT and CNOT8-CD. Specific bands are indicated by triangles for CNOT8 and CNOT6L; non-specific bands are indicated by asterisks. (D) Western blot analysis of knockdown efficiency for selected samples used in Fig. 5A. Although we did not have access to a functional CNOT10 antibody, we note that CNOT10-KD leads to destabilization of CNOT11, corroborating that they might act as a functional unit. (E) Western blot analysis of expression levels of FLAG-SCAPER constructs used in Fig. 5D. FL-SS: F628S, L632S; FIL-SKS: F628S, I629K, L632S; EE-KK: E618K, E625K; ED-KK: E633K, D640K. (F) Western blot analysis of expression levels of CNOT11 and rescue constructs used in Fig. 5E. Membranes were probed with an antibody raised against a peptide of endogenous CNOT11. The epitope was not affected by the indicated mutations. LV-QQ: L405Q, V454Q; LV-SS: L405S, V454S; LLV-SSS: L405S, L451S, V454S; RR-EE1: R447E, R450E; RR-EE2: R461E, R485E.

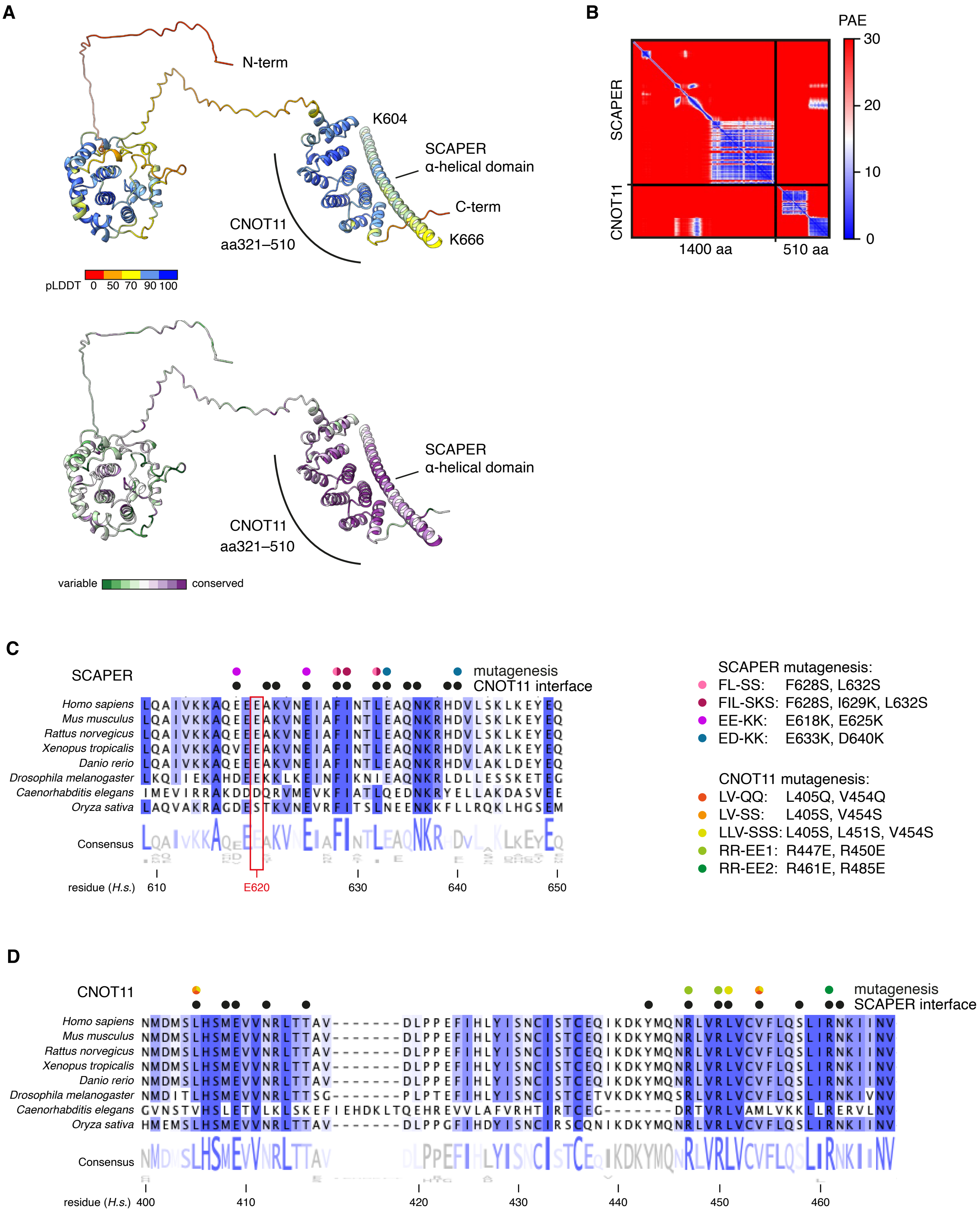
A
N-term
K604
SCAPER
α-helical domain
C-term
K666
CNOT11
aa321–510
pLDDT 0 50 70 90 100
SCAPER
α-helical domain
CNOT11
aa321–510
variable
conserved
B
PAE
30
20
10
0
SCAPER
CNOT11
1400 aa
510 aa
C
SCAPER
mutagenesis
CNOT11 interface
Homo sapiens LQAIVKKAQEEEAKVNEIAFINTLEAQNKRHDVLSKLKEYEQ
Mus musculus LQAIVKKAQEEEAKVNEIAFINTLEAQNKRHDVLSKLKEYEQ
Rattus norvegicus LQAIVKKAQEEEAKVNEIAFINTLEAQNKRHDVLSKLKEYEQ
Xenopus tropicalis LQAIVKKAQVEEAKVNEIAFINTLEAQNKRHDVLAKLKEYEQ
Danio rerio LQAIVKKAQEEEAKVNEIAFINTLEAQNKRHDVLAKLDEYEQ
Drosophila melanogaster LKQIIEKAHDEEKKLKEINFIKNIEAQNKRLDLLESSKETEG
Caenorhabditis elegans IMEVIRRAKDDDQRVMEVKFIATLQEDNKRYELLAKDASVEE
Oryza sativa LAQVAKRAGDESTKVNEVRFITSLNEENKKFLLRQKLHGSEM
Consensus
residue (H.s.) 610 E620 630 640 650
SCAPER mutagenesis:
FL-SS: F628S, L632S
FIL-SKS: F628S, I629K, L632S
EE-KK: E618K, E625K
ED-KK: E633K, D640K
CNOT11 mutagenesis:
LV-QQ: L405Q, V454Q
LV-SS: L405S, V454S
LLV-SSS: L405S, L451S, V454S
RR-EE1: R447E, R450E
RR-EE2: R461E, R485E
D
CNOT11
mutagenesis
SCAPER interface
Homo sapiens NMDMSLHSMEVVNRLTTAV-------DLPPEFIHLYISNCISTCEQIKDKYMQNRLVRLVCVFLQSLIRNKIINV
Mus musculus NMDMSLHSMEVVNRLTTAV-------DLPPEFIHLYISNCISTCEQIKDKYMQNRLVRLVCVFLQSLIRNKIINV
Rattus norvegicus NMDMSLHSMEVVNRLTTAV-------DLPPEFIHLYISNCISTCEQIKDKYMQNRLVRLVCVFLQSLIRNKIINV
Xenopus tropicalis NMDMSLHSMEVVNRLTTAV-------DLPPEFIHLYISNCISTCEQIKDKYMQNRLVRLVCVFLQSLIRNKIINV
Danio rerio NMDMSLHSMEVVNRLTTAV-------DLPPEFIHLYISNCISTCEQIKDKYMQNRLVRLVCVFLQSLIRNKIINV
Drosophila melanogaster NMDITLHSMEVVNRLTTSG-------PLPTEFIHLYISNCISTCETVKDKYMQSRLVRLVCVFLQSLIRNKIVNV
Caenorhabditis elegans GVNSTVHSLETVLKLSKEFIEHDKLTQEHREVVLAFVRHTIRTCEG-------DRTVRLVAMLVKKLLRERVLNV
Oryza sativa HMEMSLHSMEVVNRLTTAV-------DLPPGFIHDYISNCIRSCQNIKDKYMQNRLVRLVCVFLQSLIRNKIINV
Consensus
residue (H.s.) 400 410 420 430 440 450 460

**Figure S8. Structural prediction of a putative SCAPER-CNOT11 complex, related to Fig. 5.**
(A) Overview of an AF2 multimer predicted complex between CNOT11 and SCAPER coloured by predicted local distance difference test score (pLDDT, top, dark blue indicates high confidence), or per residue conservation (bottom). For SCAPER, only residues 604–666 of the $\alpha$-helical domain are displayed for clarity. (B) PAE matrix of the model displayed in panel A. (C) Multiple sequence alignment of SCAPER homologs across the indicated species generated using Clustal Omega and displayed using Jalview software [S9, S10]. Highly conserved residues are shaded in blue. Residues in close proximity to CNOT11 in the AF2 predicted model are marked with black dots, and residues targeted in mutagenesis experiments are marked with color-coded dots. The E620 residue is highlighted by a red box. (D) Multiple sequence alignment of CNOT11 homologs generated as in panel C. Residues in close proximity to SCAPER in the AF2 model are marked by black dots, and residues targeted by mutagenesis are shown with coloured dots. Residue R485 is not shown in this alignment.

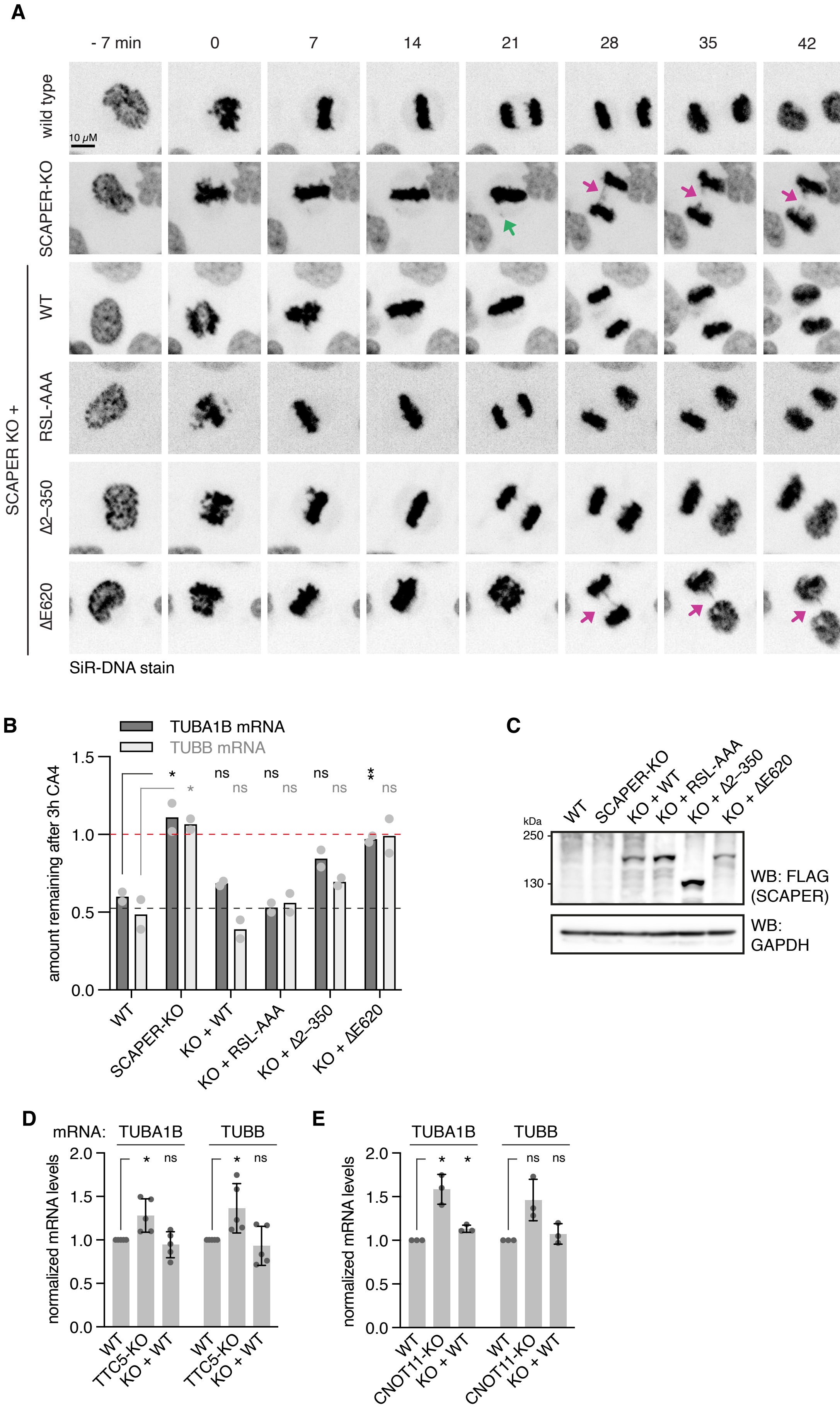
A
- 7 min
0
7
14
21
28
35
42
wild type
10 μM
SCAPER-KO
SCAPER KO +
WT
RSL-AAA
Δ2–350
ΔE620
SiR-DNA stain
B
TUBA1B mRNA
TUBB mRNA
amount remaining after 3h CA4
1.5
1.0
0.5
0.0
*
ns
ns
ns
**
*
ns
ns
ns
ns
WT
SCAPER-KO
KO + WT
KO + RSL-AAA
KO + Δ2–350
KO + ΔE620
C
WT
SCAPER-KO
KO + WT
KO + RSL-AAA
KO + Δ2–350
KO + ΔE620
kDa
250
130
WB: FLAG (SCAPER)
WB: GAPDH
D
mRNA:
TUBA1B
TUBB
normalized mRNA levels
2.0
1.5
1.0
0.5
0.0
*
ns
*
ns
WT
TTC5-KO
KO + WT
WT
TTC5-KO
KO + WT
E
TUBA1B
TUBB
normalized mRNA levels
2.0
1.5
1.0
0.5
0.0
*
*
ns
ns
WT
CNOT11-KO
KO + WT
WT
CNOT11-KO
KO + WT

**Figure S9. SCAPER is required for faithful mitosis, related to Fig. 6.**
(A) Examples from time-lapse imaging of HeLa T-REx cells of the indicated genotype going through mitosis. DNA was visualized using SiR-DNA stain and maximum intensity projections are shown. Frames were aligned to nuclear envelope breakdown ($t = 0$). Misaligned chromosomes and segregation errors are highlighted by green and magenta arrows, respectively. (B) Autoregulation assay for HeLa cell lines used in panel A and Fig. 6B and 6C. Data show the mean from 2 independent experiments. For statistical comparisons against the WT cell line for each tubulin isoform, single and double asterisks indicate $p<0.05$ and $p<0.01$, respectively; "ns" indicates not significant. See also Fig. 2D for data from HEK293 T-REx cells. (C) Total protein analysis by western blotting for FLAG-tagged SCAPER in the HeLa cell lines used in panels A, B, and Fig. 6B and 6C. (D–E) Quantification of steady state tubulin mRNA levels in the indicated HEK T-REx cell lines. Tubulin mRNA levels were normalized to a reference gene (RPLP1) and to the WT cell line, and data from all relevant experiments in the manuscript were compiled. Data show mean ± SD. Statistical analysis for KO and rescue cell lines was performed using a one-sample *t*-test. Values significantly different from 1 (WT levels) are indicated by single asterisks ($p<0.05$; "ns" indicates not significant). (D) $n = 5$, data reanalyzed from Fig. S1B and S1E. (E) $n = 3$, data reanalyzed from Fig. 5E.

**Table S1. Cryo-EM data collection, processing, refinement and model statistics. Related to Fig. 3.**

| Data Collection | Dataset 1 |
|---|---|
| Voltage (keV) | 300 |
| Pixel size (Å) | 0.829 |
| Detector | Gatan K3 |
| Defocus range (µm) | -1.2 to -2.7 |
| Electron dose ($e^{-}$ $frame^{-1}$ $Å^{-2}$) | 1 |
| **Data Processing** | |
| Useable micrographs | 19176 of 20932 |
| Particles picked | 1227269 |
| Final particles | 18949 |
| Map sharpening B-factor ($Å^2$) | -20 |
| Map resolution (Å) | 2.8 (0.143 FSC) |
| EMDB accession code | EMD-16155 |
| PDB accession code | 8BPO |
| **Model Composition** | |
| Chains | 62 |
| Non-hydrogen atoms | 143326 |
| Protein residues | 7633 |
| RNA bases | 3814 |
| Metals ($Mg^{2+}$/$Zn^{2+}$) | 214/5 |
| **Refinement** | |
| Model resolution (Å) | 2.9 (0.5 FSC)<br>2.4 (0.143 FSC) |
| CC (mask) | 0.85 |
| **R.M.S deviations** | |
| Bond lengths (Å) | 0.004 |
| Bond angles (°) | 0.851 |
| **Validation** | |
| Molprobity score | 1.40 |
| Clashscore, all atoms | 3.79 |
| Rotamers outliers (%) | 0.03 |
| Cβ outliers (%) | 0.03 |
| **Ramachandran plot** | |
| Favored (%) | 96.47 |
| Allowed (%) | 3.50 |
| Outliers (%) | 0.03 |

## SUPPLEMENTAL REFERENCES

S1. Lin, Z., Gasic, I., Chandrasekaran, V., Peters, N., Shao, S., Mitchison, T.J., and Hegde, R.S. (2020). TTC5 mediates autoregulation of tubulin via mRNA degradation. Science *367*, 100–104. 10.1126/science.aaz4352.

S2. Ashkenazy, H., Abadi, S., Martz, E., Chay, O., Mayrose, I., Pupko, T., and Ben-Tal, N. (2016). ConSurf 2016: an improved methodology to estimate and visualize evolutionary conservation in macromolecules. Nucleic Acids Res *44*, W344–W350. 10.1093/nar/gkw408.

S3. Brinkman, E.K., Chen, T., Amendola, M., and van Steensel, B. (2014). Easy quantitative assessment of genome editing by sequence trace decomposition. Nucleic Acids Res *42*, e168–e168. 10.1093/nar/gku936.

S4. Yang, J., Anishchenko, I., Park, H., Peng, Z., Ovchinnikov, S., and Baker, D. (2020). Improved protein structure prediction using predicted interresidue orientations. Proc National Acad Sci *117*, 1496–1503. 10.1073/pnas.1914677117.

S5. Tatour, Y., Sanchez-Navarro, I., Chervinsky, E., Hakonarson, H., Gawi, H., Tahsin-Swafiri, S., Leibu, R., Lopez-Molina, M.I., Fernandez-Sanz, G., Ayuso, C., et al. (2017). Mutations in SCAPER cause autosomal recessive retinitis pigmentosa with intellectual disability. J Med Genet *54*, 698. 10.1136/jmedgenet-2017-104632.

S6. Fasham, J., Arno, G., Lin, S., Xu, M., Carss, K.J., Hull, S., Lane, A., Robson, A.G., Wenger, O., Self, J.E., et al. (2019). Delineating the expanding phenotype associated with SCAPER gene mutation. Am J Med Genet A *179*, 1665–1671. 10.1002/ajmg.a.61202.

S7. Wormser, O., Gradstein, L., Yogev, Y., Perez, Y., Kadir, R., Goliand, I., Sadka, Y., Riati, S.E., Flusser, H., Nachmias, D., et al. (2019). SCAPER localizes to primary cilia and its mutation affects cilia length, causing Bardet-Biedl syndrome. Eur J Hum Genet *27*, 928–940. 10.1038/s41431-019-0347-z.

S8. Wormser, O., Levy, Y., Bakhrat, A., Bonaccorsi, S., Graziadio, L., Gatti, M., AbuMadighem, A., McKenney, R.J., Okada, K., Riati, S.E., et al. (2021). Absence of SCAPER causes male infertility in humans and Drosophila by modulating microtubule dynamics during meiosis. J Med Genet *58*, 254–263. 10.1136/jmedgenet-2020-106946.

S9. Sievers, F., Wilm, A., Dineen, D., Gibson, T.J., Karplus, K., Li, W., Lopez, R., McWilliam, H., Remmert, M., Söding, J., et al. (2011). Fast, scalable generation of high-quality protein multiple sequence alignments using Clustal Omega. Mol Syst Biol *7*, 539–539. 10.1038/msb.2011.75.

S10. Waterhouse, A.M., Procter, J.B., Martin, D.M.A., Clamp, M., and Barton, G.J. (2009). Jalview Version 2—a multiple sequence alignment editor and analysis workbench. Bioinformatics *25*, 1189–1191. 10.1093/bioinformatics/btp033.
