# Supplementary material for: Mechanism of ribosome-associated mRNA degradation during tubulin autoregulation
Source: Mol Cell. 2023 Jul 6;83(13):2290–2302.e13. doi: 10.1016/j.molcel.2023.05.020 (PMC10403363; doi:10.1016/j.molcel.2023.05.020)
Supplement: Document S2. Article plus supplemental information [file mmc6.pdf]

Molecular Cell

Article

Mechanism of ribosome-associated mRNA degradation during tubulin autoregulation

Graphical abstract

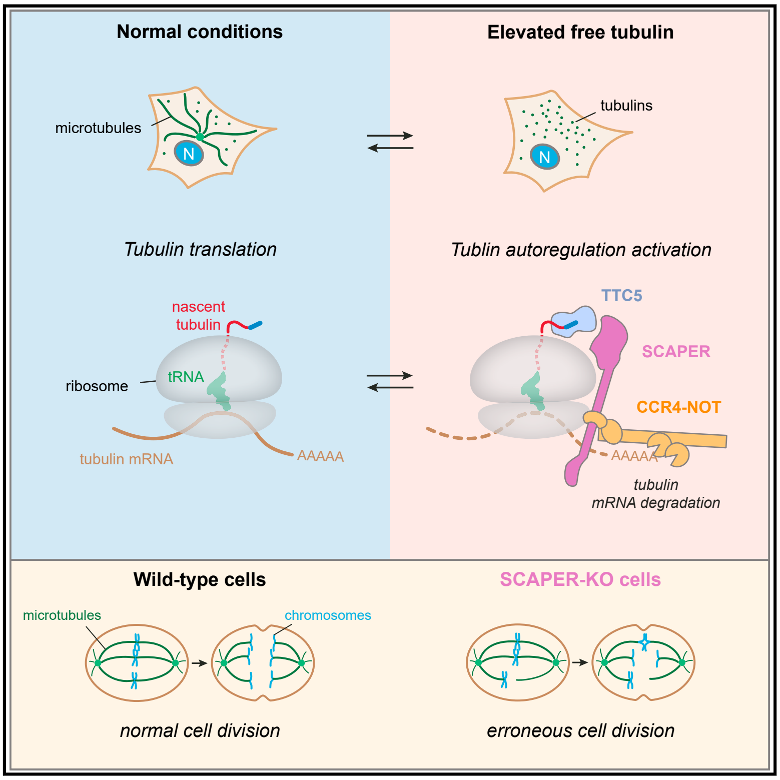

Authors

Markus Höpfler, Eva Absmeier, Sew-Yeu Peak-Chew, Evangelia Vartholomaiou, Lori A. Passmore, Ivana Gasic, Ramanujan S. Hegde

Correspondence

rhegde@mrc-lmb.cam.ac.uk

In brief

Cells with elevated unpolymerized tubulin selectively degrade tubulin-encoding mRNAs. Höpfler et al. reveal that TTC5, which recognizes nascent tubulin on translating ribosomes, recruits SCAPER and the CCR4-NOT deadenylase to degrade tubulin mRNAs. Loss of tubulin autoregulation causes aberrant mitosis in cells and is associated with complex neurodevelopmental diseases in humans.

Highlights

- Tubulin-synthesizing ribosomes engaged by TTC5 recruit the adaptor SCAPER
- Ribosome-associated SCAPER recruits CCR4-NOT, which degrades tubulin mRNAs
- The TTC5-SCAPER-CCR4-NOT axis dynamically regulates tubulin mRNA levels
- Loss of tubulin autoregulation causes mitosis defects in cells and disease in humans

Höpfler et al., 2023, Molecular Cell *83*, 2290–2302
July 6, 2023 © 2023 MRC Laboratory of Molecular Biology.
Published by Elsevier Inc.
https://doi.org/10.1016/j.molcel.2023.05.020

CellPress

Molecular Cell

Article

Mechanism of ribosome-associated mRNA degradation during tubulin autoregulation

Markus Höpfler,[1] Eva Absmeier,[1] Sew-Yeu Peak-Chew,[1] Evangelia Vartholomaiou,[2] Lori A. Passmore,[1] Ivana Gasic,[2] and Ramanujan S. Hegde[1,3,*]
[1]Medical Research Council Laboratory of Molecular Biology, Cambridge CB2 0QH, UK
[2]Department of Cell Biology, University of Geneva, Geneva, Switzerland
[3]Lead contact
*Correspondence: rhegde@mrc-lmb.cam.ac.uk
https://doi.org/10.1016/j.molcel.2023.05.020

SUMMARY

Microtubules play crucial roles in cellular architecture, intracellular transport, and mitosis. The availability of free tubulin subunits affects polymerization dynamics and microtubule function. When cells sense excess free tubulin, they trigger degradation of the encoding mRNAs, which requires recognition of the nascent polypeptide by the tubulin-specific ribosome-binding factor TTC5. How TTC5 initiates the decay of tubulin mRNAs is unknown. Here, our biochemical and structural analysis reveals that TTC5 recruits the poorly studied protein SCAPER to the ribosome. SCAPER, in turn, engages the CCR4-NOT deadenylase complex through its CNOT11 subunit to trigger tubulin mRNA decay. SCAPER mutants that cause intellectual disability and retinitis pigmentosa in humans are impaired in CCR4-NOT recruitment, tubulin mRNA degradation, and microtubule-dependent chromosome segregation. Our findings demonstrate how recognition of a nascent polypeptide on the ribosome is physically linked to mRNA decay factors via a relay of protein-protein interactions, providing a paradigm for specificity in cytoplasmic gene regulation.

INTRODUCTION

Microtubules (MTs) constitute a fundamental part of the eukaryotic cytoskeleton with key roles in shaping widely varying cellular architectures, in facilitating transport within cells over long distances, and in segregating chromosomes during cell division.[1,2] These functions rely on the highly dynamic assembly and disassembly of MTs from heterodimeric subunits comprising α- and β-tubulins.[1,2] MTs are regulated by more than 40 MT-associated proteins (MAPs) that modify the behavior of individual MTs and their assembly into higher-order structures.[3] Furthermore, tubulins are subject to an extensive range of post-translational modifications, some of them exclusively found on tubulins.[4,5]

Despite research on tubulin and MTs for many decades, several crucial MT regulators have only recently been identified and are often still poorly characterized.[6–8] Many of these are linked to human pathologies, such as cancer and neurodevelopmental or neurodegenerative conditions, and represent potential targets for therapeutics that could complement other tubulin-targeting drugs, such as taxol and colchicine.[8–12] Thus, accurate MT regulation is of exceptionally broad importance, and deciphering the range of pathways that impinge on tubulins is crucial for understanding and modulating the progression of various pathologic states.

A key parameter for the balance between MT growth and shrinkage is the concentration of the free tubulin subunits.[1,13] Several decades ago, it was recognized that cellular tubulin concentration is tightly controlled in part by a feedback mechanism termed tubulin autoregulation.[14–16] This widely conserved phenomenon dynamically adjusts tubulin mRNA levels in response to changes in the level of free tubulin subunits. Regulation occurs strictly post-transcriptionally and involves translation-dependent mRNA degradation that is preferentially triggered under conditions of excess free tubulin. How this highly selective autoregulatory loop operates has long been mysterious.

The only known component in the tubulin autoregulation pathway is tetratricopeptide repeat protein 5 (TTC5), a recently discovered factor that recognizes an N-terminal four amino acid motif common to nascent tubulin polypeptides emerging from translating ribosomes.[17] This motif, either MREI or MREC in β- or α-tubulins, respectively, was shown to be sufficient to trigger degradation of an unrelated mRNA when positioned at the N terminus of the encoded protein.[15,18] Mutations that impair TTC5 recognition of either the tubulin N-terminal motif or the ribosome abolish autoregulation and lead to aberrant mitosis,[17] a highly sensitive measure of perturbed MT dynamics.[19,20] Although the discovery and validation of TTC5 finally provided a molecular handle for the tubulin autoregulation pathway, it is not known why TTC5 binding at the polypeptide exit tunnel of tubulin-producing ribosomes leads to degradation of the associated mRNAs. Furthermore, the broader biological relevance of autoregulation for human physiology is unclear.

 © 2023 MRC Laboratory of Molecular Biology. Published by Elsevier Inc.
This is an open access article under the CC BY license (http://creativecommons.org/licenses/by/4.0/).

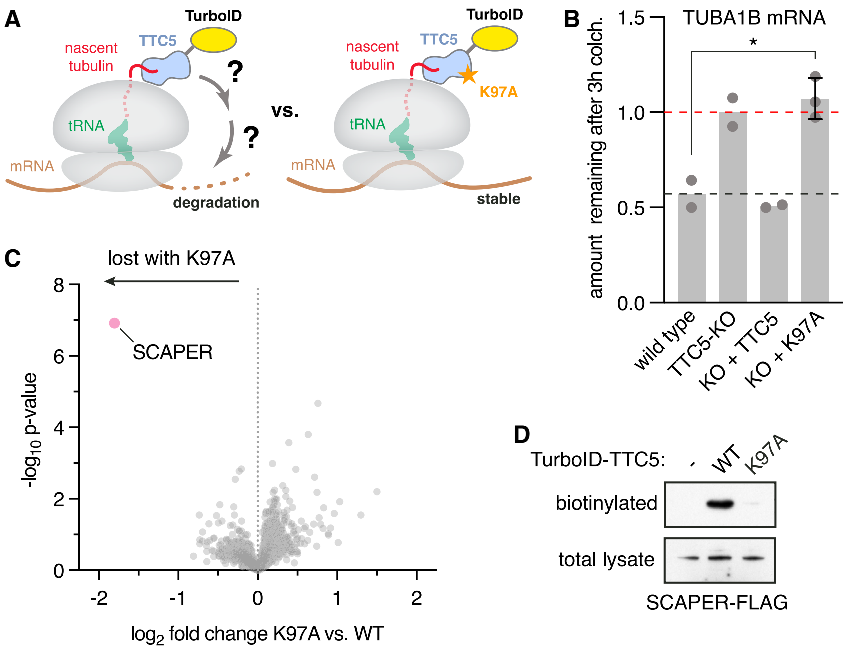

Figure 1. TTC5 proximity labeling identifies SCAPER as autoregulation-specific interactor

(A) Strategy for identification of tubulin autoregulation factors acting downstream of TTC5 on tubulin-translating ribosomes. Proximity labeling was achieved by fusing TurboID to the N terminus of either wild-type (WT) TTC5 or the Lys^{97} → Ala (K97A) mutant.

(B) Quantification of tubulin mRNA in HEK293 T-REx cells by reverse transcription followed by quantitative real-time PCR. TUBA1B mRNA levels were normalized to a house-keeping gene (RPLP1) and the relative amount remaining after 3 h 10 μM colchicine (colch.) treatment is plotted. This is hereafter referred to as the "autoregulation assay." The red dashed line indicates the starting tubulin mRNA level prior to colchicine, arbitrarily set to a value of 1. The black dashed line indicates the amount remaining in WT cells. This is typically ~0.5 after 3 h of colchicine, reflective of 50% mRNA degradation, but varies slightly in different experiments due to minor variations in experimental conditions. TTC5 knockout (KO) was complemented by re-expressing GFP-tagged WT or K97A TTC5. Data show the mean from 2 independent experiments, one of which contained 2 replicates for the TTC5 K97A cell line. Error bars denote standard deviation (SD). The lack of TUBA1B mRNA degradation in the K97A cell line relative to WT cells was statistically significant (asterisk, $p = 0.014$, Student's t test).

(C) Proximity labeling using TurboID fused to either WT or mutant (K97A) TTC5 followed by enrichment of biotinylated proteins and quantitative mass spectrometry. 6 samples were analyzed for TurboID-TTC5 WT and K97A. See also Table S2.

(D) Proximity labeling assay as in (C) with overexpression of FLAG-tagged SCAPER in the indicated cell lines. Total lysates were probed with anti-FLAG antibody and the biotinylated population with anti-SCAPER antibody. Endogenous SCAPER is not detected at this exposure due to its low expression. HEK293 T-REx cells were used for all cell-based assays in this study, unless stated otherwise (Figures 6 and S9). See also Figure S1.

Although multiple cases of mRNA sequence-dependent post-transcriptional regulation have been well characterized,[21–23] the molecular basis for the coupling of nascent chain recognition to selective mRNA degradation is poorly understood. Prominent examples of such nascent peptide-dependent regulation include highly expressed mRNAs, such as those coding for endoplasmic reticulum (ER)-targeted proteins[24,25] and ribosomal proteins.[26] Recent studies suggest that bacteriophage-derived anti-CRISPR proteins recognize nascent Cas12a protein to trigger the degradation of Cas12a mRNA, suggesting that related mechanisms exist beyond eukaryotes.[27,28]

Conceptually, specific nascent peptide recognition coupled to mRNA decay is reminiscent of the well-studied co-translational capture of signal sequences by the signal recognition particle, which targets ribosome-nascent-chain (RNC) complexes to the ER membrane. Similarly, RNCs can be used to direct mRNAs to other locations, such as centrosomes, apical poles in epithelial cells during embryogenesis, and others,[29–32] or to drive mRNA co-localization for co-translational protein complex assembly.[33,34] These examples illustrate that nascent, chain-directed mRNA fate decisions are broadly relevant, but the molecular mechanisms and structural features linking peptide recognition to downstream events are enigmatic in most cases. Given the critical functions of MTs in numerous areas of cell and organism homeostasis,[19,35] neuronal cell function,[12] and their relevance as drug targets,[9,10] we sought to understand the mechanistic basis of co-translational mRNA decay using tubulins as an example.

RESULTS

TTC5 recruits SCAPER to ribosomes

Tubulin autoregulation can be experimentally induced by MT depolymerizing drugs, such as colchicine or combretastatin A4 (CA4). The acute rise in free tubulin heterodimers comprising α and β subunits liberates TTC5 from a yet-unidentified sequestration factor.[17] TTC5 then engages tubulin-synthesizing ribosomes and triggers degradation of tubulin mRNAs to ~50% of starting levels after 3 h. We quantify this acute degradation in autoregulation assays throughout this study. How TTC5 leads to mRNA degradation is unknown.

To identify factors downstream of ribosome-bound TTC5, we used a biotin proximity labeling strategy.[36] The promiscuous biotin ligase TurboID was fused to TTC5 to biotinylate interaction partners during ongoing tubulin mRNA degradation (Figure 1A). As a specificity control, we sought a TTC5 mutant that is competent for recognition of tubulin RNCs but fails to effect downstream mRNA degradation. We noticed that a highly conserved surface patch around K97 does not interact with either the ribosome or nascent tubulin (Figure S1A), suggesting that it might recruit downstream factors. Consistent with this idea, TTC5 with the K97A mutation abolished TTC5's capacity to trigger tubulin mRNA degradation (Figures 1B and S1B) despite

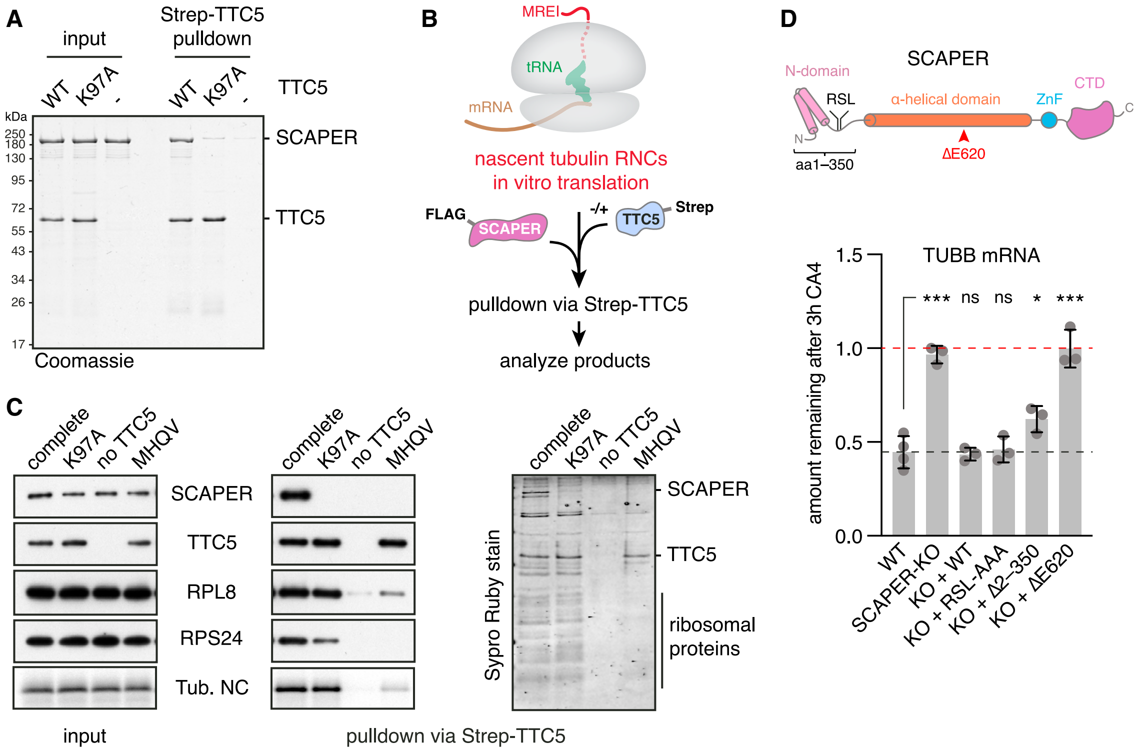

Figure 2. TTC5 recruits SCAPER to tubulin ribosome-nascent-chain complexes for autoregulation

(A) Recombinant Strep-TTC5 and SCAPER-FLAG were incubated together and pulled down via Strep-TTC5. Bound proteins were separated using SDS-PAGE and visualized by Coomassie staining.

(B) Schematic workflow for reconstitution of SCAPER recruitment to tubulin ribosome nascent chains (RNCs) via TTC5 as shown in (C).

(C) 64-residue β-tubulin (TUBB) nascent chains were produced in rabbit reticulocyte lysates in the presence of recombinant FLAG-SCAPER (all samples) and Strep-TTC5 as indicated, and TTC5-associated proteins were subsequently enriched via its Strep tag. Input and Strep-TTC5 pull-down samples were separated by SDS-PAGE and visualized by western blotting, autoradiography for the β-tubulin nascent chain (Tub. NC), or SYPRO Ruby staining for total protein. "MHQV" indicates a β-tubulin construct in which its TTC5-interacting MREI motif has been mutated.

(D) Top: schematic of SCAPER domain architecture, including annotated features and predicted structural elements. The pathologic ΔE620 mutation is indicated by a red arrowhead (see also Figure S4A). RSL, cyclin A-binding motif (Arg^{199}-Ser^{200}-Leu^{201}); ZnF, zinc finger; CTD, carboxy-terminal domain. Bottom: autoregulation assay with HEK T-REx wild type, SCAPER-KO (sgRNA1 cl. 1), and the indicated FLAG-SCAPER rescue cell lines. RSL-AAA: mutation of the cyclin A-binding site (Arg^{199}-Ser^{200}-Leu^{201}) to alanines; Δ2–350: deletion of residues 2–350; ΔE620: deletion of residue Glu^{620}. Data show the mean ± SD from 2 independent experiments, one of which contained 2 replicates. Single asterisk indicates $p < 0.05$, triple asterisk indicates $p < 0.001$, and "ns" indicates not significant. See Figures S3B–S3D for a detailed analysis of SCAPER-KO cell lines. The same SCAPER-KO cell line (sgRNA1 clone 1) was used for complementation assays throughout the rest of the study. See also Figures S2–S4.

normal expression (Figure S1C) and unimpaired recruitment to tubulin RNCs (Figure S1D).

After confirming that the TurboID-TTC5 fusion reconstitutes autoregulation in a TTC5 knockout (KO) cell line and that the TurboID-TTC5(K97A) mutant is ineffective (Figure S1E), we induced biotinylation during active autoregulation (Figure S1F) and affinity purified the biotinylated proteins (Figure S1G). Quantitative mass spectrometry revealed that a poorly studied protein named SCAPER (S-phase cyclin A associated protein residing in the ER) was the only protein whose biotinylation was strongly reduced in TTC5(K97A) cells (Figure 1C). Notwithstanding its name, SCAPER lacks obvious ER-targeting domains and is nucleo-cytoplasmic, as determined by immunostaining.[37] Immunoblotting verified that in cells, SCAPER is biotinylated by TurboID-TTC5 in a K97-dependent manner (Figure 1D).

In pull-down experiments, purified SCAPER interacted with purified TTC5 but not TTC5(K97A) (Figure 2A). Structure modeling using AlphaFold2 (AF2) multimer[38,39] predicted a high-confidence interaction between the region of TTC5 that contains K97 and a globular C-terminal domain (CTD) of SCAPER (Figures S2A and S2B). In a cytosolic *in vitro* translation reaction, recombinant SCAPER co-fractionated and co-purified with TTC5-RNC complexes displaying the first 64 amino acids of β-tubulin (Figures 2B, 2C, and S2C). This interaction was not seen in reactions containing TTC5(K97A), reactions lacking β-tubulin RNCs, or reactions containing RNCs with mutant β-tubulin incapable of TTC5 recruitment. Thus, SCAPER is selectively recruited to tubulin-synthesizing ribosomes via a direct interaction with TTC5.

SCAPER is required for autoregulation

Cells knocked down or knocked out for SCAPER are completely deficient in tubulin autoregulation (Figures 2D and S3A–S3D). Tubulin mRNAs decay exponentially after MT depolymerization

in wild-type (WT) cells with half-live times of ~2.2–2.6 h but were stable for 6 h in SCAPER-KO cells (Figure S3E). This phenotype can be fully rescued by SCAPER reintroduction (Figure 2D). Domain mapping experiments showed that the N-terminal part, which contains the SCAPER N-domain and a previously characterized cyclin A-binding site,[40] is largely dispensable for autoregulation (construct Δ2–350 in Figures 2D and S4A–S4C). Consistent with this result, a cyclin A-binding mutant (RSL-AAA) had no effect on autoregulation (Figure 2D). By contrast, SCAPER constructs Δ2–700 (which additionally deletes the central α-helical domain) and Δ936–1,400 (which lacks most of the CTD) were completely inactive in restoring autoregulation to KO cells (Figures S4B and S4C). This suggests that both the central α-helical domain and the CTD are required for tubulin autoregulation.

Interestingly, numerous disease-linked SCAPER mutations cause C-terminal truncations or are located in the central and CTDs (Figure S4A).[41,42] These mutations lead to retinitis pigmentosa, intellectual disability, male infertility, and other pathologies consistent with MT cytoskeleton aberrations.[42–44] Given the lack of complementation of SCAPER disease variants truncated after codons 726 or 935 (Figure S4B), pathological truncations further upstream in the protein are presumably also non-functional for autoregulation.

Furthermore, two disease-causing deletion mutants in the central α-helical domain (ΔE620 and Δ675–677) led to severe autoregulation defects without appreciably affecting SCAPER expression (Figures 2D, S4D, and S4E), whereas a third disease allele (S1219N) was expressed at substantially lower levels (Figure S4E), presumably due to destabilization of the protein. Notably, restoring the α-helix register to ΔE620 by inserting an alanine at this site (E620A) restored SCAPER function in autoregulation assays (Figures S4D and S4E). Thus, SCAPER alleles that cause human disease are impaired in tubulin autoregulation, highlighting key roles for the autoregulation pathway in human physiology.

Mechanism of ribosome engagement by SCAPER

To understand how SCAPER binds tubulin-synthesizing ribosomes, we analyzed β-tubulin-RNCs engaged with recombinant TTC5 and SCAPER (Figure 2C) by single-particle cryoelectron microscopy (cryo-EM). The structure, at an overall resolution of 2.8 Å and local resolution from 3 to 8 Å for non-ribosomal regions (Table S1), showed that SCAPER's CTD makes contacts with TTC5, the 60S surface, and an additional density that was identified as the 28S rRNA expansion segment ES27L (Figures 3A and S5). The other parts of SCAPER upstream of residue 859 were not resolved. AF2 models of TTC5 with the tubulin nascent chain and the SCAPER-CTD were docked into the cryo-EM map and adjusted to generate a structural model.

In this model, K97 of TTC5 is positioned near a negatively charged and highly conserved surface patch on SCAPER around E1338, explaining why the K97A mutation is defective in SCAPER interaction and autoregulation (Figure 3B). Furthermore, two conserved positively charged surface patches on SCAPER contact the 60S subunit and ES27L (Figure S6A). At the 60S interface, R907 and K910 of SCAPER abut 28S rRNA residues U2707-C2709, and R934 and R941 of SCAPER interact with D145 and D148 of ribosomal protein uL23 (Figures 3C and 3D, respectively). At the ES27L interface, a cluster of eight conserved positively charged residues between K867 and R878 along an α-helix from SCAPER faces rRNA (Figure S6B), although details of this interaction were not visualized at the moderate resolution in this part of the map. The function of rRNA expansion segments is poorly understood, but ES27L emerges as a key structural element that is known to scaffold binding of factors around the exit tunnel for various functions.[45–47]

SCAPER variants with point mutations at the interaction sites with TTC5, the 60S body, and ES27L each failed to restore autoregulation to SCAPER-KO cells (Figures 3E and 3F) despite high expression levels (Figures S6C and S6D). The charge reversal mutation E1338K in SCAPER, opposite to K97 in TTC5, strongly affected autoregulation. Similarly, a triple alanine mutation of E1338, M1339, and S1340 (EMS-AAA) on the SCAPER surface that forms the primary TTC5 binding site was completely inactive. Finally, mutants of conserved positively charged SCAPER residues that contact either the 60S body rRNA (R907E, K910E), uL23 (R934A, R941A), or ES27L (ES*-4E or -7E) were inactive. Thus, SCAPER uses its CTD to selectively engage TTC5-containing ribosomes through three crucial contacts. The structure explains why all disease-causing premature termination codons in SCAPER (Figure S4A), even those close to the C terminus, would be incompatible with SCAPER recruitment by TTC5. Furthermore, the region N-terminal to the ribosome-binding CTD would extend toward the 40S subunit and potentially reach over 300 Å (Figure S4A). This is noteworthy because SCAPER would be long enough to bridge the distance from the polypeptide exit tunnel, where TTC5 binds the nascent chain, to the 40S subunit through which the mRNA is threaded.

SCAPER recruits CCR4-NOT for mRNA deadenylation

Because SCAPER has no apparent catalytic domains that would degrade mRNA, we speculated it acts as an adaptor that recruits a nuclease. The absence of a nuclease in our TTC5-centered proximity labeling experiment hinted that distal regions of SCAPER too far for proximity biotinylation might mediate nuclease recruitment. We therefore repeated the experiment with TurboID fused to the N terminus of SCAPER (Figure 4A). The set of biotinylated proteins recovered from cells that are acutely degrading tubulin mRNA, relative to cells at steady state, was enriched for multiple subunits of the CCR4-NOT deadenylase complex (Figure S7A). Strikingly, biotinylated CCR4-NOT subunits were strongly de-enriched in samples from cells expressing SCAPER (ΔE620), a pathologic mutant defective in tubulin autoregulation (Figures 2D and 4B). Thus, CCR4-NOT is proximal to SCAPER's N terminus preferentially during autoregulation conditions of active tubulin mRNA degradation.

The CCR4-NOT complex is a large multi-subunit complex (Figure 4C) responsible for most cytoplasmic deadenylation activity, the first and often rate-limiting step in mRNA decay.[23,48,49] siRNA-mediated knockdown (KD) of CNOT1, the large scaffolding subunit around which all other subunits assemble, completely abolished tubulin mRNA degradation in autoregulation assays (Figures 4D and S7B). Similarly, KD of all four partially redundant nuclease subunits (CNOT6, CNOT6L, CNOT7, and

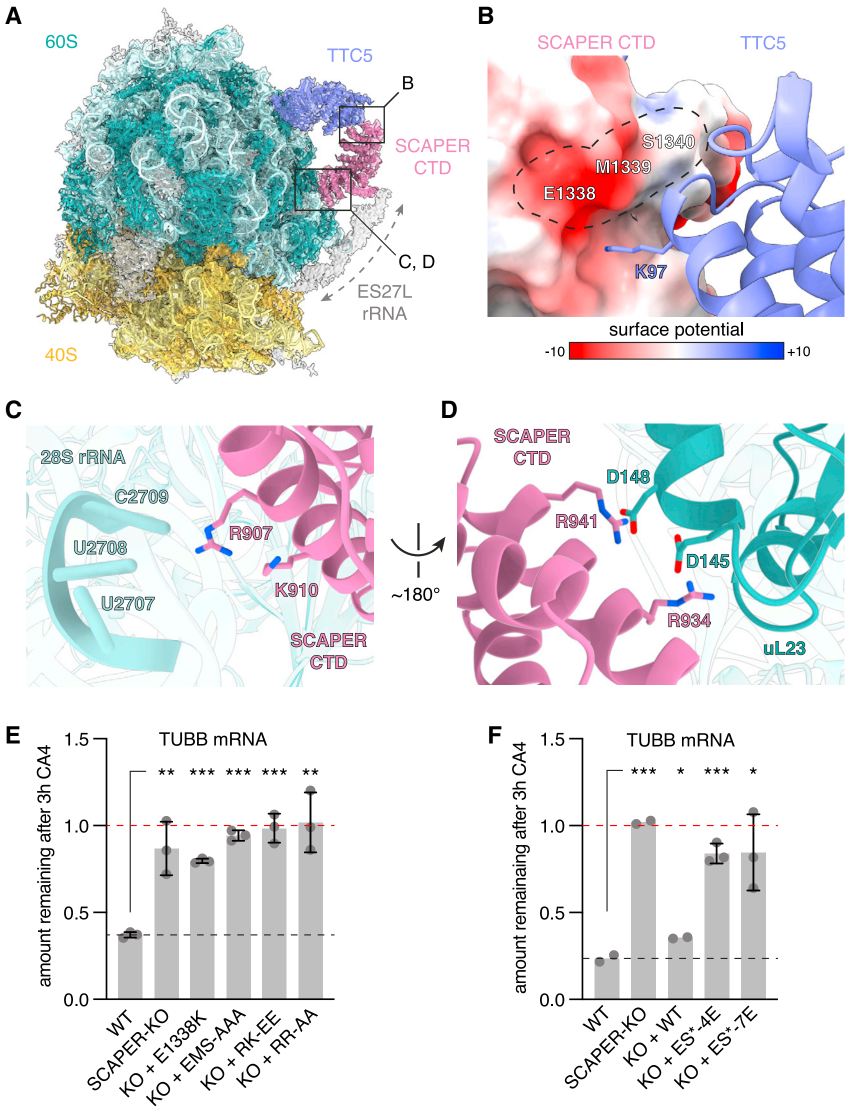

Figure 3. Mechanism of SCAPER recruitment to tubulin RNCs via TTC5

(A) Overview of the cryo-EM-derived structure of β-tubulin-synthesizing ribosomes bound to TTC5 and SCAPER. Dashed arrow marks density that was identified as 28S rRNA expansion segment ES27L. Boxes indicate positions of close-ups shown in (B)–(D). The displayed non-sharpened map resulted from the ES27L classification (see Figure S5). The 40S subunit was rigid-body docked and is shown to orient the reader.

(B) Close-up view of the contact between SCAPER and TTC5. SCAPER is colored by electrostatic surface potential [in kcal/(mol * *e*)], and the surface area of critical residues is outlined.

(C) Close-up view of critical positively charged SCAPER residues in close vicinity to 28S rRNA.

(D) Close-up view of conserved arginine residues of SCAPER in close proximity to aspartate residues of ribosomal protein uL23.

(E) Autoregulation assay comparing WT, SCAPER-KO, and rescue cell lines expressing the indicated SCAPER mutants. EMS-AAA: E1338A, M1339A, S1340A; RK-EE: R907E, K910E; RR-AA: R934A, R941A. Data show the mean ± SD from 2 independent experiments, one of which contained 2 replicates.

(F) Autoregulation assay as in (E) with mutations in the ES27L contact site of SCAPER. ES*-4E: K867E, K870E, K873E and K874E; ES*-7E: as ES*-4E plus K869E, K871E and R878E. See also Figure S6. Data show the mean from 2 independent experiments, one of which contained 2 replicates for each of the key mutants. Error bars denote SD. Single, double, and triple asterisks indicate $p < 0.05$, $p < 0.01$, and $p < 0.001$, respectively. See also Figures S5 and S6 and Table S1.

CNOT8) also stabilized tubulin mRNAs (Figures 4E and S7C). This defect was rescued by re-expressing siRNA-resistant CNOT8, but not by a catalytic dead mutant (Figures 4E and S7C).

Because CCR4-NOT is an exonuclease with specificity for poly(A), its requirement suggested that SCAPER-triggered tubulin mRNA decay is initiated by deadenylation. To test this hypothesis directly, we performed poly(A) tail-length assays.[50] The poly(A) tail of TUBA1B was noticeably shortened in WT cells after initiating autoregulation by MT depolymerization (Figure 4F). Autoregulation-triggered shortening of the poly(A) tail was abolished in SCAPER-KO cells but could be rescued by re-expressing WT SCAPER. The poly(A) tail length of glyceraldehyde-3-phosphate dehydrogenase (GAPDH) mRNA was not affected by either MT depolymerization or SCAPER expression. Thus, the CCR4-NOT complex is physically proximal to SCAPER during autoregulation and its exonuclease activity is required to initiate tubulin mRNA degradation by deadenylation.

Mechanism of CCR4-NOT recruitment by SCAPER

CNOT1 not only scaffolds the catalytic exonuclease subunits but also regulatory subunits that deploy the CCR4-NOT complex to specific mRNAs via RNA-binding adaptor proteins.[23,49] An initial screen of CCR4-NOT subunits by siRNA-mediated KD revealed that CNOT10 and CNOT11 are most important for tubulin autoregulation (Figure 5A; Figure S7D). The specificity of this effect was underscored by the finding that several other previously described CCR4-NOT substrates were stabilized by CNOT1 KD but not by KD of CNOT10 or CNOT11 (Figure 5B), consistent with previous findings.[51] CNOT10 interacts with CNOT11 to form a module that evolved later than the core CCR4-NOT complex, similar to the evolution of other tubulin autoregulation components.[16,52] This suggested that the CNOT10/CNOT11 module, although dispensable for some other CCR4-NOT functions, might recognize SCAPER for recruitment to tubulin RNCs.

Other subunits of CCR4-NOT, such as the CNOT2/CNOT3 module and CNOT9, interact with substrate-specific RNA-binding proteins that act as adaptors for selective mRNA decay. Speculating that SCAPER might be a substrate-specific adaptor for the CNOT10/CNOT11 module, we screened for potential

CellPress
OPEN ACCESS

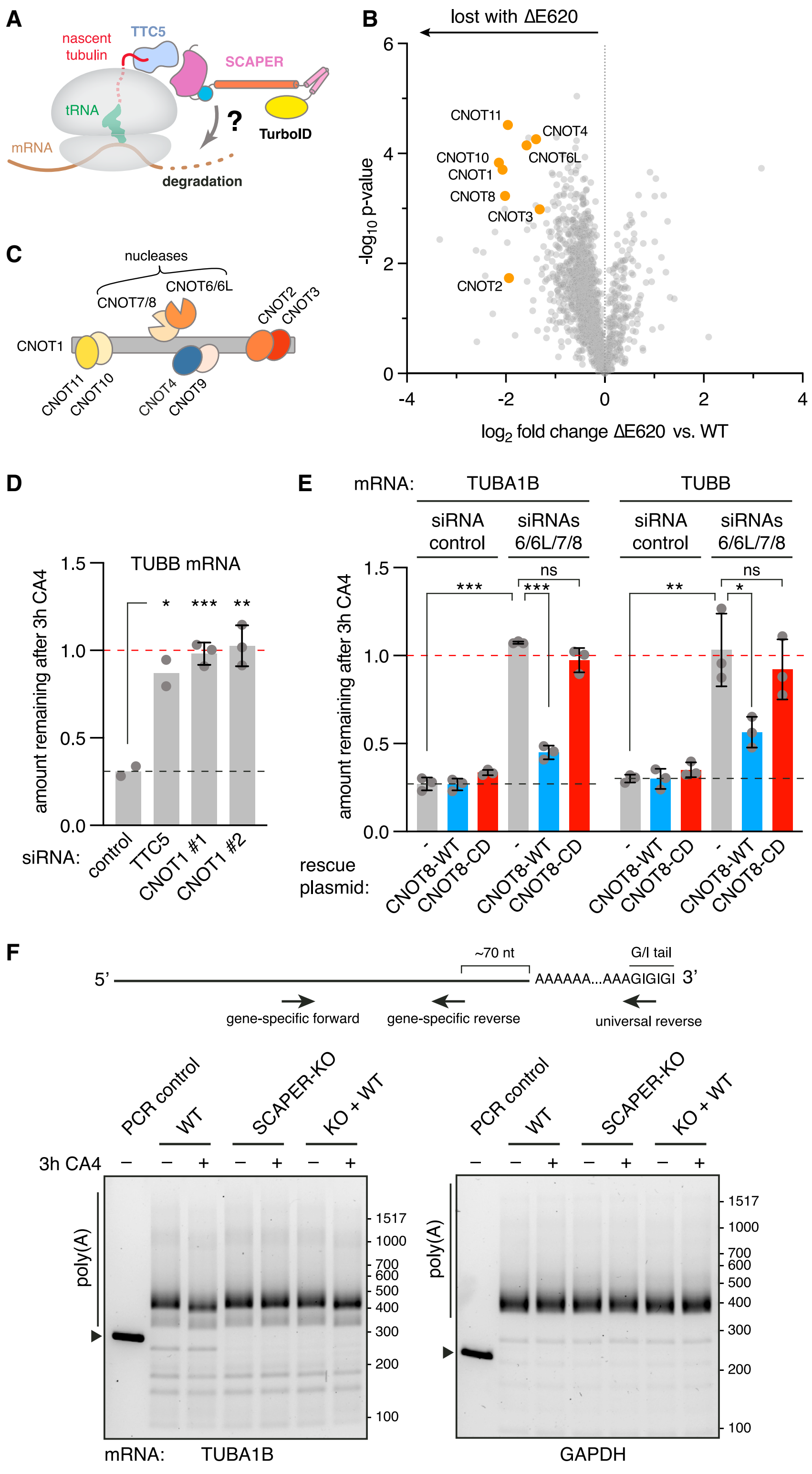

Figure 4. The CCR4-NOT complex triggers tubulin mRNA degradation

(A) Schematic for the strategy to identify factors acting downstream of SCAPER to degrade tubulin mRNA.

(B) Proximity labeling in HEK T-REx cells using TurboID fusions to either SCAPER WT or the ΔE620 mutant, performed after induction of autoregulation with the microtubule depolymerization agent combretastatin A4 (CA4, at 100 nM). Biotinylated proteins were enriched and analyzed by quantitative mass spectrometry. See also Table S3. 3 replicate samples were analyzed for SCAPER WT, and 2 replicates for ΔE620.

(C) Schematic of the subunit composition of the CCR4-NOT complex.

(D) Autoregulation assay performed after knockdowns (KD) using control, TTC5- or CNOT1-targeted siRNAs. Data show the mean from 2 independent experiments, one of which contained 2 replicates for CNOT1 siRNAs. Error bars denote SD.

(E) Autoregulation assays performed after control KD, or KD of all partially redundant catalytic deadenylase subunits of CCR4-NOT (siRNAs 6/6L/7/8). CNOT8 expression was accomplished by stable integration of siRNA-resistant CNOT8-WT (blue bars) or the catalytic dead D40A mutant (CNOT8-CD, red bars). Data show the mean ± SD from 2 independent experiments, one of which contained 2 replicates. Single, double, and triple asterisks indicate $p < 0.05$, $p < 0.01$, and $p < 0.001$, respectively; "ns" indicates not significant.

(F) Poly(A) tail-length assays were performed on total RNA isolated from the indicated HEK T-REx cell lines in control conditions or after 3 h 100 nM CA4 treatment to induce tubulin autoregulation. Total mRNAs were modified at their 3′ ends with a guanosine/inosine tail (G/I tail), reverse transcribed, and PCR amplified using a gene-specific forward primer to either TUBA1B (left) or GAPDH (right) and universal reverse primer. Size markers for PCR products lacking a poly(A) tail were generated using gene-specific reverse primers that anneal in the 3′ UTRs ~70 nt upstream of the poly(A)-site (first lane of each gel, marked by triangles). PCR products were separated on agarose gels and inverted images are shown. Diagram depicts the PCR strategy and positions of primers. See also Figure S7.

interactions with regions of SCAPER using AF2 multimer.[38,39] A high-confidence interaction was predicted between the highly conserved C-terminal DUF2363 domain of CNOT11 and conserved residues of the SCAPER α-helical domain (Figures 5C and S8). Strikingly, E620 of SCAPER was adjacent to the CNOT11 binding surface (but not in direct contact), perhaps explaining why a shift of α-helix register in this region caused by the ΔE620 mutation abolishes autoregulation and causes disease (Figures 2D, 5C, and S8C).

Guided by the AF2 prediction, we designed mutations on either side of the SCAPER-CNOT11 interface and introduced them into the respective KO cell line. Whereas the WT constructs rescued the KO phenotype, each of the interface mutants was completely deficient for autoregulation (Figures 5D, 5E, S7E, S7F, S8C, and

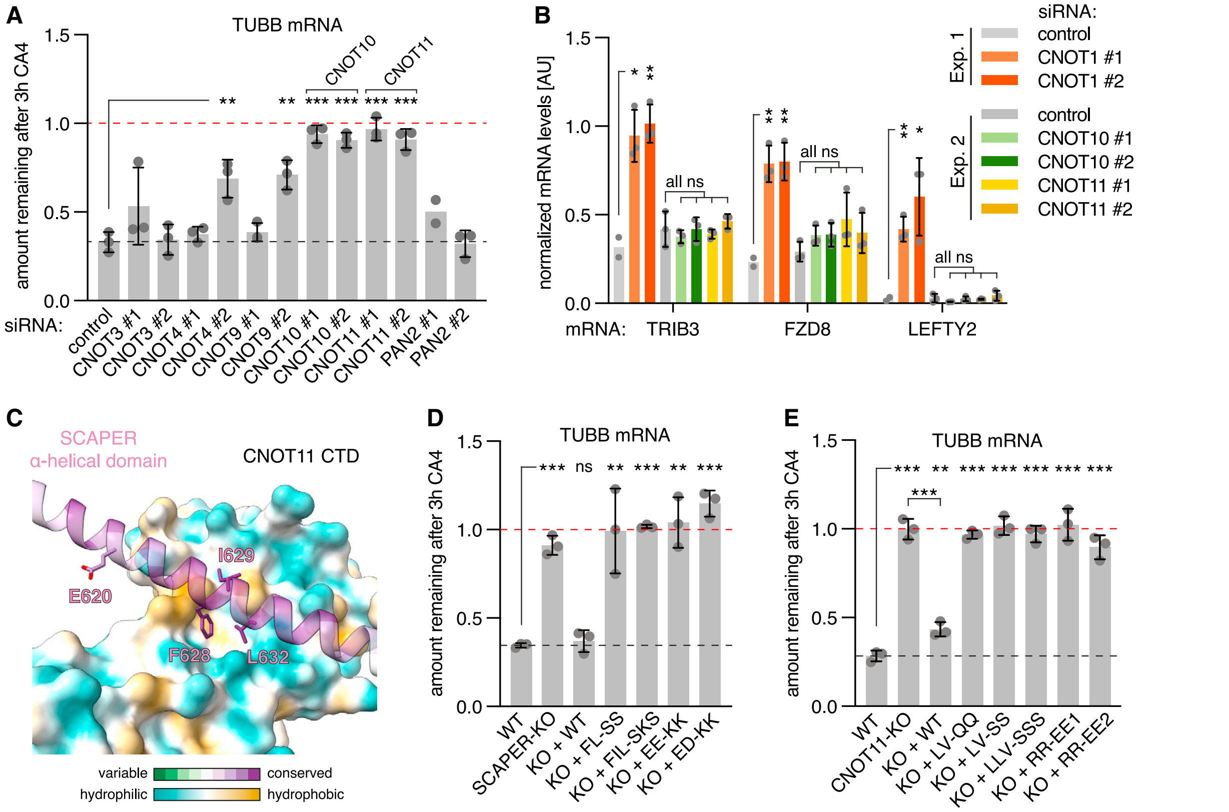

Figure 5. SCAPER recruits the CCR4-NOT complex via CNOT11

(A) Autoregulation assays were performed after KD using the indicated siRNAs for 3–4 days. We note that KD of PAN2 did not lead to stabilization of tubulin mRNAs in autoregulation assays. PAN2 is the catalytic subunit of the PAN2-PAN3 complex that often initiates deadenylation before CCR4-NOT.[23] Data show the mean ± SD from 3 independent experiments. One sample for siPAN2 #1 was lost. Significant changes from the siRNA control condition are indicated by asterisks.

(B) Real-time quantitative PCR quantification of previously identified CCR4-NOT substrates[53,54] in samples with KD for CNOT1, CNOT10, or CNOT11. The same samples from control conditions used in Figure 4D (Exp. 1) and (A) (Exp. 2) were analyzed. Target mRNA levels were normalized to a house-keeping gene (GAPDH). Normalization to 18S rRNA, which is not a deadenylation substrate of CCR4-NOT, gave comparable results. Note that LEFTY2 mRNA levels were at or below the detection threshold for all samples except CNOT1-KD samples. For Exp. 1, data show the mean ± SD from 2 independent experiments, one of which contained 2 replicates for CNOT1 siRNAs. For Exp. 2, data show mean ± SD from 3 independent experiments.

(C) Model of AlphaFold2 multimer predicted interaction between the C-terminal domain of CNOT11 with the α-helical domain of SCAPER. E620 and three highly conserved hydrophobic SCAPER residues predicted to interact with a hydrophobic patch on CNOT11 are highlighted.

(D) Autoregulation assay with WT, SCAPER-KO, or SCAPER rescue cell lines with the indicated mutations targeting the predicted CNOT11 interaction surface. FL-SS: F628S, L632S; FIL-SKS: F628S, I629K, L632S; EE-KK: E618K, E625K; ED-KK: E633K, D640K

(E) Autoregulation assay with WT, CNOT11-KO, or CNOT11 rescue cell lines with the indicated mutations targeting the predicted SCAPER interaction surface. LV-QQ: L405Q, V454Q; LV-SS: L405S, V454S; LLV-SSS: L405S, L451S, V454S; RR-EE1: R447E, R450E; RR-EE2: R461E, R485E. For (D) and (E), data show the mean ± SD from 2 independent experiments, one of which contained 2 replicates. Single, double, and triple asterisks indicate $p < 0.05$, $p < 0.01$, and $p < 0.001$, respectively; "ns" indicates not significant. See also Figures S7 and S8.

S8D), validating key features of the AF2-predicted interaction. Moreover, a recently published crystal structure of the isolated CNOT11 CTD closely matches our AF2-predicted structure (PDB: 8BFH).[55] Taken together, our data imply that the CCR4-NOT complex employs its CNOT10/CNOT11 module to selectively engage tubulin RNCs marked by the TTC5-SCAPER complex via nascent chain recognition. At these RNCs, the nuclease subunits of CCR4-NOT can deadenylate tubulin mRNAs to trigger their degradation during autoregulation.

SCAPER mutation causes mitosis defects

Accurate regulation of tubulin levels is crucial for MT-dependent processes, including the formation of the mitotic spindle during cell division. To investigate the relevance of SCAPER-dependent autoregulation during mitosis in a cell-based assay, we monitored chromosome segregation using live cell microscopy (Figure 6A). We found that SCAPER-KO cells have a ~4-fold increase in chromosome alignment and segregation errors (Figures 6B, 6C, and S9A–S9C) similar to the effects seen in TTC5-KO cells.[17] Neither the cyclin A binding site mutation (RSL-AAA) nor truncation of the N terminus showed this phenotype. By contrast, the ΔE620 disease mutant, which is deficient in CCR4-NOT recruitment, essentially phenocopied the SCAPER-KO (Figures 6B, 6C, and S9A–S9C). These outcomes closely correlate with the phenotypes in tubulin autoregulation assays of the respective genotypes (Figures 2D and S9B).

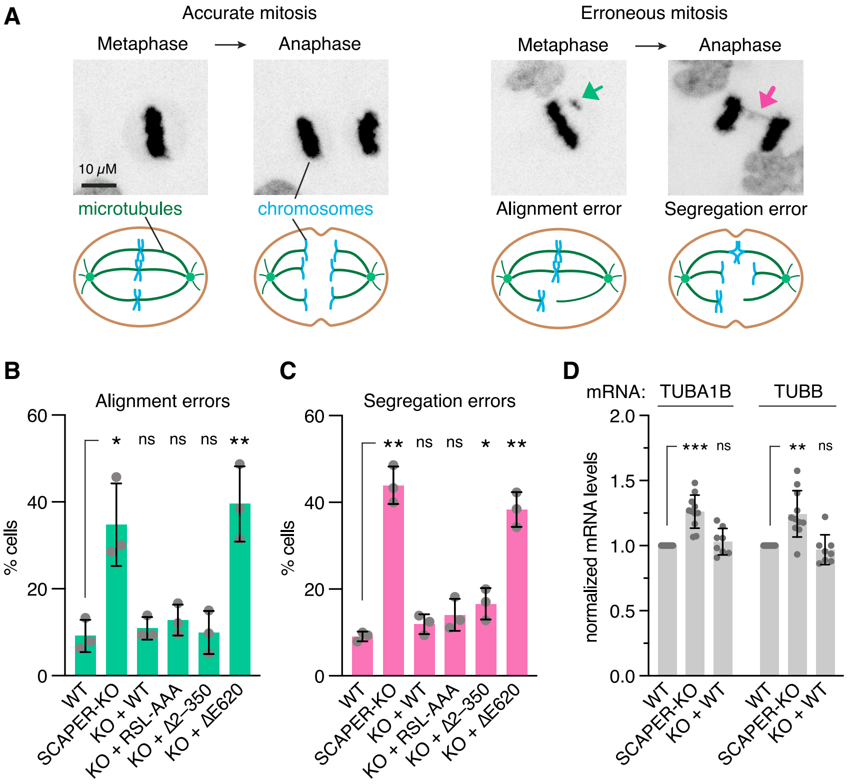

Figure 6. SCAPER is required for accurate mitosis

(A) Example images of meta- and anaphase stages of HeLa cells going through mitosis in which chromosomes were visualized using SiR-DNA stain and maximum intensity projections are shown. Misaligned chromosomes and segregation errors are highlighted by green and magenta arrows, respectively. Schematics of accurate and erroneous cell division stages are shown below images. Chromosomes are shown in blue, MTs in dark green, centrosomes in light green.

(B) Quantification of chromosome alignment errors in stable Flp-In HeLa T-REx cell lines with the indicated genotypes.

(C) Quantification of chromosome segregation errors in stable Flp-In HeLa T-REx cell lines with the indicated genotypes. Data show mean ± SD from three independent experiments with 100 cells in total for (B) and (C). Unpaired, two-tailed Student's t tests were performed for each of the indicated cell lines with the WT cell line as reference. Single, double, and triple asterisks indicate $p < 0.05$, $p < 0.01$, and $p < 0.001$, respectively; "ns" indicates not significant.

(D) Quantification of steady-state tubulin mRNA levels in the indicated HEK T-REx cell lines. Tubulin mRNA levels were normalized to a reference gene (RPLP1) and to the WT cell line, and data from all relevant experiments in the manuscript were compiled. Data show mean ± SD. Statistical analysis for SCAPER-KO and rescue cell lines was performed using a one-sample t test. Values significantly different from 1 (WT levels) are indicated by double and triple asterisks ($p < 0.01$, and $p < 0.001$, respectively; "ns" indicates not significant). n = 11 for WT and SCAPER-KO, n = 8 for KO + WT; data reanalyzed from Figures 3E, 3F, 5D, and S4D. See also Figure S9.

Consistent with a function of SCAPER in ensuring accurate cellular tubulin levels, we found that KO of SCAPER led to ~25% increased steady-state tubulin mRNA levels, independent of drug-induced MT depolymerization (Figure 6D). Similar effects were seen for cells lacking TTC5 or CNOT11 (Figures S9D and S9E), indicating that tubulin autoregulation is needed for maintaining tubulin homeostasis even under normal conditions. In support of this, a recent high-throughput microscopy study found tubulin protein levels elevated upon CCR4-NOT disruption, specifically when CNOT1, CNOT10, or CNOT11 were depleted.[56] Thus, tubulin autoregulation has a house-keeping function during normal cell growth to rein in tubulin expression and ensure faithful mitosis. The observed mitosis defects seen in the absence of autoregulation are expected to result in aneuploidy, which is associated with cancer progression and can impair neurodevelopment.[57–59]

DISCUSSION

The mechanistic basis for selective tubulin mRNA degradation and its physiological function have been long-standing questions since the description of tubulin autoregulation more than 40 years ago.[14] In this work, we elucidated the factors and interactions that bridge nascent tubulin peptide recognition at the ribosome exit tunnel to mRNA deadenylation (Figure 7). The findings assign molecular functions to the previously obscure proteins SCAPER and CNOT11, provide mechanistic insight into genetic diseases caused by SCAPER mutations, and provide a detailed view of how a nascent protein can selectively control the degradation of its encoding mRNA. The work therefore highlights several principles in post-transcriptional gene regulation.

The most noteworthy insight to emerge from our studies is the mechanistic basis for how an mRNA can be targeted for selective degradation by direct recognition of the nascent protein. Instead of sequence-specific recognition of tubulin mRNAs, a series of protein-protein interactions at the translating ribosome culminates in the recruitment of a general deadenylase complex. A major advantage of this mechanism is that an entire class of mRNAs, the α- and β-tubulins totaling 18 genes in humans, can be targeted as a group despite widely varying UTRs and coding sequences. Instead, they are recognized via a shared peptide motif in the proteins they encode. This is conceptually analogous to how a single microRNA can coordinately regulate multiple widely different proteins based on a shared recognition motif in their encoding mRNAs.[22]

In the autoregulation pathway, TTC5 imparts specificity for tubulins and contributes decisively to the specificity of SCAPER recruitment. Because SCAPER has the potential to be highly elongated, the CNOT11 binding site can reach far from the polypeptide exit tunnel where its CTD engages TTC5. Consistent with this idea, cross-linking mass spectrometry

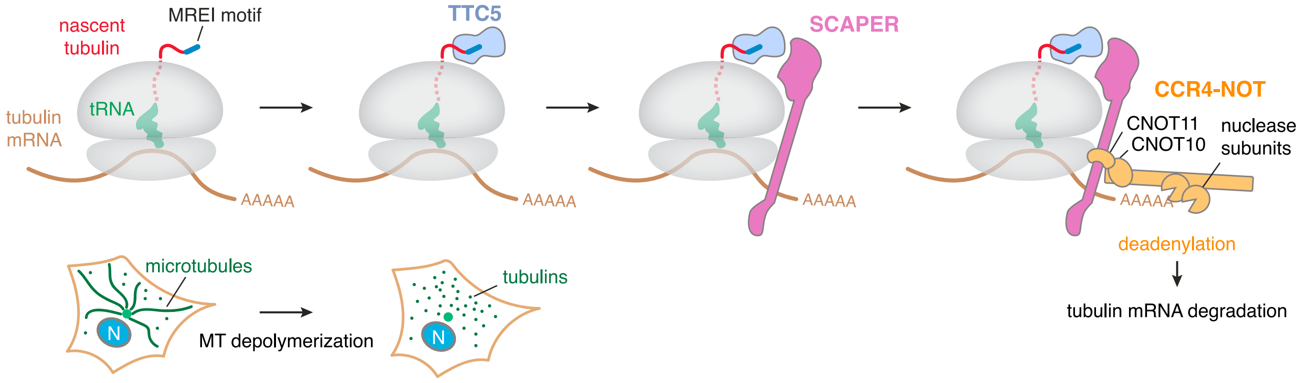

Figure 7. Model of regulated mRNA degradation in the tubulin autoregulation pathway

Selective tubulin mRNA degradation is triggered when cells sense excess free tubulin levels, e.g., due to microtubule (MT) depolymerization, as depicted in the bottom schematic (N: nucleus). Under these conditions, TTC5 is liberated from an elusive inhibitory factor[17] (not shown). This allows TTC5 to selectively bind tubulin-translating ribosomes by interacting with the conserved N-terminal peptide motif (Met-Arg-Glu-Ile or MREI, shown in dark blue) and a surface around the ribosomal exit tunnel. SCAPER recruitment is, in turn, facilitated by a composite interaction surface formed by TTC5 and the ribosome. The CCR4-NOT complex uses its CNOT11 subunit to bind an extended α-helical domain of SCAPER and its nuclease subunit(s) to deadenylate tubulin mRNA to initiate its subsequent degradation.

experiments suggest that CNOT11 may contact the mRNA-binding 40S subunit.[60] SCAPER therefore acts as a molecular bridge that effectively communicates a nascent chain recognition event at the exit tunnel on the ribosome 60S subunit to a deadenylase activity that may reside near the mRNA channel of the 40S subunit. The flexibility of both the CCR4-NOT complex and the downstream mRNA would then allow access to the 3′ end for deadenylation. Thus, CCR4-NOT can be deployed to selective ribosomes on the basis of the nascent polypeptides they display, a mode of action qualitatively different from direct binding to either ribosomes or sequence-specific mRNA-binding adaptors.[23,49,61]

Our mechanistic dissection of nascent tubulin-dependent recruitment of the CCR4-NOT complex provides a framework for understanding analogous regulatory processes for other proteins. For example, the stability of mRNAs coding for at least some ribosomal proteins is coupled to the availability of chaperones dedicated to these proteins in budding yeast.[26] Degradation of these mRNAs in the absence of chaperones is thought to be co-translational, but neither the basis of nascent chain recognition nor the mechanism of putative CCR4-NOT recruitment are understood. The methods and principles from the tubulin autoregulation pathway provide a roadmap to now dissect the analogous processes for ribosomal proteins and others.

Mutations in TTC5 and the N-terminal recognition motif in a tubulin gene have previously been linked to neurodevelopmental defects,[62,63] hinting at physiologic role(s) for autoregulation. However, potential added roles for TTC5 in regulation of transcription and the actin cytoskeleton,[64,65] and putative consequences for tubulin structure complicated this interpretation. Our assignment of SCAPER to the autoregulation pathway and characterization of autoregulation-disrupting mutants now substantially strengthen the link between autoregulation and human physiology. SCAPER disease variants lead to ciliopathy-related syndromes comprising intellectual disability, retinitis pigmentosa, male infertility, and other symptoms.[42–44] These phenotypes overlap partially with both TTC5-linked disease and tubulinopathies, providing insights into the tissues and biological processes most reliant on tubulin autoregulation. Interestingly, the nervous system is exquisitely sensitive to mutations that cause DNA damage or chromosome segregation defects, possibly due to the rapid proliferation of neuronal progenitor cells required during brain development.[58,59,66] Thus, the complex phenotypes seen in humans mutant for SCAPER may be due to a combination of defective ciliogenesis, chromosome segregation, and some of the many other tubulin-related processes.

How the tubulin autoregulation pathway is controlled in response to changes in MT or free tubulin levels remains enigmatic. Previous work has shown that the access of TTC5 to tubulin RNCs is regulated by a yet-unidentified sequestration factor that releases TTC5 when tubulin autoregulation is triggered.[17] Furthermore, the previously identified cyclin A binding site of SCAPER,[40] and its putative MT binding activity[44] suggest potential mechanisms of regulation. Indeed, tubulin mRNA levels have been observed to change through the cell cycle as might be needed to accommodate different roles of the MT network.[67,68] More generally, multi-component pathways provide ample scope for temporal and context-dependent regulation.[69,70] In tubulin autoregulation, the specificity factor TTC5, the adaptor SCAPER, the substrate-recruitment subunit CNOT11, and the deadenylase complex CCR4-NOT could all be fine-tuned to ensure accurate tubulin levels in a cell-type-specific manner.

Limitations of the study

Our study provides strong evidence that TTC5, which recognizes tubulin-synthesizing ribosomes, subsequently recruits SCAPER that, in turn, recruits the CCR4-NOT deadenylation complex. In our structure of the ribosome-TTC5-SCAPER complex, only the C-terminal globular domain of SCAPER was resolved, so we cannot visualize how the CCR4-NOT complex engages

CellPress
OPEN ACCESS

with translating ribosomes. Similarly, the AF2 prediction of CNOT11 bound to SCAPER has been validated by mutagenesis, but not by direct structural methods. The mechanism by which tubulin autoregulation is activated by elevated free tubulin levels has not been addressed by our work. Although the requirement for TTC5, SCAPER, and CCR4-NOT has been established, their sufficiency for mRNA degradation has not been established by *in vitro* reconstitution. How defects in tubulin autoregulation contribute to the phenotypes of pathological SCAPER and TTC5 mutations on an organismal level will require further investigation.

STAR★METHODS

Detailed methods are provided in the online version of this paper and include the following:

- KEY RESOURCES TABLE
- RESOURCE AVAILABILITY
  - Lead contact
  - Materials availability
  - Data and code availability
- EXPERIMENTAL MODEL AND STUDY PARTICIPANT DETAILS
  - Cell lines
- METHOD DETAILS
  - Plasmids and reagents
  - Cell culture procedures
  - Live cell imaging and data analysis
  - Western blot analysis
  - mRNA quantification by RT-qPCR
  - Poly(A) tail-length assay
  - Pulse labelling of protein synthesis
  - Biotin proximity labelling procedure
  - Quantitative proteomics procedures
  - Recombinant protein purification
  - Pull-down assays
  - In vitro transcription and translation
  - Structural analysis of TTC5-SCAPER-ribosomes
  - Structural modelling
- QUANTIFICATION AND STATISTICAL ANALYSIS
  - Quantification
  - Statistical analysis
  - Reproducibility

SUPPLEMENTAL INFORMATION

Supplemental information can be found online at https://doi.org/10.1016/j.molcel.2023.05.020.

ACKNOWLEDGMENTS

We are grateful to the electron microscopy facility staff at the MRC Laboratory of Molecular Biology for access and support for sample preparation and data collection; Z. Lin for reagents and useful discussions during the early stages of this project; J. Grimmett and T. Darling for maintenance of the LMB scientific computing infrastructure; F. Begum and M. Skehel for mass spectrometry; C. Lau and S. Chaaban for help with a local implementation of AF2; V. Chandrasekaran and J.P. O'Donnell for comments on the manuscript; the Photonic Bioimaging Center of the University of Geneva for help with microscopy. This work was supported by the Medical Research Council, as part of United Kingdom Research and Innovation (MC_U105192715 to L.A.P. and MC_UP_A022_1007 to R.S.H.). M.H. received funding from EMBO (ALTF 116-2020) and the European Union's Horizon 2020 research and innovation programme under the Marie Sklodowska-Curie grant agreement no 101029853. E.A. was funded by a postdoctoral fellowship of the Deutsche Forschungsgemeinschaft (DFG, German Research Foundation, project number 429892960). I.G. is supported by the Swiss National Science Foundation Eccellenza Fellowship (PCEFP3_194312) and is the Dale F. Frey Breakthrough Scientist of the Damon Runyon Cancer Research Foundation (DRG:2279-16). We acknowledge Diamond Light Source for access to eBIC (proposal BI23268) funded by the Wellcome Trust, Medical Research Council, and Biotechnology and Biological Sciences Research Council.

AUTHOR CONTRIBUTIONS

M.H. discovered the role for SCAPER in tubulin autoregulation and performed most experiments. E.A. performed structural analysis of the TTC5-SCAPER-ribosome complex. S.-Y.P.-C. analyzed mass spectrometry samples. I.G. and E.V. generated and validated HeLa cell lines. I.G. designed and performed phenotypic analysis of mitosis. R.S.H., L.A.P., and I.G. supervised different parts of the project. R.S.H. and M.H. conceived the project, oversaw its implementation, and wrote the manuscript. All authors contributed to manuscript editing.

DECLARATION OF INTERESTS

L.A.P. is an inventor on a patent filed by the Medical Research Council for all-gold EM supports, licensed to Quantifoil under the trademark UltrAuFoil. L.A.P. and R.S.H. are on the advisory board for *Molecular Cell*.

Received: December 16, 2022
Revised: March 28, 2023
Accepted: May 15, 2023
Published: June 8, 2023

REFERENCES

1. Goodson, H.V., and Jonasson, E.M. (2018). Microtubules and microtubule-associated proteins. Cold Spring Harb. Perspect. Biol. *10*, a022608. https://doi.org/10.1101/cshperspect.a022608.
2. Gudimchuk, N.B., and McIntosh, J.R. (2021). Regulation of microtubule dynamics, mechanics and function through the growing tip. Nat. Rev. Mol. Cell Biol. *22*, 777–795. https://doi.org/10.1038/s41580-021-00399-x.
3. Bodakuntla, S., Jijumon, A.S., Villablanca, C., Gonzalez-Billault, C., and Janke, C. (2019). Microtubule-associated proteins: structuring the cytoskeleton. Trends Cell Biol. *29*, 804–819. https://doi.org/10.1016/j.tcb.2019.07.004.
4. Janke, C., and Magiera, M.M. (2020). The tubulin code and its role in controlling microtubule properties and functions. Nat. Rev. Mol. Cell Biol. *21*, 307–326. https://doi.org/10.1038/s41580-020-0214-3.
5. Nieuwenhuis, J., and Brummelkamp, T.R. (2019). The tubulin detyrosination cycle: function and enzymes. Trends Cell Biol. *29*, 80–92. https://doi.org/10.1016/j.tcb.2018.08.003.
6. Nieuwenhuis, J., Adamopoulos, A., Bleijerveld, O.B., Mazouzi, A., Stickel, E., Celie, P., Altelaar, M., Knipscheer, P., Perrakis, A., Blomen, V.A., et al. (2017). Vasohibins encode tubulin detyrosinating activity. Science *358*, 1453–1456. https://doi.org/10.1126/science.aao5676.
7. Landskron, L., Bak, J., Adamopoulos, A., Kaplani, K., Moraiti, M., Hengel, L.G. van den, Song, J.-Y., Bleijerveld, O.B., Nieuwenhuis, J., Heidebrecht, T., et al. (2022). Posttranslational modification of microtubules by the MATCAP detyrosinase. Science *376*, eabn6020. https://doi.org/10.1126/science.abn6020.
8. Jijumon, A.S., Bodakuntla, S., Genova, M., Bangera, M., Sackett, V., Besse, L., Maksut, F., Henriot, V., Magiera, M.M., Sirajuddin, M., et al. (2022). Lysate-based pipeline to characterize microtubule-associated

proteins uncovers unique microtubule behaviours. Nat. Cell Biol. *24*, 253–267. https://doi.org/10.1038/s41556-021-00825-4.

9. Dominguez-Brauer, C., Thu, K.L., Mason, J.M., Blaser, H., Bray, M.R., and Mak, T.W. (2015). Targeting mitosis in cancer: emerging strategies. Mol. Cell *60*, 524–536. https://doi.org/10.1016/j.molcel.2015.11.006.
10. Dumontet, C., and Jordan, M.A. (2010). Microtubule-binding agents: a dynamic field of cancer therapeutics. Nat. Rev. Drug Discov. *9*, 790–803. https://doi.org/10.1038/nrd3253.
11. Matamoros, A.J., and Baas, P.W. (2016). Microtubules in health and degenerative disease of the nervous system. Brain Res. Bull. *126*, 217–225. https://doi.org/10.1016/j.brainresbull.2016.06.016.
12. Lasser, M., Tiber, J., and Lowery, L.A. (2018). The role of the microtubule cytoskeleton in neurodevelopmental disorders. Front. Cell. Neurosci. *12*, 165. https://doi.org/10.3389/fncel.2018.00165.
13. Mitchison, T., and Kirschner, M. (1984). Dynamic instability of microtubule growth. Nature *312*, 237–242. https://doi.org/10.1038/312237a0.
14. Cleveland, D.W., Lopata, M.A., Sherline, P., and Kirschner, M.W. (1981). Unpolymerized tubulin modulates the level of tubulin mRNAs. Cell *25*, 537–546. https://doi.org/10.1016/0092-8674(81)90072-6.
15. Yen, T.J., Machlin, P.S., and Cleveland, D.W. (1988). Autoregulated instability of beta-tubulin mRNAs by recognition of the nascent amino terminus of beta-tubulin. Nature *334*, 580–585. https://doi.org/10.1038/334580a0.
16. Gasic, I., and Mitchison, T.J. (2019). Autoregulation and repair in microtubule homeostasis. Curr. Opin. Cell Biol. *56*, 80–87. https://doi.org/10.1016/j.ceb.2018.10.003.
17. Lin, Z., Gasic, I., Chandrasekaran, V., Peters, N., Shao, S., Mitchison, T.J., and Hegde, R.S. (2020). TTC5 mediates autoregulation of tubulin via mRNA degradation. Science *367*, 100–104. https://doi.org/10.1126/science.aaz4352.
18. Yen, T.J., Gay, D.A., Pachter, J.S., and Cleveland, D.W. (1988). Autoregulated changes in stability of polyribosome-bound beta-tubulin mRNAs are specified by the first 13 translated nucleotides. Mol. Cell. Biol. *8*, 1224–1235. https://doi.org/10.1128/mcb.8.3.1224-1235.1988.
19. Petry, S. (2016). Mechanisms of mitotic spindle assembly. Annu. Rev. Biochem. *85*, 659–683. https://doi.org/10.1146/annurev-biochem-060815-014528.
20. Vicente, J.J., and Wordeman, L. (2019). The quantification and regulation of microtubule dynamics in the mitotic spindle. Curr. Opin. Cell Biol. *60*, 36–43. https://doi.org/10.1016/j.ceb.2019.03.017.
21. Chen, C.-Y.A., and Shyu, A.-B. (1995). AU-rich elements: characterization and importance in mRNA degradation. Trends Biochem. Sci. *20*, 465–470. https://doi.org/10.1016/s0968-0004(00)89102-1.
22. Bartel, D.P. (2018). Metazoan microRNAs. Cell *173*, 20–51. https://doi.org/10.1016/j.cell.2018.03.006.
23. Passmore, L.A., and Coller, J. (2022). Roles of mRNA poly(A) tails in regulation of eukaryotic gene expression. Nat. Rev. Mol. Cell Biol. *23*, 93–106. https://doi.org/10.1038/s41580-021-00417-y.
24. Hollien, J., and Weissman, J.S. (2006). Decay of endoplasmic reticulum-localized mRNAs during the unfolded protein response. Science *313*, 104–107. https://doi.org/10.1126/science.1129631.
25. Karamyshev, A.L., Patrick, A.E., Karamysheva, Z.N., Griesemer, D.S., Hudson, H., Tjon-Kon-Sang, S., Nilsson, I., Otto, H., Liu, Q., Rospert, S., et al. (2014). Inefficient SRP interaction with a nascent chain triggers a mRNA quality control pathway. Cell *156*, 146–157. https://doi.org/10.1016/j.cell.2013.12.017.
26. Pillet, B., Méndez-Godoy, A., Murat, G., Favre, S., Stumpe, M., Falquet, L., and Kressler, D. (2022). Dedicated chaperones coordinate co-translational regulation of ribosomal protein production with ribosome assembly to preserve proteostasis. eLife *11*, e74255. https://doi.org/10.7554/eLife.74255.
27. Marino, N.D., Zhang, J.Y., Borges, A.L., Sousa, A.A., Leon, L.M., Rauch, B.J., Walton, R.T., Berry, J.D., Joung, J.K., Kleinstiver, B.P., et al. (2018). Discovery of widespread type I and type V CRISPR-Cas inhibitors. Science *362*, 240–242. https://doi.org/10.1126/science.aau5174.
28. Marino, N.D., Talaie, A., Carion, H., Zhang, Y., Silas, S., Li, Y., and Bondy-Denomy, J. (2022). Translation-dependent downregulation of Cas12a mRNA by an anti-CRISPR protein. Preprint at bioRxiv. https://doi.org/10.1101/2022.11.29.518452.
29. Kilchert, C., and Spang, A. (2011). Cotranslational transport of ABP140 mRNA to the distal pole of S. cerevisiae. EMBO J. *30*, 3567–3580. https://doi.org/10.1038/emboj.2011.247.
30. Moissoglu, K., Stueland, M., Gasparski, A.N., Wang, T., Jenkins, L.M., Hastings, M.L., and Mili, S. (2020). RNA localization and co-translational interactions control RAB13 GTPase function and cell migration. EMBO J. *39*, e104958. https://doi.org/10.15252/embj.2020104958.
31. Safieddine, A., Coleno, E., Salloum, S., Imbert, A., Traboulsi, A.M., Kwon, O.S., Lionneton, F., Georget, V., Robert, M.-C., Gostan, T., et al. (2021). A choreography of centrosomal mRNAs reveals a conserved localization mechanism involving active polysome transport. Nat. Commun. *12*, 1352. https://doi.org/10.1038/s41467-021-21585-7.
32. Cassella, L., and Ephrussi, A. (2022). Subcellular spatial transcriptomics identifies three mechanistically different classes of localizing RNAs. Nat. Commun. *13*, 6355. https://doi.org/10.1038/s41467-022-34004-2.
33. Williams, N.K., and Dichtl, B. (2018). Co-translational control of protein complex formation: a fundamental pathway of cellular organization? Biochem. Soc. Trans. *46*, 197–206. https://doi.org/10.1042/BST20170451.
34. Shiber, A., Döring, K., Friedrich, U., Klann, K., Merker, D., Zedan, M., Tippmann, F., Kramer, G., and Bukau, B. (2018). Cotranslational assembly of protein complexes in eukaryotes revealed by ribosome profiling. Nature *561*, 268–272. https://doi.org/10.1038/s41586-018-0462-y.
35. Muroyama, A., and Lechler, T. (2017). Microtubule organization, dynamics and functions in differentiated cells. Development *144*, 3012–3021. https://doi.org/10.1242/dev.153171.
36. Branon, T.C., Bosch, J.A., Sanchez, A.D., Udeshi, N.D., Svinkina, T., Carr, S.A., Feldman, J.L., Perrimon, N., and Ting, A.Y. (2018). Efficient proximity labeling in living cells and organisms with TurboID. Nat. Biotechnol. *36*, 880–887. https://doi.org/10.1038/nbt.4201.
37. Thul, P.J., Åkesson, L., Wiking, M., Mahdessian, D., Geladaki, A., Ait Blal, H.A., Alm, T., Asplund, A., Björk, L., Breckels, L.M., et al. (2017). A subcellular map of the human proteome. Science *356*, eaal3321. https://doi.org/10.1126/science.aal3321.
38. Jumper, J., Evans, R., Pritzel, A., Green, T., Figurnov, M., Ronneberger, O., Tunyasuvunakool, K., Bates, R., Žídek, A., Potapenko, A., et al. (2021). Highly accurate protein structure prediction with AlphaFold. Nature *596*, 583–589. https://doi.org/10.1038/s41586-021-03819-2.
39. Evans, R., O'Neill, M., Pritzel, A., Antropova, N., Senior, A., Green, T., Žídek, A., Bates, R., Blackwell, S., Yim, J., et al. (2022). Protein complex prediction with AlphaFold-Multimer. Preprint at bioRxiv. https://doi.org/10.1101/2021.10.04.463034.
40. Tsang, W.Y., Wang, L., Chen, Z., Sánchez, I., and Dynlacht, B.D. (2007). SCAPER, a novel cyclin A–interacting protein that regulates cell cycle progression. J. Cell Biol. *178*, 621–633. https://doi.org/10.1083/jcb.200701166.
41. Tatour, Y., Sanchez-Navarro, I., Chervinsky, E., Hakonarson, H., Gawi, H., Tahsin-Swafiri, S., Leibu, R., Lopez-Molina, M.I., Fernandez-Sanz, G., Ayuso, C., et al. (2017). Mutations in SCAPER cause autosomal recessive retinitis pigmentosa with intellectual disability. J. Med. Genet. *54*, 698–704. https://doi.org/10.1136/jmedgenet-2017-104632.
42. Fasham, J., Arno, G., Lin, S., Xu, M., Carss, K.J., Hull, S., Lane, A., Robson, A.G., Wenger, O., Self, J.E., et al. (2019). Delineating the expanding phenotype associated with SCAPER gene mutation. Am. J. Med. Genet. A *179*, 1665–1671. https://doi.org/10.1002/ajmg.a.61202.
43. Wormser, O., Gradstein, L., Yogev, Y., Perez, Y., Kadir, R., Goliand, I., Sadka, Y., El Riati, S.E., Flusser, H., Nachmias, D., et al. (2019). SCAPER localizes to primary cilia and its mutation affects cilia length, causing Bardet-Biedl syndrome. Eur. J. Hum. Genet. *27*, 928–940. https://doi.org/10.1038/s41431-019-0347-z.

CellPress
OPEN ACCESS

44. Wormser, O., Levy, Y., Bakhrat, A., Bonaccorsi, S., Graziadio, L., Gatti, M., AbuMadighem, A., McKenney, R.J., Okada, K., El Riati, S.E., et al. (2021). Absence of SCAPER causes male infertility in humans and Drosophila by modulating microtubule dynamics during meiosis. J. Med. Genet. *58*, 254–263. https://doi.org/10.1136/jmedgenet-2020-106946.

45. Fujii, K., Susanto, T.T., Saurabh, S., and Barna, M. (2018). Decoding the function of expansion segments in ribosomes. Mol. Cell *72*, 1013–1020.e6. https://doi.org/10.1016/j.molcel.2018.11.023.

46. Knorr, A.G., Schmidt, C., Tesina, P., Berninghausen, O., Becker, T., Beatrix, B., and Beckmann, R. (2019). Ribosome–NatA architecture reveals that rRNA expansion segments coordinate N-terminal acetylation. Nat. Struct. Mol. Biol. *26*, 35–39. https://doi.org/10.1038/s41594-018-0165-y.

47. Wild, K., Aleksić, M., Lapouge, K., Juaire, K.D., Flemming, D., Pfeffer, S., and Sinning, I. (2020). MetAP-like Ebp1 occupies the human ribosomal tunnel exit and recruits flexible rRNA expansion segments. Nat. Commun. *11*, 776. https://doi.org/10.1038/s41467-020-14603-7.

48. Decker, C.J., and Parker, R. (1993). A turnover pathway for both stable and unstable mRNAs in yeast: evidence for a requirement for deadenylation. Genes Dev. *7*, 1632–1643. https://doi.org/10.1101/gad.7.8.1632.

49. Raisch, T., and Valkov, E. (2022). Regulation of the multisubunit CCR4-NOT deadenylase in the initiation of mRNA degradation. Curr. Opin. Struct. Biol. *77*, 102460. https://doi.org/10.1016/j.sbi.2022.102460.

50. Kusov, Y.Yu., Shatirishvili, G., Dzagurov, G., and Gauss-Müller, V. (2001). A new G-tailing method for the determination of the poly(A) tail length applied to hepatitis A virus RNA. Nucleic Acids Res. *29*. E57–E57. https://doi.org/10.1093/nar/29.12.e57.

51. Enwerem, I.I.I., Elrod, N.D., Chang, C.-T., Lin, A., Ji, P., Bohn, J.A., Levdansky, Y., Wagner, E.J., Valkov, E., and Goldstrohm, A.C. (2021). Human Pumilio proteins directly bind the CCR4-NOT deadenylase complex to regulate the transcriptome. Rna *27*, 445–464. https://doi.org/10.1261/rna.078436.120.

52. Mauxion, F., Prève, B., and Séraphin, B. (2013). C2ORF29/CNOT11 and CNOT10 form a new module of the CCR4-NOT complex. RNA Biol. *10*, 267–276. https://doi.org/10.4161/rna.23065.

53. Bohn, J.A., Van Etten, J.L.V., Schagat, T.L., Bowman, B.M., McEachin, R.C., Freddolino, P.L., and Goldstrohm, A.C. (2018). Identification of diverse target RNAs that are functionally regulated by human Pumilio proteins. Nucleic Acids Res. *46*, 362–386. https://doi.org/10.1093/nar/gkx1120.

54. Gillen, S.L., Giacomelli, C., Hodge, K., Zanivan, S., Bushell, M., and Wilczynska, A. (2021). Differential regulation of mRNA fate by the human Ccr4-Not complex is driven by coding sequence composition and mRNA localization. Genome Biol. *22*, 284. https://doi.org/10.1186/s13059-021-02494-w.

55. Mauxion, F., Basquin, J., Ozgur, S., Rame, M., Albrecht, J., Schäfer, I., Séraphin, B., and Conti, E. (2023). The human CNOT1-CNOT10-CNOT11 complex forms a structural platform for protein-protein interactions. Cell Rep. *42*, 111902. https://doi.org/10.1016/j.celrep.2022.111902.

56. Funk, L., Su, K.C., Ly, J., Feldman, D., Singh, A., Moodie, B., Blainey, P.C., and Cheeseman, I.M. (2022). The phenotypic landscape of essential human genes. Cell *185*, 4634–4653.e22. https://doi.org/10.1016/j.cell.2022.10.017.

57. Ben-David, U., and Amon, A. (2020). Context is everything: aneuploidy in cancer. Nat. Rev. Genet. *21*, 44–62. https://doi.org/10.1038/s41576-019-0171-x.

58. Saade, M., Blanco-Ameijeiras, J., Gonzalez-Gobartt, E., and Martí, E. (2018). A centrosomal view of CNS growth. Development *145*, dev170613. https://doi.org/10.1242/dev.170613.

59. González-Martínez, J., Cwetsch, A.W., Martínez-Alonso, D., López-Sainz, L.R., Almagro, J., Melati, A., Gómez, J., Pérez-Martínez, M., Megías, D., Boskovic, J., et al. (2021). Deficient adaptation to centrosome duplication defects in neural progenitors causes microcephaly and subcortical heterotopias. JCI Insight *6*, e146364. https://doi.org/10.1172/jci.insight.146364.

60. Absmeier, E., Chandrasekaran, V., O'Reilly, F.J., Stowell, J.A., Rappsilber, J., and Passmore, L.A. (2022). Specific recognition and ubiquitination of slow-moving ribosomes by human CCR4-NOT. Preprint at bioRxiv. https://doi.org/10.1101/2022.07.24.501325.

61. Buschauer, R., Matsuo, Y., Sugiyama, T., Chen, Y.-H., Alhusaini, N., Sweet, T., Ikeuchi, K., Cheng, J., Matsuki, Y., Nobuta, R., et al. (2020). The Ccr4-Not complex monitors the translating ribosome for codon optimality. Science *368*, eaay6912. https://doi.org/10.1126/science.aay6912.

62. Musante, L., Faletra, F., Meier, K., Tomoum, H., Najarzadeh Torbati, P., Blair, E., North, S., Gärtner, J., Diegmann, S., Beiraghi Toosi, M., et al. (2022). TTC5 syndrome: clinical and molecular spectrum of a severe and recognizable condition. Am. J. Med. Genet. A *188*, 2652–2665. https://doi.org/10.1002/ajmg.a.62852.

63. Gardner, J.F., Cushion, T.D., Niotakis, G., Olson, H.E., Grant, P.E., Scott, R.H., Stoodley, N., Cohen, J.S., Naidu, S., Attie-Bitach, T., et al. (2018). Clinical and functional characterization of the recurrent TUBA1A p.(Arg2His). Mutation. Brain Sci. *8*, 145. https://doi.org/10.3390/brainsci8080145.

64. Demonacos, C., Krstic-Demonacos, M., and La Thangue, N.B.L. (2001). A TPR motif cofactor contributes to p300 activity in the p53 response. Mol. Cell *8*, 71–84. https://doi.org/10.1016/s1097-2765(01)00277-5.

65. Hu, X., and Mullins, R.D. (2019). LC3 and STRAP regulate actin filament assembly by JMY during autophagosome formation. J. Cell Biol. *218*, 251–266. https://doi.org/10.1083/jcb.201802157.

66. Caldecott, K.W. (2022). DNA single-strand break repair and human genetic disease. Trends Cell Biol. *32*, 733–745. https://doi.org/10.1016/j.tcb.2022.04.010.

67. Krenning, L., Sonneveld, S., and Tanenbaum, M.E. (2022). Time-resolved single-cell sequencing identifies multiple waves of mRNA decay during the mitosis-to-G1 phase transition. eLife *11*, e71356. https://doi.org/10.7554/eLife.71356.

68. Schedl, T., Burland, T.G., Gull, K., and Dove, W.F. (1984). Cell cycle regulation of tubulin RNA level, tubulin protein synthesis, and assembly of microtubules in Physarum. J. Cell Biol. *99*, 155–165. https://doi.org/10.1083/jcb.99.1.155.

69. Varshavsky, A. (2012). The ubiquitin system, an immense realm. Annu. Rev. Biochem. *81*, 167–176. https://doi.org/10.1146/annurev-biochem-051910-094049.

70. Vihervaara, A., Duarte, F.M., and Lis, J.T. (2018). Molecular mechanisms driving transcriptional stress responses. Nat. Rev. Genet. *19*, 385–397. https://doi.org/10.1038/s41576-018-0001-6.

71. O'Donnell, J.P., Phillips, B.P., Yagita, Y., Juszkiewicz, S., Wagner, A., Malinverni, D., Keenan, R.J., Miller, E.A., and Hegde, R.S. (2020). The architecture of EMC reveals a path for membrane protein insertion. eLife *9*, e57887. https://doi.org/10.7554/eLife.57887.

72. Brinkman, E.K., Chen, T., Amendola, M., and van Steensel, B. (2014). Easy quantitative assessment of genome editing by sequence trace decomposition. Nucleic Acids Res. *42*, e168. https://doi.org/10.1093/nar/gku936.

73. Schindelin, J., Arganda-Carreras, I., Frise, E., Kaynig, V., Longair, M., Pietzsch, T., Preibisch, S., Rueden, C., Saalfeld, S., Schmid, B., et al. (2012). Fiji: an open-source platform for biological-image analysis. Nat. Methods *9*, 676–682. https://doi.org/10.1038/nmeth.2019.

74. Cox, J., and Mann, M. (2008). MaxQuant enables high peptide identification rates, individualized p.p.b.-range mass accuracies and proteome-wide protein quantification. Nat. Biotechnol. *26*, 1367–1372. https://doi.org/10.1038/nbt.1511.

75. Tyanova, S., Temu, T., Sinitcyn, P., Carlson, A., Hein, M.Y., Geiger, T., Mann, M., and Cox, J. (2016). The Perseus computational platform for comprehensive analysis of (prote)omics data. Nat. Methods *13*, 731–740. https://doi.org/10.1038/nmeth.3901.

76. Kimanius, D., Dong, L., Sharov, G., Nakane, T., and Scheres, S.H.W. (2021). New tools for automated cryo-EM single-particle analysis in RELION-4.0. Biochem. J. *478*, 4169–4185. https://doi.org/10.1042/BCJ20210708.

77. Emsley, P., Lohkamp, B., Scott, W.G., and Cowtan, K. (2010). Features and development of coot. Acta Crystallogr. D Biol. Crystallogr. *66*, 486–501. https://doi.org/10.1107/S0907444910007493.

78. Adams, P.D., Afonine, P.V., Bunkóczi, G., Chen, V.B., Davis, I.W., Echols, N., Headd, J.J., Hung, L.-W., Kapral, G.J., Grosse-Kunstleve, R.W., et al. (2010). Phenix: a comprehensive Python-based system for macromolecular structure solution. Acta Crystallogr. D Biol. Crystallogr. *66*, 213–221. https://doi.org/10.1107/S0907444909052925.

79. Pettersen, E.F., Goddard, T.D., Huang, C.C., Couch, G.S., Greenblatt, D.M., Meng, E.C., and Ferrin, T.E. (2004). UCSF Chimera—A visualization system for exploratory research and analysis. J. Comput. Chem. *25*, 1605–1612. https://doi.org/10.1002/jcc.20084.

80. Goddard, T.D., Huang, C.C., Meng, E.C., Pettersen, E.F., Couch, G.S., Morris, J.H., and Ferrin, T.E. (2018). UCSF ChimeraX: meeting modern challenges in visualization and analysis. Protein Sci. *27*, 14–25. https://doi.org/10.1002/pro.3235.

81. Mirdita, M., Schütze, K., Moriwaki, Y., Heo, L., Ovchinnikov, S., and Steinegger, M. (2022). ColabFold: making protein folding accessible to all. Nat. Methods *19*, 679–682. https://doi.org/10.1038/s41592-022-01488-1.

82. Ashkenazy, H., Abadi, S., Martz, E., Chay, O., Mayrose, I., Pupko, T., and Ben-Tal, N. (2016). ConSurf 2016: an improved methodology to estimate and visualize evolutionary conservation in macromolecules. Nucleic Acids Res. *44*, W344–W350. https://doi.org/10.1093/nar/gkw408.

83. Sievers, F., Wilm, A., Dineen, D., Gibson, T.J., Karplus, K., Li, W., Lopez, R., McWilliam, H., Remmert, M., Söding, J., et al. (2011). Fast, scalable generation of high-quality protein multiple sequence alignments using Clustal Omega. Mol. Syst. Biol. *7*, 539. https://doi.org/10.1038/msb.2011.75.

84. Waterhouse, A.M., Procter, J.B., Martin, D.M.A., Clamp, M., and Barton, G.J. (2009). Jalview, version 2—a multiple sequence alignment editor and analysis workbench. Bioinformatics *25*, 1189–1191. https://doi.org/10.1093/bioinformatics/btp033.

85. Sharma, A., Mariappan, M., Appathurai, S., and Hegde, R.S. (2010). In vitro dissection of protein translocation into the mammalian endoplasmic reticulum. Methods Mol. Biol. *619*, 339–363. https://doi.org/10.1007/978-1-60327-412-8_20.

86. Ran, F.A., Hsu, P.D., Wright, J., Agarwala, V., Scott, D.A., and Zhang, F. (2013). Genome engineering using the CRISPR-Cas9 system. Nat. Protoc. *8*, 2281–2308. https://doi.org/10.1038/nprot.2013.143.

87. Livak, K.J., and Schmittgen, T.D. (2001). Analysis of relative gene expression data using real-time quantitative PCR and the 2-ΔΔC T method. Methods *25*, 402–408. https://doi.org/10.1006/meth.2001.1262.

88. Cho, K.F., Branon, T.C., Udeshi, N.D., Myers, S.A., Carr, S.A., and Ting, A.Y. (2020). Proximity labeling in mammalian cells with TurboID and split-TurboID. Nat. Protoc. *15*, 3971–3999. https://doi.org/10.1038/s41596-020-0399-0.

89. Feng, Q., and Shao, S. (2018). In vitro reconstitution of translational arrest pathways. Methods *137*, 20–36. https://doi.org/10.1016/j.ymeth.2017.12.018.

90. Varadi, M., Anyango, S., Deshpande, M., Nair, S., Natassia, C., Yordanova, G., Yuan, D., Stroe, O., Wood, G., Laydon, A., et al. (2022). AlphaFold Protein Structure Database: massively expanding the structural coverage of protein-sequence space with high-accuracy models. Nucleic Acids Res. *50*, D439–D444. https://doi.org/10.1093/nar/gkab1061.

91. Mirdita, M., Steinegger, M., and Söding, J. (2019). MMseqs2 desktop and local web server app for fast, interactive sequence searches. Bioinformatics *35*, 2856–2858. https://doi.org/10.1093/bioinformatics/bty1057.

CellPress
OPEN ACCESS

STAR★METHODS

KEY RESOURCES TABLE

REAGENT or RESOURCE	SOURCE	IDENTIFIER
**Antibodies**		
β-actin HRP-conjugated	Sigma-Aldrich	Cat#A3854; RRID:AB_262011
GAPDH	Cell Signaling Technology	Cat#2118, RRID:AB_561053
RPS24	abcam	Cat#ab196652, RRID:AB_2714188
RPL8	abcam	Cat#ab169538, RRID:AB_2714187
TTC5	Epigentek	Cat#A66330
SCAPER	Thermo Fisher	Cat#PA5-69015, RRID:AB_2689457
SCAPER	Thermo Fisher	Cat#PA5-61195, RRID:AB_2646987
CNOT1	Proteintech	Cat#01397000014276-I-AP, RRID:AB_10888627
CNOT3	Abnova	Cat#H00004849-M01, RRID:AB_489915
CNOT4	abcam	Cat#ab214937
CNOT6	abcam	Cat#ab221151, RRID:AB_2861188
CNOT6L	Fisher Scientific	Cat#PA5114256, RRID:AB_2884770
CNOT7	abcam	Cat#ab195587, RRID:AB_2801659
CNOT8	Proteintech	Cat#10752-1-AP, RRID:AB_2082470
CNOT9	Proteintech	Cat#PT22503-1-AP, RRID:AB_11232413
CNOT11	Santa Cruz	Cat#sc-377068
PAN2	abcam	Cat#ab241505
FLAG-tag	Sigma-Aldrich	Cat#F3165, RRID:AB_259529
FLAG-tag, HRP-coupled	Sigma-Aldrich	Cat#A8592, RRID:AB_439702
StrepII-tag	abcam	Cat#ab76949, RRID:AB_1524455
HA-tag (custom made)	O'Donnell et al.[71]	N/A
HRP-conjugated secondary antibody (mouse)	Thermo Fisher	Cat#31430, RRID:AB_228307
HRP-conjugated secondary antibody (rabbit)	Thermo Fisher	Cat#31460, RRID:AB_228341
HRP-conjugated goat polyclonal anti-rabbit IgG (H + L)	Jackson Immuno Research Labs	Cat#111-035-003; RRID:AB_2313567
HRP-conjugated goat polyclonal anti-mouse IgG (H + L)	Jackson Immuno Research Labs	Cat#115-035-003; RRID:AB_10015289
**Bacterial and virus strains**		
E. coli BL21(DE3)	Thermo Fisher	Cat#EC0114
**Chemicals, peptides, and recombinant proteins**		
Blasticidin S	Santa Cruz Biotechnology	Cat#sc-204655; CAS: 3513-03-9
Hygromycin B	Sigma-Aldrich	Cat#400051; CAS: 31282-04-9
PEI MAX - Transfection Grade	Polysciences	Cat#24765; CAS: 49553-93-7
Doxycycline	Sigma-Aldrich	Cat#D9891; CAS: 24390-14-5
Puromycin	Sigma-Aldrich	Cat#P8833, CAS: 58-58-2
Digitonin, High Purity	Millipore	Cat#300410; CAS: 11024-24-1
cOmplete, EDTA-free Protease Inhibitor Cocktail	Roche	Cat#11873580001
3xFLAG Peptide	Sigma-Aldrich	Cat#F4799
Recombinant RNasin Ribonuclease Inhibitor	Promega	Cat#N2518
SP6 RNA Polymerase	New England Biolabs	Cat#M0207
EasyTag L-[^{35}S]-Methionine	Perkin Elmer	Cat#NEG709A005MC
SYPRO Ruby Protein Gel Stain	Invitrogen	Cat#S12000
Ponceau S solution	Sigma-Aldrich	Cat#P-7170; CAS: 6226-79-5

(*Continued on next page*)

Continued		
REAGENT or RESOURCE	SOURCE	IDENTIFIER
Lipofectamine 3000	Invitrogen	Cat#L3000001
TransIT-293 Transfection Reagent	Mirus	Cat#MIR 2700
Alt-R S.p. Cas9 Nuclease V3	IDT	Cat#1081058
Lipofectamine RNAiMAX	Invitrogen	Cat#13778150
Sir-DNA	Cytoskeleton	Cat#CY-SC007
Pierce ECL Western Blotting Substrate	Thermo Fisher	Cat#32209
SuperSignal West Pico PLUS Chemiluminescent Substrate	Thermo Fisher	Cat#34080
SYBR Safe DNA gel stain	Thermo Fisher	Cat#S33102
Colchicine	Sigma-Aldrich	Cat#PRH1764
Combretastatin A4	Selleckchem	Cat#S7783
Nocodazole	Sigma-Aldrich	Cat#SML1665
Actinomycin D	Sigma-Aldrich	Cat#A1410
TCEP (Tris(2-carboxyethyl)phosphine hydrochloride)	Sigma-Aldrich	Cat#C4706, CAS: 51805-45-9
Biotin	APExBIO	Cat#A8010
Sequencing grade trypsin	Promega	Cat#V5111
DL-Dithiothreitol	Sigma-Aldrich	Cat#D5546-5G
Iodoacetamide	Sigma-Aldrich	Cat#I1149-5G
IPTG	BioBasic	Cat#IB0168
Recombinant protein: human SCAPER-FLAG	This study	N/A
Recombinant protein: human FLAG-SCAPER	This study	N/A
Recombinant protein: human 6xHis-TEV-Twin-Strep-TTC5	This study	N/A
Recombinant protein: human 6xHis-TEV-Twin-Strep-TTC5-K97A	This study	N/A
Critical commercial assays		
iScript cDNA synthesis kit	BioRad	Cat#1708891
KAPA SYBR Fast qPCR reagents	Sigma-Aldrich	Cat#KK4602
TaqMan Fast Advanced Master Mix	Thermo Fisher	Cat#4444557
PureLink RNA Mini Kit	Thermo Fisher	Cat#12183018A
PureLink DNase Set	Thermo Fisher	Cat#12185010
RNeasy Plus mini kit	QIAGEN	Cat#74134
SuperScript IV kit	Invitrogen	Cat#18091050
PowerUp SYBR Green master mix	Thermo Fisher	Cat#A25776
USB Poly(A) Tail-Length Assay Kit	Thermo Fisher	Cat#764551KT
Deposited data		
Human Reviewed UniProt Fasta database (2019)	UniProt	https://www.uniprot.org/
Structure: TTC5 bound to 60S ribosome subunit and tubulin	Lin et al.[17]	PDB: 6T59
Structure: TTC5 and SCAPER bound to 60S ribosome and tubulin (model)	This study	PDB: 8BPO
Structure: TTC5 and SCAPER bound to 60S ribosome and tubulin (EM-map)	This study	EMDB: EMD-16155
Proteomics: TurboID-TTC5 and TurboID-SCAPER datasets	This study	PRIDE: PXD041096

(*Continued on next page*)

Continued

REAGENT or RESOURCE	SOURCE	IDENTIFIER
Experimental models: Cell lines		
Human: Flp-In T-REx 293	Thermo Fisher	Cat#R78007; RRID:CVCL_U427
Human: HeLa Flp-In T-REx	Thermo Fisher/S. Shao lab, Harvard Medical School	Cat#R71407
Human: Expi293F	Thermo Fisher	Cat#A14527; RRID:CVCL_D615
HEK293 T-REx TTC5-KO	Lin et al.[17]	Internal ID: cMH3
HEK293 T-REx TTC5-KO + GFP-TTC5	This study	Internal ID: cMH2
HEK293 T-REx TTC5-KO + GFP-TTC5-K97A	This study	Internal ID: cMH14
HEK293 T-REx TTC5KO clone G2T9 + TurboID-FLAG-TTC5	This study	Internal ID: cMH10
HEK293 T-REx TTC5KO clone G2T9 + TurboID-FLAG-TTC5-K97A	This study	Internal ID: cMH17
HEK293 T-REx SCAPER-KO sgRNA1 cl.1	This study	Internal ID: cMH25-1
HEK293 T-REx SCAPER-KO sgRNA1 cl.5	This study	Internal ID: cMH25-5
HEK293 T-REx SCAPER-KO sgRNA3 cl.3	This study	Internal ID: cMH26-3
HEK293 T-REx SCAPER-KO sgRNA3 cl.6	This study	Internal ID: cMH26-3
HEK293 T-REx SCAPER-KO sgRNA1 cl.1 + SCAPER-FLAG	This study	Internal ID: cMH29
HEK293 T-REx SCAPER-KO sgRNA1 cl.1 + FLAG-SCAPER	This study	Internal ID: cMH30
HEK293 T-REx SCAPER-KO sgRNA1 cl.1 + FLAG-SCAPER-RSL-AAA	This study	Internal ID: cMH33
HEK293 T-REx SCAPER-KO sgRNA1 cl.1 + FLAG-SCAPER-aa351-1400	This study	Internal ID: cMH34
HEK293 T-REx SCAPER-KO sgRNA1 cl.1 + FLAG-SCAPER-E620Δ	This study	Internal ID: cMH35
HEK293 T-REx SCAPER-KO sgRNA1 cl.1 + FLAG-SCAPER-E620A	This study	Internal ID: cMH36
HEK293 T-REx SCAPER-KO sgRNA1 cl.1 + FLAG-SCAPER-E675-K677Δ	This study	Internal ID: cMH37
HEK293 T-REx SCAPER-KO sgRNA1 cl.1 + FLAG-SCAPER-E675-K677-AAA	This study	Internal ID: cMH38
HEK293 T-REx SCAPER-KO sgRNA1 cl.1 + FLAG-SCAPER-S1219N	This study	Internal ID: cMH39
HEK293 T-REx SCAPER-KO sgRNA1 cl.1 + FLAG-SCAPER-E1338K	This study	Internal ID: cMH43
HEK293 T-REx SCAPER-KO sgRNA1 cl.1 + FLAG-SCAPER-1338-EMS-AAA	This study	Internal ID: cMH44
HEK293 T-REx SCAPER-KO sgRNA1 cl.1 + FLAG-SCAPER-R907E_K910E	This study	Internal ID: cMH45
HEK293 T-REx SCAPER-KO sgRNA1 cl.1 + FLAG-SCAPER-R934A_R941A	This study	Internal ID: cMH46
HEK293 T-REx SCAPER-KO sgRNA1 cl.1 + FLAG-SCAPER-ES*4E	This study	Internal ID: cMH49
HEK293 T-REx SCAPER-KO sgRNA1 cl.1 + FLAG-SCAPER-ES*7E	This study	Internal ID: cMH48
HEK293 T-REx SCAPER-KO sgRNA1 cl.1 + TurboID-FLAG-SCAPER	This study	Internal ID: cMH50
HEK293 T-REx SCAPER-KO sgRNA1 cl.1 + TurboID-FLAG-SCAPER_E620Δ	This study	Internal ID: cMH52
HEK293 T-REx SCAPER-KO sgRNA1 cl.1 + FLAG-SCAPER_F628S_L632S	This study	Internal ID: cMH67

(*Continued on next page*)

Continued

REAGENT or RESOURCE	SOURCE	IDENTIFIER
HEK293 T-REx SCAPER-KO sgRNA1 cl.1 + FLAG-SCAPER_F628S_I629K_L632S	This study	Internal ID: cMH68
HEK293 T-REx SCAPER-KO sgRNA1 cl.1 + FLAG-SCAPER_E618K_E625K	This study	Internal ID: cMH69
HEK293 T-REx SCAPER-KO sgRNA1 cl.1 + FLAG-SCAPER_E633K_D640K	This study	Internal ID: cMH70
HEK293 T-REx WT + CNOT8-TST_siRNA-resistant	This study	Internal ID: cMH72
HEK293 T-REx WT + CNOT8-TST_D40A_siRNA-resistant	This study	Internal ID: cMH73
HEK293 T-REx CNOT11-KO sgRNA AC cl.12	This study	Internal ID: cMH75-12
HEK293 T-REx CNOT11-KO cl.12 + 3HA-TEV-CNOT11	This study	Internal ID: cMH76
HEK293 T-REx CNOT11-KO cl.12 + 3HA-TEV-CNOT11_V454S-L405S	This study	Internal ID: cMH79
HEK293 T-REx CNOT11-KO cl.12 + 3HA-TEV-CNOT11_V454S_L405S_L451S	This study	Internal ID: cMH80
HEK293 T-REx CNOT11-KO cl.12 + 3HA-TEV-CNOT11_R447E_R450E	This study	Internal ID: cMH81
HEK293 T-REx CNOT11-KO cl.12 + 3HA-TEV-CNOT11_R461E_R485E	This study	Internal ID: cMH82
HeLa T-REx SCAPER-KO	This study	N/A (same as cell line name)
HeLa T-REx SCAPER-KO + FLAG-SCAPER-RSL-AAA	This study	N/A (same as cell line name)
HeLa T-REx SCAPER-KO + FLAG-SCAPER-aa351-1400	This study	N/A (same as cell line name)
HeLa T-REx SCAPER-KO + FLAG-SCAPER-E620Δ	This study	N/A (same as cell line name)
**Oligonucleotides**		
Oligonucleotides used in this study	This study	Table S5
**Recombinant DNA**		
pcDNA5/FRT/TO Vector	Thermo Fisher	Cat#V652020
pOG44 Flp-Recombinase Expression Vector	Thermo Fisher	Cat#V600520
pet28a 6xHis-TEV-Twin-Strep-TTC5	Lin et al.[17]	Internal ID: ZL53
pet28a 6xHis-TEV-Twin-Strep-TTC5-K97A	This study	Internal ID: ZL110
pcDNA5 FRT/TO GFP-TTC5	This study	Internal ID: ZL140
pcDNA3.1 TUBB	Lin et al.[17]	Internal ID: ZL54
pcDNA3.1 TUBB-MHQV mutant	Lin et al.[17]	Internal ID: ZL59
pcDNA5/FRT/TO N-GFP-TTC5_K97A	This study	Internal ID: pMH500
pcDNA5/FRT/TO TurboID-FLAG-TTC5	This study	Internal ID: pMH485
pcDNA5/FRT/TO TurboID-FLAG-TTC5_K97A	This study	Internal ID: pMH512
px459 SCAPER_sgRNA1_exon5	This study	Internal ID: pMH568
px459 SCAPER_sgRNA3_exon17	This study	Internal ID: pMH569
pcDNA3.1 SCAPER-FLAG	Genscript	ID: OHu03552
pcDNA5/FRT/TO FLAG-SCAPER	This study	Internal ID: pMH575
pcDNA5/FRT/TO FLAG-SCAPER-RSL-AAA	This study	Internal ID: pMH579
pcDNA5/FRT/TO FLAG-SCAPER_aa1-935	This study	Internal ID: pMH580
pcDNA5/FRT/TO FLAG-SCAPER_aa1-726	This study	Internal ID: pMH581
pcDNA5/FRT/TO FLAG-SCAPER_aa351-1400	This study	Internal ID: pMH583
pcDNA5/FRT/TO FLAG-SCAPER_aa701-1400	This study	Internal ID: pMH584
pcDNA5/FRT/TO FLAG-SCAPER_aa901-1400	This study	Internal ID: pMH585

(*Continued on next page*)

CellPress
OPEN ACCESS

Continued

REAGENT or RESOURCE	SOURCE	IDENTIFIER
pcDNA5/FRT/TO FLAG-SCAPER-E620Δ	This study	Internal ID: pMH586
pcDNA5/FRT/TO FLAG-SCAPER-E620A	This study	Internal ID: pMH587
pcDNA5/FRT/TO FLAG-SCAPER-E675-K677Δ	This study	Internal ID: pMH588
pcDNA5/FRT/TO FLAG-SCAPER-E675-K677-AAA	This study	Internal ID: pMH589
pcDNA5/FRT/TO FLAG-SCAPER-S1219N	This study	Internal ID: pMH590
pcDNA5/FRT/TO FLAG-SCAPER-E1338K	This study	Internal ID: pMH598
pcDNA5/FRT/TO FLAG-SCAPER-1338-1340-EMS-AAA	This study	Internal ID: pMH599
pcDNA5/FRT/TO FLAG-SCAPER-R907E_K910E	This study	Internal ID: pMH601
pcDNA5/FRT/TO FLAG-SCAPER-R934A_R941A	This study	Internal ID: pMH603
pcDNA5/FRT/TO FLAG-SCAPER-ES*7E	This study	Internal ID: pMH607
pcDNA5/FRT/TO FLAG-SCAPER-ES*4E	This study	Internal ID: pMH608
pcDNA5/FRT/TO TurboID-FLAG-SCAPER	This study	Internal ID: pMH609
pcDNA5/FRT/TO TurboID-FLAG-SCAPER-E620Δ	This study	Internal ID: pMH611
pcDNA5/FRT/TO FLAG-SCAPER_F628S_L632S	This study	Internal ID: pMH632
pcDNA5/FRT/TO FLAG-SCAPER_F628S_L632S_I629K	This study	Internal ID: pMH633
pcDNA5/FRT/TO FLAG-SCAPER_E618K_E625K	This study	Internal ID: pMH634
pcDNA5/FRT/TO FLAG-SCAPER_E633K_D640K	This study	Internal ID: pMH635
pcDNA5/FRT/TO CNOT8-TST-siRNA-resistant	This study	Internal ID: pMH644
pcDNA5/FRT/TO CNOT8-TST-D40A-siRNA-resistant	This study	Internal ID: pMH645
pcDNA5/FRT/TO 3HA-TEV-CNOT11	This study	Internal ID: pMH646
pcDNA5/FRT/TO 3HA-TEV-CNOT11_V454S-L405S	This study	Internal ID: pMH649
pcDNA5/FRT/TO 3HA-TEV-CNOT11_V454S_L405S_L451S	This study	Internal ID: pMH650
pcDNA5/FRT/TO 3HA-TEV-CNOT11_R447E_R450E	This study	Internal ID: pMH651
**Software and algorithms**		
TIDE online tool	Brinkman et al.[72]	http://shinyapps.datacurators.nl/tide/
Fiji	Schindelin et al.[73]	https://fiji.sc/
GraphPad Prism 9	GraphPad	https://www.graphpad.com/
Microsoft Excel	Microsoft	https://www.microsoft.com
Quantstudio Real-time PCR software v1.3	Thermo Fisher	https://www.thermofisher.com
R v4.2.0	R Foundation for Statistical Computing	https://www.r-project.org/
ImageLab 6.1	BioRad	https://www.bio-rad.com/en-uk/product/image-lab-software?ID=KRE6P5E8Z
MaxQuant v1.6.6.0 and v1.6.17.0	Cox and Mann[74]	https://www.maxquant.org/maxquant
Perseus v1.6.6.0 and v1.6.17.0	Tyanova et al.[75]	https://maxquant.org/perseus/
RELION 4	Kimanius et al.[76]	https://github.com/3dem/relion
Coot v0.9.6, Marina Bay	Emsley et al.[77]	https://www2.mrc-lmb.cam.ac.uk/personal/pemsley/coot/
Phenix v1.20-4459-000	Adams et al.[78]	https://phenix-online.org/
UCSF Chimera v1.15	Pettersen et al.[79]	https://www.cgl.ucsf.edu/chimera/
UCSF ChimeraX v1.3	Goddard et al.[80]	https://www.cgl.ucsf.edu/chimerax/
AlphaFold2	Jumper et al.[38]	https://github.com/deepmind/alphafold
AlphaFold2 multimer	Evans et al.[39]	https://github.com/deepmind/alphafold
Colabfold v1.2.0	Mirdita et al.[81]	https://github.com/sokrypton/ColabFold
PyMOL v2.4	Schrödinger, LLC	https://pymol.org/2/
ConSurf Web Server	Ashkenazy et al.[82]	https://consurf.tau.ac.il/consurf_index.php
Clustal Omega	Sievers et al.[83]	https://www.ebi.ac.uk/Tools/msa/clustalo/

(*Continued on next page*)

Continued		
REAGENT or RESOURCE	SOURCE	IDENTIFIER
Jalview v2.11.2.6	Waterhouse et al.[84]	https://www.jalview.org/
Adobe Photoshop	Adobe	RRID:SCR_014199; https://www.adobe.com/products/photoshop.html
Adobe Illustrator	Adobe	RRID:SCR_010279; http://www.adobe.com/products/illustrator.html
SnapGene v5.1.7	SnapGene	https://www.snapgene.com/
Other		
DMEM, high glucose without L-methionine	Sigma-Aldrich	Cat#D0422
DMEM, high glucose, GlutaMAX Supplement, pyruvate	Gibco/Thermo Fisher	Cat#10569010
Liebowitz-15 without phenol-red	Thermo Fisher	Cat#21083027
Fetal Bovine Serum	Thermo Fisher	Cat#10270106
Expi293 Expression Medium	Thermo Fisher	Cat#A1435101
Rabbit Reticulocyte Lysate Mix	Sharma et al.[85]	N/A
Pierce ECL Western Blotting Substrate	Thermo Fisher	Cat#32209
SuperSignal West Pico PLUS Chemiluminescent Substrate	Thermo Fisher	Cat#34080
Streptavidin Magnetic beads	Pierce	Cat#88817
Anti-FLAG M2 affinity gel	Sigma-Aldrich	Cat#A2220; RRID:AB_10063035
MagStrep "type 3" XT beads	IBA	Cat#2-4090-010
Strep-Tactin Sepharose	IBA	Cat#2-1201-010
Ni-NTA agarose	QIAGEN	Cat#30210
UltrAuFoil R1.2/1.3 300-mesh grids	Quantifoil	Cat#N1-A14nAu30-50
C18 3M Empore	3M	Cat#2215-C18
Poros Oligo R3	Thermo Fisher	Cat#1-1339-03
TMTpro 18-plex reagents	Thermo Fisher	Cat#A44520 (TMTpro 16plex) + Cat#A52046 134C & 135N

RESOURCE AVAILABILITY

Lead contact

Further information and requests for resources and reagents should be directed to and will be fulfilled by the lead contact, Ramanujan S. Hegde (rhegde@mrc-lmb.cam.ac.uk).

Materials availability

All unique/stable materials generated in this study are available upon request from the lead contact.

Data and code availability

- Cryo-EM maps are deposited to the Electron microscopy database (EMDB) and models in the Protein Data Bank (PDB). The mass spectrometry proteomics data have been deposited to the ProteomeXchange Consortium via the PRIDE partner repository. Accession numbers and DOI are listed in the key resources table.
- This paper does not report original code.
- Any additional information required to reanalyze the data reported in this paper is available from the lead contact upon request.

EXPERIMENTAL MODEL AND STUDY PARTICIPANT DETAILS

Cell lines

Flp-In T-REx HEK 293 or HeLa cells (Thermo Fisher) were maintained at 37°C with 5% CO_2 in DMEM with GlutaMAX and 4.5 g/l glucose (Gibco) supplemented with 10% fetal calf serum, and optionally 0.1 mg/ml Hygromycin B and 10 μg/ml Blasticidine S for stable Flp-In cell lines. All cell lines used are female, routinely checked for mycoplasma contamination, and not authenticated further.

CellPress
OPEN ACCESS

METHOD DETAILS

Plasmids and reagents

β-tubulin (human TUBB) constructs for in vitro translation have been described previously.[17] EGFP-tagged TTC5 (“GFP-TTC5”) was obtained by cloning previously described TTC5 constructs[17] into a pcDNA5/FRT/TO with an N-terminal EGFP tag. N-terminally 6xHis-TEV-Twin-Strep-tagged TTC5 (“Strep-TTC5”) for bacterial expression was cloned in the pET-28a vector. A human SCAPER cDNA construct with C-terminal FLAG-tag in a pcDNA3.1 vector was obtained from Genscript (cloneID OHu03552) and subsequently cloned into pcDNA5/FRT/TO vectors with N- or C-terminal FLAG-tags. TurboID-FLAG was fused to the N-terminus of TTC5 or SCAPER (WT or mutants) and cloned into pcDNA5/FRT/TO vectors. Human siRNA-resistant CNOT8-WT and -CD were cloned from synthetic gene blocks (IDT) into pcDNA5/FRT/TO with a C-terminal PreScission cleavage site followed by a Twin-Strep-tag. Human CNOT11 was cloned from HEK293 T-REx cDNA into pcDNA5/FRT/TO with an N-terminal 3HA-TEV-tag. CRISPick (https://portals.broadinstitute.org/gppx/crispick/public) was used to design sgRNAs for CRISPR-Cas9-mediated knockout (KO) of SCAPER and CNOT11. The sequences are listed in Table S5.

Cell culture procedures

Flp-In T-REx HEK 293 or HeLa cells (Thermo Fisher) were maintained at 37°C with 5% CO_2 in DMEM with GlutaMAX and 4.5 g/l glucose (Gibco) supplemented with 10% fetal calf serum, and optionally 0.1 mg/ml Hygromycin B and 10 μg/ml Blasticidine S for stable Flp-In cell lines. CRISPR-Cas9 mediated gene knockout for SCAPER was performed essentially as described[86]: HeLa or HEK293 Flp-In TRex cells were transiently transfected with the pX459 plasmid encoding the sgRNAs targeting SCAPER and Cas9, using Lipofectamine 3000 reagent (Invitrogen) for HeLa cells or TransIT-293 (Mirus) for HEK T-REx cells following manufacturers' protocols. 24 hours after transfection, 2 μg/ml puromycin (1μg/ml for HEK293) was added for selection. 2–3 days after transfection, cells were trypsinized and re-plated in 96-well plates at a density of 0.5 or 1 cell per well using a FACSAria Fusion instrument (BD) to obtain single cell clones. To obtain CNOT11-KO clones, IDT Alt-R sgRNA was complexed with Alt-R S.p. Cas9 Nuclease V3 and transfected into HEK T-REx cells using Lipofectamine RNAiMAX (Invitrogen) according to the IDT user guide. Cells were grown for 48 hours and then sorted into 96-well plates.

Successful knockout clones were verified by genotyping via PCR amplification of the modified region followed by TIDE analysis[72] and western blotting. See Figures S3B–S3D for a detailed characterization of SCAPER-KO cells. Throughout the rest of the study, we used SCAPER-KO sgRNA1 clone 1 for all experiments and to generate rescue cell lines. Rescue cell lines with stable expression of TTC5, SCAPER, CNOT8 or CNOT11 constructs were generated in knockout cells (or wild type cells for CNOT8) using the Flp-In system (Invitrogen) following manufacturer's protocol. Expression of transgenes was induced with 200 ng/mL (HeLa) or 1 μg/ml (HEK T-REx) doxycycline for 24–48 hours. Colchicine (10 μM), Nocodazole (10 μM), and combretastatin A4 (CA4, 100 nM) treatments were performed in standard media for 3 h, unless stated otherwise. All drugs gave similar effects in autoregulation assays, but we found results with colchicine more variable and hence used CA4 throughout most of the study, which gave consistent results.

For siRNA mediated knockdowns of indicated genes, Silencer Select siRNAs (Thermo Fisher) were transfected using RNAiMAX (Invitrogen) according the manufacturer's instructions for reverse transfection (see Table S5). Cells were typically incubated for three days, unless stated otherwise. When multiple siRNAs were transfected, they were used in equal ratios with the total amount of siRNA kept constant.

Live cell imaging and data analysis

Flp-In T-REx HeLa cells of the genotypes indicated in the figure legends were plated in 8-well Lab Tek II Chamber 1.5 German coverglass dishes (Thermo Fisher, 155409) in regular growth medium, and incubated for 6 hours. Medium was then changed to Liebowitz-15 without phenol-red (Thermo Fisher, 21083027) supplemented with 10% fetal calf serum, 200 ng/mL doxycycline and 50 nM Sir-DNA (Cytoskeleton, CY-SC007). Cells were incubated for 24 hours prior to imaging. Time lapse images were acquired using Nikon Eclipse Ti2-E inverted microscope (Nikon), equipped with Kinetix sCMOS camera (Photometrics), Spectrax Chroma light engine for fluorescence illumination (Lumencor), or a Nikon Ti / CSU-W1 Spinning Disc Confocal microscope (Nikon), equipped with Photometrics Prime 95B camera (Photometrics) and 3iL35 LaserStack (Intelligent Imaging Innovations Inc). Both systems are equipped with a perfect focus system, and an incubation chamber with 37°C and controlled humidity (OkoLab). Three-dimensional images at multiple stage positions were acquired in steps of 2 μm, every 7 minutes for 10 hours using NIS Elements (Nikon) and 20x Plan Apochromat Lambda objective (NA 0.80, Nikon) or 40x Plan Apochromat Lambda objective (NA 0.95, Nikon). Maximum intensity projections and inverted color profiles of representative examples of mitoses were prepared in Fiji and exported as still images. Analysis of mitotic cells was performed using 3D reconstructions in Fiji. The parameters scored (based on the Sir-DNA signal) were: occurrence of unaligned chromosomes in metaphase, and chromosome segregation errors in anaphase. Analyses of 100 cells per cell line in three biological replicates were documented using Excel and processed and plotted using GraphPad Prism software. Instances where not all the chromosomes were properly aligned on the spindle equator in metaphase and/or anaphase are classified as chromosome alignment errors. Instances where sister chromatids failed to properly separate, either segregating both into the same daughter cell or forming a bridge in anaphase were classified as segregation errors. Numbers reported represent percentage of cells experiencing either abnormality.

Western blot analysis

For analysis of protein expression levels in HEK T-REx cell lines, cells were typically processed in parallel to cells used for autoregulation assays in 12 or 24 well plates, and protein expression was induced by addition of 1 μg/ml doxycycline for 24–48h. Cells were washed with PBS once and then harvested in PBS, pelleted and lysed in 1% SDS, 100 mM Tris pH8 by boiling for 20 minutes at 95°C. Samples were normalized, separated on 7% or 10% Tris-Tricine based gels, and transferred to 0.2 μm nitrocellulose membrane (BioRad). Membranes were stained with Ponceau S (Sigma), blocked in 5% milk (or 3% BSA for Streptavidin-HRP blots) and incubated with primary antibody at 4°C overnight or for 1h at room temperature as listed below. Signals were detected using HRP-conjugated secondary antibodies and chemiluminescent substrate Pierce ECL or SuperSignal West Pico PLUS (Thermo Fisher). As loading controls, membranes were probed with antibodies against β-actin, RPL8 or GAPDH. Alternatively, the Ponceau S stained membrane is displayed.

For total protein analysis of HeLa cells, parental HeLa T-REx, SCAPER knockout and the indicated rescue cell lines were grown in 6 well plates and treated with 200 ng/ml doxycycline for 24 hours, then washed with PBS and collected by scraping directly in Laemmli buffer. Total cell lysates were boiled for 5 minutes, equal volumes loaded on a Tris-Glycine 4-12% gel (ThermoFisher Scientific, XP04125BOX), and transferred in the presence of 0.1% SDS to nitrocellulose membrane. The membrane was incubated with blocking solution (5% non-fat dry milk in PBS-0.2% Tween 20) and then exposed to primary antibodies against FLAG-tag and GAPDH. The membrane was further incubated with HRP-conjugated secondary antibodies against mouse (ThermoFisher Scientific, 31430) and rabbit (ThermoFisher Scientific, 31460) at 1:10.000 dilution and visualized by ECL (ThermoFisher Scientific, 34580) using an Amersham ImageQuant 800 imaging system.

mRNA quantification by RT-qPCR

For autoregulation assays in HEK T-REx cells, cells were grown to 70–80% confluency (optionally with 1 μg/ml doxycycline for 24–48 h) in 24- or 12-well plates and treated with colchicine (10 μM, Sigma PRH1764), combretastatin A4 (CA4, Selleckchem S7783), or as controls (DMSO/regular media) for 3 hours. Cells were washed with PBS, harvested and total RNA was isolated using the RNeasy Plus mini kit (QIAGEN, 74134) as per the manufacturers protocol. 500 ng of total RNA was used to generate cDNA using the iScript cDNA synthesis kit (BioRad 1708891). Samples were diluted ten-fold with nuclease-free water, or kept at higher concentrations to make a standard curve. RT-qPCR was carried out using a ViiA 7 Real-Time PCR System (Thermo Fisher Scientific) and KAPA SYBR Fast qPCR reagents (KAPA Biosystems) as per manufacturer's instructions. The primer sequences used are listed in Table S5. All pairs of primers were annealed at 60°C, and a melt curve performed. PCR products were verified by sequencing. Data was then analyzed using the Quantstudio Real-time PCR software v1.3. Relative standard curve quantification was performed and values were normalized to RPLP1 levels, and to untreated control samples. Processing, statistical analysis, and data plotting were performed in Microsoft Excel and GraphPad Prism.

For analysis of previously reported CCR4-NOT substrates, untreated control cDNA samples from siRNA knockdown experiments were reanalysed using TaqMan probes (Thermo Fisher) and TaqMan Fast Advanced Master Mix according to the manufacturer's protocols. FAM-MGB labelled probes (Cat No 4331182) for TRIB3 (ID: Hs00221754_m1), FZD8 (ID: Hs00259040_s1), LEFTY2 (ID: Hs00745761_s1) and 18S rRNA (ID: Hs99999901_s1) were analyzed in multiplex-reactions with a VIC-MGB labelled GAPDH probe (Cat No 4326317E, ID: Hs99999905_m1). A standard curve was prepared from CNOT1-KD samples. Samples were normalized to GAPDH as an endogenous control for each well, and relative standard curve quantification was performed using the Quantstudio Real-time PCR software v1.3.

For autoregulation assays in HeLa cells, Flp-In TRex HeLa parental, SCAPER knockout and the indicated rescue cell lines were grown to 70–80% confluency in 10 cm dishes and treated with DMSO (control) or combretastatin A4 (100 nM) 4 hours. Cells were harvested and total RNA isolated using the PureLink RNA Mini Kit (Invitrogen, Thermo Fisher, 12183018A) as per manufacturer's protocol. On column DNase digestion was performed using PureLink DNase Set (Thermo Fisher, 12185010) as per manufacturer's instructions. 500 ng of total RNA was used to generate cDNA using the SuperScript IV kit (Invitrogen, 18091050) and random hexamer primers following the manufacturer's protocol. RT-qPCR was carried out using 5 ng of cDNA and 2x PowerUp SYBR Green master mix (Thermo Fisher, A25776) on a thermocycler (BioRad), as per manufacturer's instructions. Data analysis was performed using the ddCt method.[87] All data were normalized to reference genes RPLP1 or GAPDH, and to DMSO treated controls. Experiments include two biological replicates. Processing and data plotting were performed in R, Microsoft Excel, and GraphPad Prism.

For measurement of tubulin mRNA decay rates in HEK T-REx cells, cells were grown to 70–90% confluencey and treated with 5 μg/ml actinomycin D (Sigma-Aldrich, A1410), and optionally with 100 nM CA4 or DMSO for 6 hours. At the indicated time-points, samples were harvested and mRNA isolation, reverse transcription and qPCR were performed as described above. TUBA1B and TUBB mRNAs were normalized to GAPDH mRNA and to the $t = 0$ timepoint. Data processing, statistical analysis, and plotting were performed in Microsoft Excel and GraphPad Prism.

Poly(A) tail-length assay

Poly(A) tail lengths of TUBA1B and GAPDH were measured using a poly(A) tail-length assay kit (Thermo Fisher). HEK T-REx cells were grown to 70–90% confluency and optionally treated with 100 nM CA4 for 3h. Total RNA was isolated as described above and the 3' ends of mRNAs were by extended with guanosine/inosine (G/I) tails using kit reagents. After reverse transcription, PCR amplification was performed using a gene-specific forward primer (to either TUBA1B or GAPDH as a control) and a universal reverse primer that

anneals to the poly(A)-G/I fusion site. As a PCR control and tail-lacking size marker, a gene-specific reverse primer that anneals ~70 nt upstream of the poly(A) tail in the 3'UTR was used. All procedures were performed according to the manufacturer's instructions. PCR products were separated on 2.5% agarose TBE gels and stained with SYBR Safe (Thermo Fisher). Gene-specific forward and reverse primers for TUBA1B and GAPDH are listed in Table S5.

Pulse labelling of protein synthesis

To measure tubulin autoregulation by pulse labelling of protein synthesis, HEK T-REx wild type or SCAPER-KO cells were seeded in 12-well plates and transfected the next day with pcDNA5/FRT/TO rescue plasmids and a puromycin-resistance conferring plasmid (MXS-CMV-PuroR) using TransIT-293 (Mirus). 24 hours after transfection, cells were induced and selected by addition of 1 μg/ml doxycycline and 1 μg/ml puromycin, respectively. 24 hours after induction, cells were treated with 100 nM CA4 (or left untreated) for 3 hours. Cells were then washed with warm PBS and harvested in PBS. 40% of cells were used for total protein analysis, and 60% of cells were resuspended in depletion media lacking FCS and methionine (+/- 100 nM CA4). Cells were starved for 30 minutes at 37°C and pulse labelling was performed for 30 minutes at 37°C by addition of ^{35}S-methionine at 100 μCi/ml. After labelling, cells were pelleted (5000 rpm, 2 min) and lysed in 45 μl digitonin lysis buffer [50 mM HEPES pH7.4, 100 mM KAc, 5 mM $MgAc_2$, 1 mM DTT, 1x EDTA-free protease inhibitor cocktail (Roche), 0.01% digitonin] for 10 minutes on ice. Lysates were cleared by centrifugation at maximum speed at 4°C in a table-top centrifuge. 1 μl sample was mixed with sample buffer and separated on 10% Tris-Tricine gels to analyze proteins by autoradiography. Quantification was performed using ImageLab software (BioRad). The tubulin band was normalized to an unrelated band for each lane and then to untreated control samples. Microsoft Excel and GraphPad Prism were used to plot data. Two independent replicates were averaged.

Biotin proximity labelling procedure

For biotin proximity labelling experiments,[36,88] TurboID-FLAG was fused to the N-terminus of TTC5 or SCAPER (WT or mutants) and cloned into pcDNA5/FRT/TO vectors. The eGFP-V5-TurboID vector was kind gift from the Bienz lab (MRC-LMB). TTC5 or SCAPER KO HEK T-REx cell lines were rescued by stable integration of TurboID constructs, which were functional in autoregulation assays. To avoid strong overexpression, leaky expression from the doxycycline-inducible promoter was used for TurboID-TTC5 expression, and TurboID-SCAPER was induced with 2 ng/ml doxycycline for 48 hours. Parental cell lines without TurboID constructs served as specificity controls for mass spectrometry.

To isolate biotinylated proteins for mass spectrometry analysis, cells were seeded in 150 mm plates and grown to ~ 80% confluency. For TurboID-TTC5, two plates per replicate were pretreated with DMSO (control), colchicine (10 μM, Sigma PRH1764) or nocodazole (10 μM, Sigma SML1665) for 30 minutes and biotin (APExBIO A8010) was added at 50 μM and incubated for another 2.5 hours. For SCAPER, one plate of cells per replicate was treated with DMSO (control) or combretastatin A4 (Selleckchem S7783) for 30 minutes and biotin was added at 50 μM and incubated for another 30 minutes. Cells were washed once in ice-cold PBS, pelleted, and cytosolic extracts were prepared by lysis in 1 ml digitonin lysis buffer per 150 mm plate for 10–15 min on ice [50 mM HEPES pH7.4, 100 mM Kac (400 mM KAc for TurboID-SCAPER samples), 5 mM $MgAc_2$, 1 mM DTT, 1x EDTA-free protease inhibitor cocktail (Roche), 0.01% digitonin]. Lysates were cleared by centrifugation at maximum speed at 4°C in a table-top centrifuge. Lysates were then incubated on a rotating wheel with ~ 50 μl of streptavidin-coupled magnetic beads (Pierce 88817) for 2 hours at 4°C. Beads were then washed with 1 ml each of physiological salt buffer [PSB: 50 mM HEPES pH7.4, 100 mM KAc (400 mM for TuroboID-SCAPER samples), 2 mM $MgAc_2$] with 0.01% digitonin, wash buffer 1 (1% SDS, 10 mM Tris-HCl pH8), wash buffer 2 (1 M NaCl, 10 mM HEPES pH7.4, 0.01% digitonin), and wash buffer 3 (2 M urea, 10 mM Tris-HCl pH8, 0.01% digitonin). To remove detergent, beads were washed twice with 100 μl 50 mM Tris-HCl pH8, 150 mM NaCl and transferred to a new tube with the last step. Beads were then stored in 20 μl 50 mM Tris-HCl pH8, 150 mM NaCl for mass spectrometry analysis, or eluted with 20 μl sample buffer supplemented with 2 mM biotin for 5 minutes at 95°C for analysis by SDS-PAGE. For mass spectrometry analysis, two or three biological replicates were processed for each condition.

For western blot validation of SCAPER biotinylation by TurboID-TTC5, expression in the indicated cell lines was induced with 1 μg/ml doxycycline and cells were transfected with a pcDNA3-SCAPER-FLAG construct using TransIT293 (Mirus) in 10 cm dishes. All plates were pretreated with colchicine (10 μM 30 minutes) and biotin was added for another 2.5 h (50 μM). Biotinylated proteins were isolated as described above.

Quantitative proteomics procedures

On-bead digestion

Proteins bound to beads were reduced with 2 mM DTT in 2 M urea buffer and sequencing grade trypsin (Promega) was added to a final concentration of 5 ng/μl. After incubation for 3 h at 25°C, supernatants were transferred to fresh eppendorf tubes. Beads were washed once with 2M urea buffer, once with 1M urea buffer, and the washes were combined with the corresponding supernatants. Samples were then alkylated with 4 mM iodoacetamide (IAA) in the dark at 25°C for 30 min. An additional 0.1 μg of trypsin (Promega) was added to the samples and digested over night at 25°C. Samples were acidified to 0.5% formic acid (FA) and desalted using home-made C18 (3M Empore) stage tips filled with 4 μl of Poros Oligo R3 resin (Thermo Fisher). Bound peptides were eluted sequentially with 30%, 50% and 80% acetonitrile (MeCN) in 0.5% FA and lyophilized.

Tandem mass tag (TMT) labeling

Dried peptides from each condition were resuspended in 15 μl of 200 mM HEPES, pH 8.5. 7.5 μl of TMTpro 18-plex reagent (Thermo Fisher Scientific), reconstituted in anhydrous acetonitrile according to manufacturer's instructions, was added and incubated at room temperature for 1 h. The labeling reactions were terminated by incubation with 1.5 μl of 5% hydroxylamine for 30 min. Labeled samples for each condition were pooled into one sample, and MeCN was removed by vacuum centrifugation. TMT-labeled peptides were desalted and then fractionated with home-made C18 stage tip using 10 mM ammonium bicarbonate and increasing acetonitrile concentration. Eluted fractions were acidified, partially dried down in a speed vac and used for LC-MS/MS.

Mass spectrometry analysis

The fractionated peptides were analysed by LC-MS/MS using a fully automated Ultimate 3000 RSLC nano System (Thermo Fisher Scientific) fitted with a 100 μm x 2 cm PepMap100 C18 nano trap column and a 75 μm × 25cm, nanoEase M/Z HSS C18 T3 column (Waters). Peptides were separated using a binary gradient consisting of buffer A (2% MeCN, 0.1% FA) and buffer B (80% MeCN, 0.1% FA). Eluted peptides were introduced directly via a nanospray ion source into a Q Exactive Plus hybrid quardrupole-Orbitrap mass spectrometer (MS2, TurboID-TTC5 samples) or Orbitrap Eclipse mass spectrometer (RTS-MS3, TurboID-SCAPER samples), both from Thermo Fisher Scientific. The Q Exactive Plus mass spectrometer was operated in standard data dependent mode, performed MS1 full-scan at m/z = 380-1600 with a resolution of 70K, followed by MS2 acquisitions of the 15 most intense ions with a resolution of 35K and NCE of 29%. MS1 target values of 3e6 and MS2 target values of 1e5 were used. Dynamic exclusion was enabled for 40s.

For the RTS-MS3 experiment (TurboID-SCAPER samples), MS1 spectra were acquired using the following settings: Resolution=120K; mass range=400-1400m/z; AGC target=4e5 and dynamic exclusion was set at 60s. MS2 analysis were carried out with HCD activation, ion trap detection, AGC=1e4; NCE=33% and isolation window =0.7m/z. RTS of MS2 spectrum was set up to search uniport Human proteome (2021), with fixed modifications cysteine carbamidomethylation and TMTpro 16plex at N-terminal and lysine residues. Met-oxidation was set as variable modification. Missed cleavage=1 and maximum variable modifications=2. In MS3 scans, the selected precursors were fragmented by HCD and analyzed using the orbitrap with these settings: Isolation window=1.3 m/z; NCE=55; orbitrap resolution=50K; scan range=110-500 m/z and AGC=1e5.

Data analysis

The acquired LC-MS/MS raw files, were processed using MaxQuant[74] with the integrated Andromeda search engine (v1.6.6.0 or v1.6.17.0). MS/MS spectra were quantified with reporter ion MS2 or MS3, and searched against Human Reviewed UniProt Fasta database (downloaded in 2019). Carbamidomethylation of cysteines was set as fixed modification, while methionine oxidation and N-terminal acetylation (protein) were set as variable modifications. Protein quantification requirements were set at 1 unique and razor peptide. In the identification tab, second peptides and match between runs were not selected. Other parameters in MaxQuant were set to default values.

The MaxQuant output file (proteinGroups.txt) was then processed with Perseus software[75] (v1.6.6.0 or v1.6.17.0). After uploading the matrix, the data was filtered to remove identifications from reverse database, identifications with modified peptide only, and common contaminants. Data were log_2-transformed, a valid value filter was applied and missing values for remaining proteins were imputed with standard settings. Data were then exported for further processing in MS Excel, where intensity values were normalized to bait protein levels for each sample, except for untagged control samples. Background binders were filtered if intensities were less than 4-fold enriched in any sample over an untagged cell line (TurboID-TTC5), or if average intensity in TurboID samples was less than 2-fold enriched over untagged control levels (TurboID-SCAPER). A two-tailed t-test was used to calculate p-values between sample groups. For TurboID-TTC5 versus K97A comparison, values from all conditions (DMSO, colchicine, nocodazole, two replicates each) were used for statistics, because SCAPER binding was independent of treatments. For TurboID-SCAPER, three replicates each of TurboID-SCAPER +/- CA4, and two replicates for ΔE620 + CA4 samples were analyzed. Data were plotted in GraphPad Prism.

Recombinant protein purification

WT and mutant 6xHis-TEV-Twin-Strep-tagged TTC5 ("Strep-TTC5") were purified from E. coli cells as described.[17] Briefly, BL21 DE3 cells were transformed with the respective pET28a plasmids and grown at 37°C in LB containing 50 μg/ml kanamycin. Induction was with 0.2 mM IPTG at an A600 of 0.6 at 16°C overnight. Bacterial lysate was prepared by sonication (Sonics Vibracell) in 25 ml cold lysis buffer [500 mM NaCl, 20 mM imidazole, 1 mM TCEP, 1x EDTA-free protease inhibitor cocktail (Roche), and 50 mM HEPES, pH7.4] per litre of cells. Clarified bacterial lysates from a 1 l culture were bound to a 0.5 ml column of Ni-NTA resin (Qiagen) by gravity flow. Columns were washed with ~40 column volumes of lysis buffer and eluted with 250 mM imidazole in lysis buffer. The eluate was then bound to a 200 μl column of Streptactin Sepharose (IBA 2-1201-010). After extensive washing with 500 mM NaCl, 1 mM TECP and 50 mM HEPES, pH 7.4, TTC5 protein was eluted with 400 μl washing buffer containing 50 mM biotin and dialyzed against dialysis buffer (500 mM NaCl, 25 mM HEPES, pH 7.4).

Recombinant N- or C-terminally FLAG-tagged SCAPER was purified from Expi293 cells. Briefly, 100 ml cells were transfected with pcDNA3 or pcDNA5 plasmids encoding SCAPER constructs using polyethyleneimine-Max (made in-house) and grown for 72 hours for protein expression. Cells were pelleted and lysed in 10 ml lysis buffer [50 mM HEPES pH7.4, 400 mM KAc, 2 mM $MgAc_2$, 0.01% digitonin, 1 mM DTT, 1x EDTA-free protease inhibitor cocktail (Roche)] using a dounce homogenizer. Cleared lysates were incubated with 250 μl anti-FLAG resin (Sigma) with rotation for 2 hours at 4°C. The resin was then transferred to a gravity flow column (BioRad) and washed with 80 column volumes of lysis buffer, and 20 column volumes of wash buffer (50 mM HEPES pH7.4, 400 mM KAc,

2 mM $MgAc_2$). Proteins were eluted in two column volumes of 0.2 mg/ml 3xFLAG peptide (Sigma) and dialyzed against 50 mM HEPES pH 7.4, 400 mM KAc, 2 mM $MgAc_2$.

Pull-down assays

For pull-downs of recombinant SCAPER by TTC5, proteins were mixed at 100 nM (SCAPER) or 150 nM (TTC5) final concentration in 400 µl reactions in IP buffer (50 mM HEPES pH7.4, 100 mM KAc, 5 mM $MgAc_2$, 1 mM DTT, 0.01% digitonin). Reactions were incubated rotating for 1 hour at 4°C, 5 µl of streptactin magnetic agarose beads (IBA 2-4090-010) were added and samples were incubated another 1 hour. Beads were washed five times with 400 µl IP buffer and transferred to a new tube with the last step. Proteins were eluted with sample buffer, separated by SDS-PAGE, and gels were stained with Coomassie brilliant blue.

In vitro transcription and translation

All in vitro transcription of tubulin constructs utilized PCR product as template and were carried out as described.[17] The 5' primer contained the SP6 promoter sequence and anneals to the CMV promoter of pCDNA3.1. The 3' primers anneal at codon 54-60 or 84-90 of tubulin and contain extra sequence encoding MKLV to generate 64-mer or 94-mer nascent chains, respectively. Transcription reactions were carried out with SP6 polymerase (NEB) and RNasin ribonuclease inhibitor (Promega) for 1 hour at 37°C. Transcription reactions were directly used for in vitro translation in a homemade rabbit reticulocyte lysate (RRL)-based translation system as previously described,[85,89] optionally in the presence of ^{35}S-methionine. Recombinant Strep-TTC5 (100–250 nM) or FLAG-SCAPER (100–250 nM) proteins were included in the translation reactions as indicated. Translation reactions were at 32°C for 15 minutes, or 30 minutes for large-scale reactions for structural analysis. For analysis of total translation level of nascent chains, a 1 µl aliquot of the translation reaction was mixed with protein sample buffer and analyzed by SDS-PAGE gel electrophoresis and autoradiography.

For analysis of ribosome nascent chains (RNCs), translation products were pulled down via the Twin-Strep-tag on TTC5 using Streptactin Sepharose (IBA 2-1201-010) for 2 hours at 4°C. Beads were washed four times with PSB (50 mM HEPES pH7.4, 100 mM KAc, 2 mM $MgAc_2$) and eluted with 50 mM biotin in PSB for 30 minutes on ice. Elutions were analyzed by SDS-PAGE followed by SYPRO Ruby staining (Thermo Fisher), western blotting, or autoradiography. Alternatively, translation reactions were separated on linear 10–50% sucrose gradients (55,000 rpm, 20 min) and analyzed as above.

Structural analysis of TTC5-SCAPER-ribosomes

Cryo-EM grid preparation and data collection

Affinity purified ribosomes at a concentration of ~ 65 nM (A_{260} of 3.2) were vitrified on UltrAuFoil R1.2/1.3 300-mesh grids (Quantifoil), coated with graphene oxide (GO). For GO coating, gold grids were washed with deionized water, dried and subsequently glow-discharged for 5 min with an Edwards glowdischarger at 0.1 torr and 30 mA. 3 µl of a 0.2 mg/ml GO suspension in deionized water (Sigma) was pipetted onto the glow-discharged grids and incubated for 1 min. Next the GO solution was blotted away, and the grids were washed 3x by dipping into 20 µl deionized water drops followed by blotting (washed twice the top-side and once the bottom-side). 3 µl sample was pipetted onto the grids, blotted for 5.5 sec, -15 blot force, 0 sec wait at 100% humidity, 4°C, Whatman 595 blotting paper, with a Vitrobot Mark IV and plunge frozen into liquid ethane. Grids were stored in liquid nitrogen until data-collection. The dataset was collected with a Gatan K3 camera on a Titan Krios4 microscope at eBIC (Diamond) in super-resolution counting mode and binning 2, using EPU software in faster acquisition mode (AFIS), yielding 20932 micrographs (105000x magnification, pixel size= 0.829 Å, total dose 44.7 $e^-/Å^2$, 44 frames, resulting in 1 $e^-/Å^2$ dose/frame). Refer to Table S1 for data collection statistics.

Cryo-EM data processing

Datasets were processed with RELION 4.[76] Raw movies were corrected with MotionCor (5x5 patches), followed by CTF correction using CTFFIND-4.1. Particles were picked using low-pass filtered 80S ribosomes as a 3D reference, resulting in 1227269 initial particles, which were used for initial 2D classification. Good 2D classes (696074 particles) were selected and subjected to 3D classification without alignment using data to 8.29 Å, which resulted in 559080 high-resolution 80S particles. Particles were then re-extracted at 1.32 Å/pixel and 3D refined, yielding an overall resolution of 2.89 Å. To select TTC5 and SCAPER bound 80S, we performed focused classification with signal subtraction (FCwSS) around TTC5 and SCAPER without alignment, resulting in 22610 particles. These particles were then extracted at full pixel size (0.829 Å) and we performed Bayesian polishing and CTF refinement [(anisotropic) magnification estimation followed by CTF parameter fitting (fit defocus, astigmatism and B-factor per particle)], resulting in a map with an overall resolution of 2.89 Å. These particles were subjected to three different FCwSS. First, we performed a FCwSS around the expansion segment contacting SCAPER, yielding two classes with density corresponding to the expansion segment, resulting in two maps with an overall resolution of 3.10 Å (9158 particles) and 3.17 Å (7370 particles), respectively. Second, we performed a FCwSS around the P-site tRNA, resulting in a map with 3.24 Å resolution (5424 particles). Third, we did a FCwSS around SCAPER to remove some non-SCAPER containing particles, which resulted in a map with an overall resolution of 2.95 Å (18949 particles). The 40S subunit was in several rotation states, so we subtracted the 40S and focused on the 60S. This step resulted in an overall resolution of 2.84 Å after 3D refinement for the 60S subunit bound by TTC5 and SCAPER.

Model building, refinement and validation

The molecular model from PDB 6T59 (60S bound to TTC5 and tubulin nascent chain) was split into two groups, which were individually docked into the 2.8 Å post-processed map using UCSF Chimera (version 1.15).[79] TTC5 and the nascent chain from PDB 6T59 were deleted and replaced by an AlphaFold2 model[38,39,90] of TTC5 bound to the β-tubulin nascent chain. The SCAPER model was

also derived from AlphaFold2. Similar to the two groups of PDB 6T59, TTC5–β-tubulin nascent chain and SCAPER were individually docked in Chimera. Subsequently, all chains were manually adjusted into the original, or suitably blurred maps (B factors of 60 to 100) using Coot (version 0.9.6, Marina Bay).[77] TTC5–β-tubulin nascent chain and SCAPER were merged into group 2 of PDB 6T59. In Phenix (version 1.20-4459-000),[78] the 2 groups were first combined using *iotbx.pdb.join_fragment_files* and then *phenix.real_space_refine* was used to perform real space refinement of the resulting model with default settings and the following additions: *phenix.elbow* was used to automatically obtain restraints for all non-standard RNA bases and ligands; nonbonded weight of 1000 was used; rotamer outliers were fixed using the Fit option 'outliers_or_poormap' and the Target was set to 'fix_outliers'; and finally 112 processors were used to speed up the calculations. Refer to Table S1 for processing, refinement and model statistics.

Molecular graphics

Map and model figures were generated using UCSF Chimera (version 1.15),[79] UCSF Chimera X (version 1.3)[80] and PyMOL (Molecular Graphics System, version 2.4, Schrödinger, LLC). 2D class averages were generated in RELION 4.0 and the FSC curve was plotted using GraphPad Prism.

Structural modelling

Structure predictions were performed with AlphaFold2 through a local installation of Colabfold 1.2.0,[81] using MMseqs2[91] for homology searches and AlphaFold2[38] or AlphaFold2 multimer[39] for the predictions of single or multiple chains, respectively.

QUANTIFICATION AND STATISTICAL ANALYSIS

Quantification

Quantification of mRNA levels and mitosis defects, as well as proteomics analysis are described in the relevant methods sections.

Statistical analysis

All data analysis and statistical testing were performed in GraphPad Prism or Microsoft Excel. For mRNA quantification by RT-qPCR, statistical analysis was performed using an unpaired, two-tailed Student's t-test to compare two samples as indicated, typically with the WT cell line as a reference. A one-sample t-test was performed to compare a population to a hypothetical value where indicated. All bar graphs show means. Where 3 or more replicates were analyzed, error bars denote standard deviation (SD). The exact number of independent experiments and replicate samples within a particular experiment is given in the figure legends for each panel. Note that in many cases, additional independent replicates were performed as parts of other experiments shown in the paper (e.g., the many examples of tubulin mRNA decay in wild type, knockout, and rescue cell lines). For this reason, confidence in key results is in fact substantially greater than indicated solely by the *p-value*s indicated for an individual experiment.

To ensure that a sufficient number of independent replicates were performed in the autoregulation assays, a formal post-hoc power analysis was performed. The magnitude and precision of our mRNA degradation measurements in wild type cells was determined by combining all fifteen fully independent measurements shown in this study. The normalized mRNA level after 3 h of CA4 treatment (to trigger autoregulation) falls to 0.36 ± 0.11 for TUBA1B and 0.35 ± 0.09 for TUBB (n=15). A power analysis was performed to determine how many measurements are needed to have 95% confidence (for both type I and type II errors) for a particular difference from these normal values seen in wild type cells. This showed that a single measurement is sufficient to draw a confident conclusion for any effect where the mRNA level is 0.75 and above for TUBA1B and 0.66 and above for TUBB. To ensure reproducibility, we performed at least two independent experiments, which allows 95% confidence for mRNA levels of 0.64 and above for TUBA1 and 0.57 and above for TUBB. Because our conclusions do not depend on a finer level of discrimination than this, a minimum N of 2 was used throughout.

For proteomics data analysis, an unpaired, two-tailed Student's t-test was applied to calculate *p-value*s for comparisons, and the number of samples for each condition is stated in the figure legend. We have chosen not to display any significance cut-off, but have validated the relevance of the highlighted key interactors. The data to generate the plots is available in Tables S2 and S3.

For analysis of mitosis defects, individual event frequencies were calculated for each replicate, and then means were calculated across three replicate experiments. Statistical significance of the phenotypes was established by performing an unpaired, two-tailed Student's t-test.

All source data plotted in quantitative assays and statistical analysis with exact *p-value*s is available in Table S4, including additional RT-qPCR data for tubulin isoforms not shown in some figures. Significance is reported for *p-value*s < 0.05, with the following symbols $*p < 0.05$, $**p < 0.01$, $***p < 0.001$, ns: not significant ($p > 0.05$), as indicated in the figure legends.

Reproducibility

Reproducibility and reliability of the findings have been ensured in several ways. All biochemical in vitro experiments were reproduced on separate and fully independent occasions with comparable results to the examples shown in the figures. The cryo-EM structure was determined from a single sample, but similar densities were observed in an independently prepared sample. Key interactions have been validated by mutagenesis. Tubulin autoregulation assays were performed on at least two independent

occasions, with triplicate measurements in qPCR for every datapoint. For all key cell lines and constructs, we show three replicates. In cases where only two replicates are shown, the findings are reproduced elsewhere in the manuscript or validated by other means. For example, WT, TTC5-KO, and TTC5 rescue cells from Figure 1B (n = 2) were measured three more times in Figure S1E with an alternative tag on the TTC5 rescue constructs. Similarly, SCAPER siRNA knock-down phenotypes (Figure S3A) were further validated by establishing multiple independent KO cell lines in both HEK293 and HeLa T-REx cells. Live-cell microscopy assays for chromosome alignment and segregation errors show data from three independent experiments with at least 100 cells per cell line in total.

Molecular Cell, Volume *83*

Supplemental information

Mechanism of ribosome-associated

mRNA degradation during tubulin autoregulation

Markus Höpfler, Eva Absmeier, Sew-Yeu Peak-Chew, Evangelia Vartholomaiou, Lori A. Passmore, Ivana Gasic, and Ramanujan S. Hegde

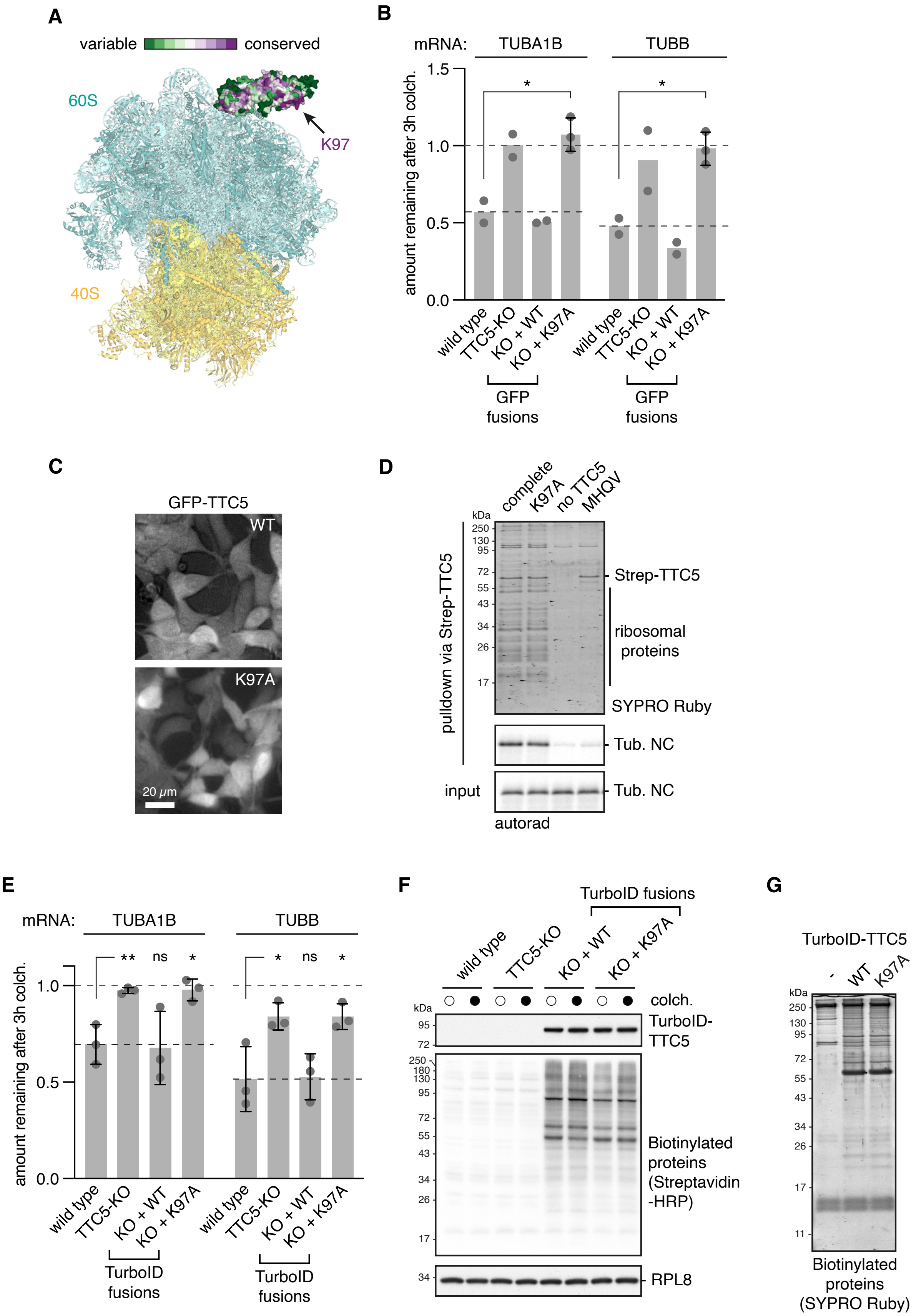
A
variable
conserved
60S
K97
40S
B
mRNA:
TUBA1B
TUBB
amount remaining after 3h colch.
1.5
1.0
0.5
0.0
*
*
wild type
TTC5-KO
KO + WT
KO + K97A
GFP fusions
C
GFP-TTC5
WT
K97A
20 μm
D
complete
K97A
no TTC5
MHQV
kDa
250
130
95
72
55
43
34
26
17
pulldown via Strep-TTC5
Strep-TTC5
ribosomal proteins
SYPRO Ruby
Tub. NC
input
Tub. NC
autorad
E
mRNA:
TUBA1B
TUBB
amount remaining after 3h colch.
1.0
0.5
0.0
**
ns
*
*
ns
*
wild type
TTC5-KO
KO + WT
KO + K97A
TurboID fusions
F
TurboID fusions
wild type
TTC5-KO
KO + WT
KO + K97A
colch.
kDa
95
72
TurboID-TTC5
250
180
130
95
72
55
43
34
26
17
Biotinylated proteins (Streptavidin -HRP)
34
RPL8
G
TurboID-TTC5
-
WT
K97A
kDa
250
130
95
72
55
43
34
26
17
11
Biotinylated proteins (SYPRO Ruby)

Figure S1. The TTC5 K97A mutation abolishes tubulin autoregulation, related to Fig. 1.

(A) Model of TTC5 bound to the ribosome containing a 64 amino acid β-tubulin nascent chain from a previously published structure [S1] (PDB: 6T59) with the 40S subunit docked for reference. ConSurf [S2] was used to display the residue conservation of TTC5 on the surface. The highly conserved surface patch containing the K97 residue is indicated. This patch is not involved in either ribosome binding or nascent tubulin recognition, leading us to a hypothesis in which it is a docking site for downstream factors. (B) Tubulin autoregulation assay as described in Fig. 1B for TUBB, with TUBA1B reproduced from Fig. 1B for comparison. The red dashed line indicates the starting tubulin mRNA level prior to colchicine, arbitrarily set to a value of 1. The black dashed lines indicate the fold change in wild type (WT) cells for each tubulin subunit. Both TUBA1B and TUBB mRNA were quantified in all experiments in the manuscript. Because the results were comparable for both mRNAs throughout, we display only one tubulin gene in most figures for brevity and clarity. TTC5-KO cells were complemented by stable inducible expression with either GFP-tagged wild type (WT) TTC5 or the K97A mutant. Data show the mean from 2 independent experiments, one of which contained 2 replicates for the TTC5 K97A cell line. Error bars denote standard deviation (SD). Single asterisks denote significant stabilization of tubulin mRNA in KO + K97A cells compared to WT ($p<0.05$) (C) The complemented GFP-tagged cell lines from panel B were imaged using a wide-field microscope (Life Technologies EVOS FL) to verify that expression and localization were similar for both constructs. The same microscope settings were used for both images. (D) Reconstitution of TTC5 recruitment to tubulin ribosome nascent chains (RNCs). 94-residue β-tubulin (TUBB) nascent chains were produced in rabbit reticulocyte lysates in the presence of recombinant WT or K97A mutant Twin-Strep-tagged TTC5 (Strep-TTC5) as indicated. "MHQV" indicates a β-tubulin construct in which its TTC5-interacting MREI motif has been mutated. TTC5-associated proteins were enriched via its Strep tag, separated by SDS-PAGE and visualized by SYPRO Ruby staining for total protein (top) or autoradiography for the β-tubulin nascent chain (Tub. NC, bottom). (E) Autoregulation assay with the indicated WT, TTC5-KO, or TurboID-TTC5 rescue cell lines. Data show the mean ± SD from 2 independent experiments, one of which contained 2 replicates. Single and double asterisks indicate $p<0.05$ and $p<0.01$, respectively; "ns" indicates not significant. (F) Western blot analysis of cell lines used in panel E. Cells were pre-treated with 10 μM colchicine (colch.) for 30 minutes as indicated by filled circles, and 50 μM biotin was added for 2.5 h (all samples). (G) Biotinylated proteins enriched from parental, TurboID-TTC5 WT or K97A cell lines as detected by SYPRO Ruby staining. Samples prepared in the same way were used for the mass spectrometry experiment in Fig. 1C. 50 μM biotin was added 2.5h prior to harvesting of cells. To avoid over-expression of TurboID-TTC5 constructs in biotinylation experiments, we exploited the basal leaky expression from the tetracycline-regulated CMV promoter seen in the absence of added doxycycline.

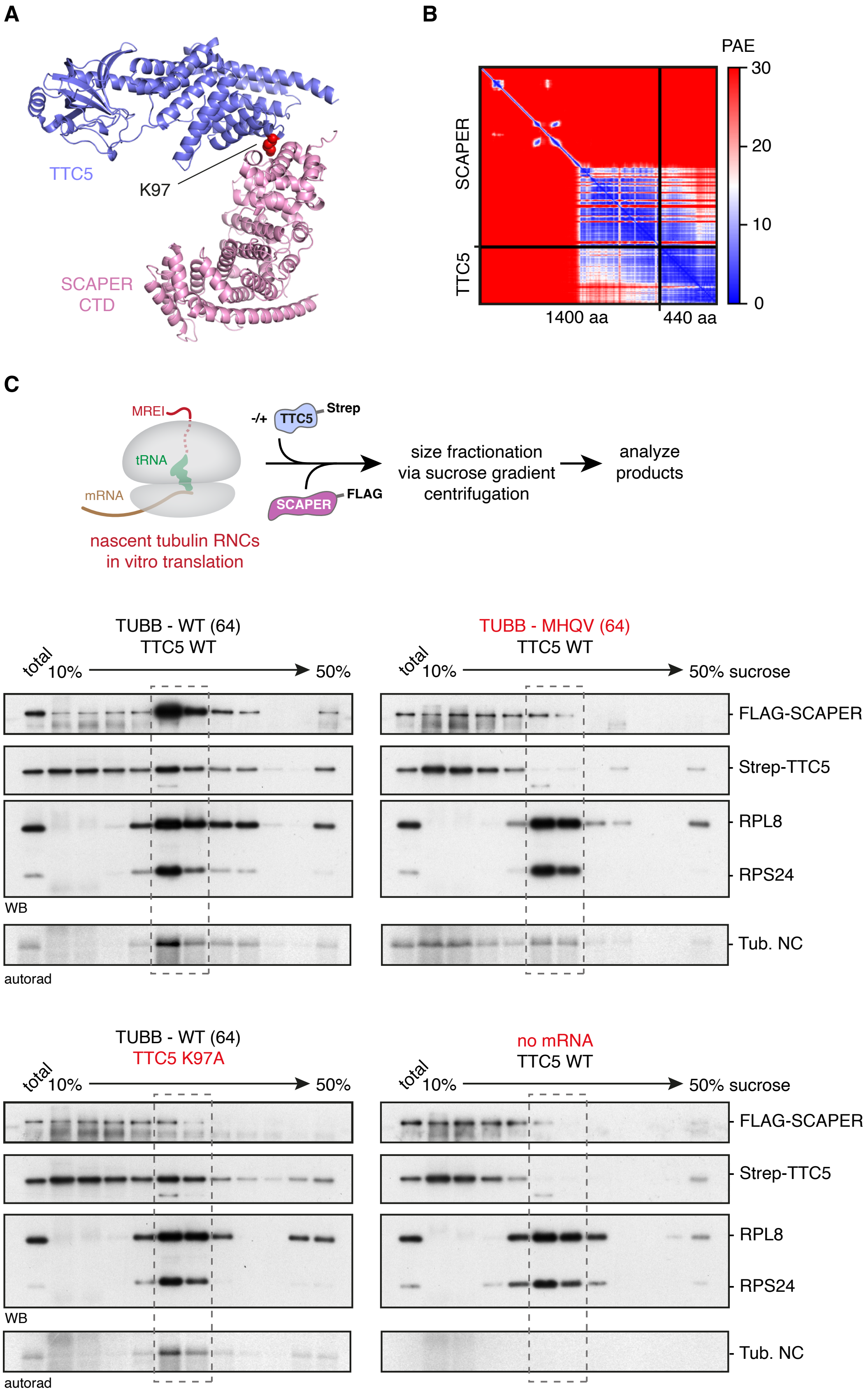
A
TTC5
K97
SCAPER
CTD
B
PAE
30
20
10
0
SCAPER
TTC5
1400 aa
440 aa
C
MREI
tRNA
mRNA
nascent tubulin RNCs
in vitro translation
-/+
TTC5
Strep
SCAPER
FLAG
size fractionation
via sucrose gradient
centrifugation
analyze
products
TUBB - WT (64)
TTC5 WT
total
10%
50%
TUBB - MHQV (64)
TTC5 WT
50% sucrose
FLAG-SCAPER
Strep-TTC5
RPL8
RPS24
WB
Tub. NC
autorad
TUBB - WT (64)
TTC5 K97A
no mRNA
TTC5 WT

Figure S2. TTC5 recruits SCAPER to tubulin ribosome nascent chain complexes, related to Fig. 2. (A) Top-ranked AlphaFold2 (AF2) multimer prediction of the interaction between TTC5 and SCAPER. The SCAPER C-terminal domain (CTD, residues 857–1400) is shown, with the rest of the molecule omitted for clarity. The TTC5 K97 residue is highlighted in red spheres. (B) Predicted Aligned Error (PAE) matrix of the TTC5-SCAPER AF2 multimer prediction shown in panel A. Lower PAE values (blue) indicate higher confidence in the relative positions and orientation between residue pairs in the model. Note that a strong signal is observed between TTC5 and the CTD of SCAPER. (C) 64-residue β-tubulin (TUBB) nascent chain complexes were produced by in vitro translation in the presence of SCAPER and TTC5 as in Fig. 2C. Reactions were then centrifuged through 10–50% sucrose gradients and fractions were analysed by SDS-PAGE, western blotting (WB), and autoradiography (autorad). An aliquot of the total reaction is analyzed in the first lane of each gradient. The peak ribosome-containing fractions, predominantly monosomes, are indicated by dashed boxes. Both the WT β-tubulin sequence and WT TTC5 are required for efficient recruitment of SCAPER to RNCs.

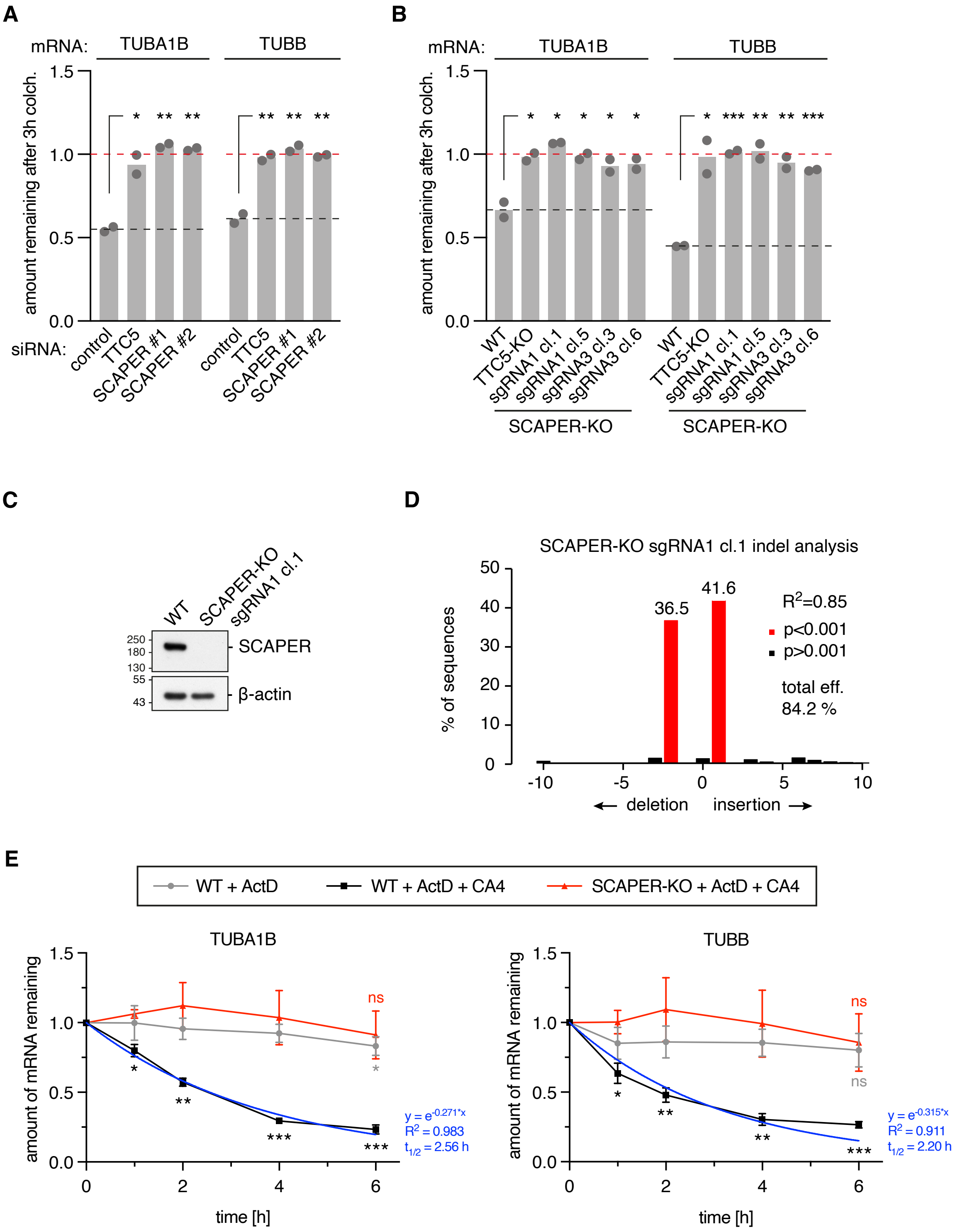

A
mRNA:
TUBA1B
TUBB
amount remaining after 3h colch.
siRNA:
control
TTC5
SCAPER #1
SCAPER #2
B
WT
TTC5-KO
sgRNA1 cl.1
sgRNA1 cl.5
sgRNA3 cl.3
sgRNA3 cl.6
SCAPER-KO
C
SCAPER-KO sgRNA1 cl.1
SCAPER
β-actin
D
SCAPER-KO sgRNA1 cl.1 indel analysis
% of sequences
36.5
41.6
R2=0.85
p<0.001
p>0.001
total eff. 84.2 %
deletion
insertion
E
WT + ActD
WT + ActD + CA4
SCAPER-KO + ActD + CA4
TUBA1B
TUBB
amount of mRNA remaining
time [h]
ns
y = e-0.271*x
R2 = 0.983
t1/2 = 2.56 h
y = e-0.315*x
R2 = 0.911
t1/2 = 2.20 h

Figure S3. SCAPER is required for tubulin autoregulation, related to Fig. 2.
(A) Tubulin mRNA levels were determined in the qPCR-based autoregulation assay for cells transfected with the indicated siRNAs for 72h. Samples from colchicine-treated cells (10 μM for 3h) were normalized to untreated control samples, arbitrarily set to a value of 1 (red dashed line). The black dashed lines indicate the fold change in wild type (WT) cells for each set of samples. Data show the mean from 2 independent experiments. (B) Autoregulation assays were performed using the indicated HEK293 T-REx cell lines or four different SCAPER KO clones (cl.) obtained from two different sgRNAs. Data show the mean from 2 independent experiments. Single, double, and triple asterisks indicate $p<0.05$, $p<0.01$, and $p<0.001$, respectively. (C) Whole cell lysates from HEK T-REx WT and SCAPER KO (sgRNA1 cl.1) were analyzed by SDS-PAGE and western blotting using anti-SCAPER and anti-β-actin antibodies. (D) Indel analysis for SCAPER KO sgRNA1 cl.1 using genomic DNA sequencing followed by TIDE analysis [S3]. This clone shows a 1-nucleotide insertion and a 2-nucleotide deletion for the two alleles. sgRNA1 targets exon 5 around codon 127. The same clone was used throughout the rest of the study and to generate rescue cell lines. (E) Quantification of mRNA decay rates in HEK T-REx WT and SCAPER KO cells after transcription shut-down with actinomycin D (ActD) for α-tubulin (TUBA1B, top) and β-tubulin (TUBB, bottom). Cells were treated with 5 μg/ml ActD or 100 nM combretastatin A4 (CA4) from $t = 0$ as indicated. TUBA1B and TUBB mRNAs were quantified by RT-qPCR and normalized to GAPDH (a transcript that was stable for the observed period) and the $t = 0$ value. Statistical analysis was performed using a one-sample t-test. Values significantly different from 1 ($t = 0$ value) are indicated by single, double, and triple asterisks ($p<0.05$, $p<0.01$, and $p<0.001$, respectively; "ns" indicates not significant). For the WT + ActD + CA4 samples, an exponential decay curve was fitted to the data (blue curve and parameters). Data show mean ± SD from 3 replicates.

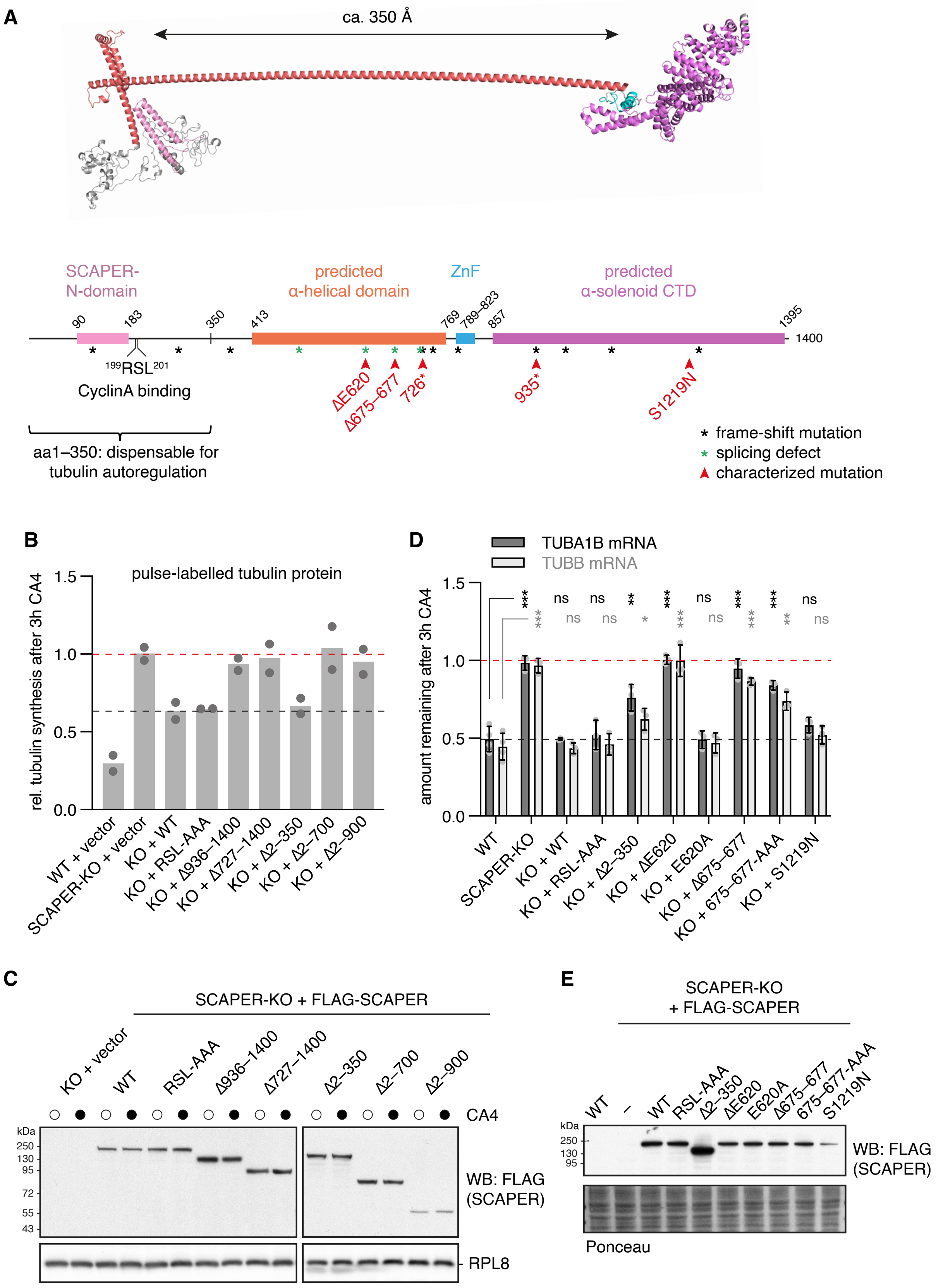

A
ca. 350 Å
SCAPER-
N-domain
predicted
α-helical domain
ZnF
predicted
α-solenoid CTD
90
183
350
413
769
789–823
857
1395
1400
199RSL201
CyclinA binding
ΔE620
Δ675–677
726*
935*
S1219N
aa1–350: dispensable for
tubulin autoregulation
frame-shift mutation
splicing defect
characterized mutation
B
pulse-labelled tubulin protein
rel. tubulin synthesis after 3h CA4
WT + vector
SCAPER-KO + vector
KO + WT
KO + RSL-AAA
KO + Δ936–1400
KO + Δ727–1400
KO + Δ2–350
KO + Δ2–700
KO + Δ2–900
D
TUBA1B mRNA
TUBB mRNA
amount remaining after 3h CA4
WT
SCAPER-KO
KO + WT
KO + RSL-AAA
KO + Δ2–350
KO + ΔE620
KO + E620A
KO + Δ675–677
KO + 675–677-AAA
KO + S1219N
C
SCAPER-KO + FLAG-SCAPER
KO + vector
WT
RSL-AAA
Δ936–1400
Δ727–1400
Δ2–350
Δ2–700
Δ2–900
CA4
kDa
WB: FLAG
(SCAPER)
RPL8
E
SCAPER-KO
+ FLAG-SCAPER
WT
–
WT
RSL-AAA
Δ2–350
ΔE620
E620A
Δ675–677
675–677-AAA
S1219N
kDa
WB: FLAG
(SCAPER)
Ponceau

Figure S4. The SCAPER α-helical and C-terminal domains are critical for tubulin autoregulation, related to Fig. 2.

(A) Top: Structural model of SCAPER predicted by trRosetta [S4]. The displayed model was stitched from two overlapping predictions. Subdomains are color-coded as in the schematic below. Bottom: Schematic of the SCAPER domain architecture with annotated and predicted features indicated (drawn to scale). Pathologic mutations [S5–S7] characterized in autoregulation assays in this study are indicated by red arrow heads. Positions of pathologic frameshift and splice site mutations are indicated by black and green asterisks, respectively. RSL: cyclin A binding motif (Arg^{199}-Ser^{200}-Leu^{201}); ZnF: Zinc finger; CTD: Carboxy-terminal domain. (B) Tubulin autoregulation was tested by pulse-labelling of newly synthesized proteins with ^{35}S-methionine. Indicated cell lines were transiently transfected with FLAG-SCAPER encoding plasmids. Plotted is the ratio of ^{35}S-labelled tubulins from cells after 3h combretastatin A4 (CA4) treatment versus untreated control conditions as quantified by autoradiography. RSL-AAA: mutation of the cyclin A binding site (Arg^{199}-Ser^{200}-Leu^{201}) to alanines. Numbers refer to SCAPER amino acid (aa) positions. Mean and individual data points from two independent experiments are plotted. We note that pathologic alleles with truncations after residue 726 and 935 have been reported to cause disease phenotypes such as intellectual disability, a Bardet-Biedl syndrome-like illness, and male infertility [S6, S8]. (C) Total protein analysis by western blotting of cells used in panel B. (D) Autoregulation assay with WT, SCAPER-KO and the indicated stable rescue cell lines. Note that part of the data is reproduced from Fig. 2D for comparison. Data show the mean ± SD from 2 independent experiments, one of which contained 2 replicates. For statistical comparisons against the WT cell line for each tubulin isoform, single, double, and triple asterisks indicate $p<0.05$, $p<0.01$, and $p<0.001$, respectively; "ns" indicates not significant. (E) Western blot analysis of SCAPER expression levels of cell lines used in panel D and Fig. 2D. Note that the SCAPER-S1219N variant shows lower expression levels. This indicates that the mutation might destabilize the protein, a potential explanation for the pathologic phenotype it has in a heterozygous patient with the ΔE620 mutation on the second allele [S5]. Under overexpression conditions, SCAPER-S1219N is functional for autoregulation (see panel D).

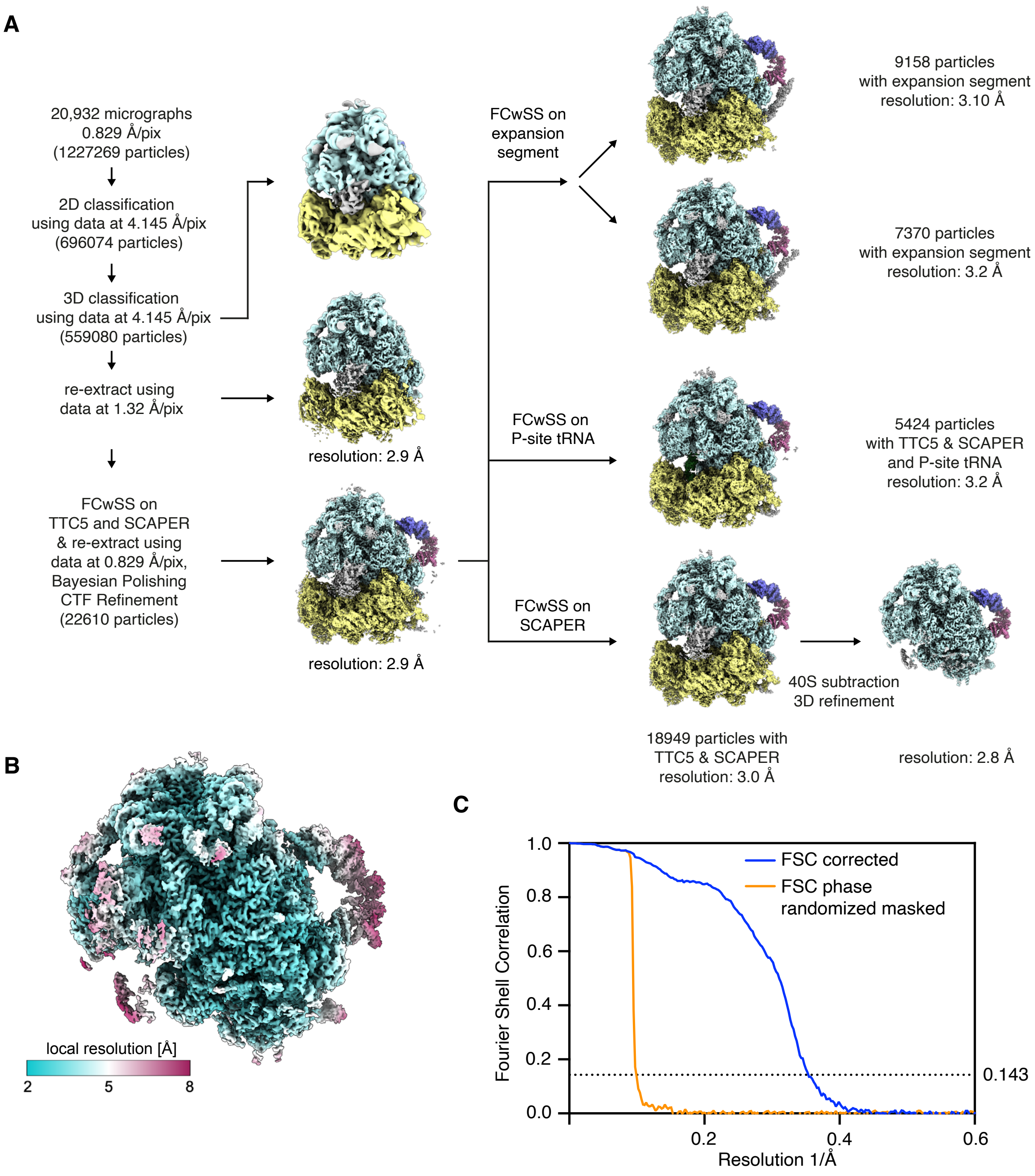

Figure S5. Cryo-EM analysis of SCAPER bound to TTC5 and 80S ribosomes, related to Fig. 3.
(A) RELION 4.0 classification and processing workflow for reconstruction of ribosomal particles from cryo-EM micrographs. FCwSS: Focussed classification with signal subtraction. (B) Map coloured according to the local resolution after final FCwSS around SCAPER and subtraction of the 40S subunit. (C) Gold-standard Fourier shell correlation (FSC) curve (blue) of the final 60S-TTC5-SCAPER map illustrating an overall resolution of 2.8 Å. The phase-randomized, masked FSC curve (orange) is also shown.

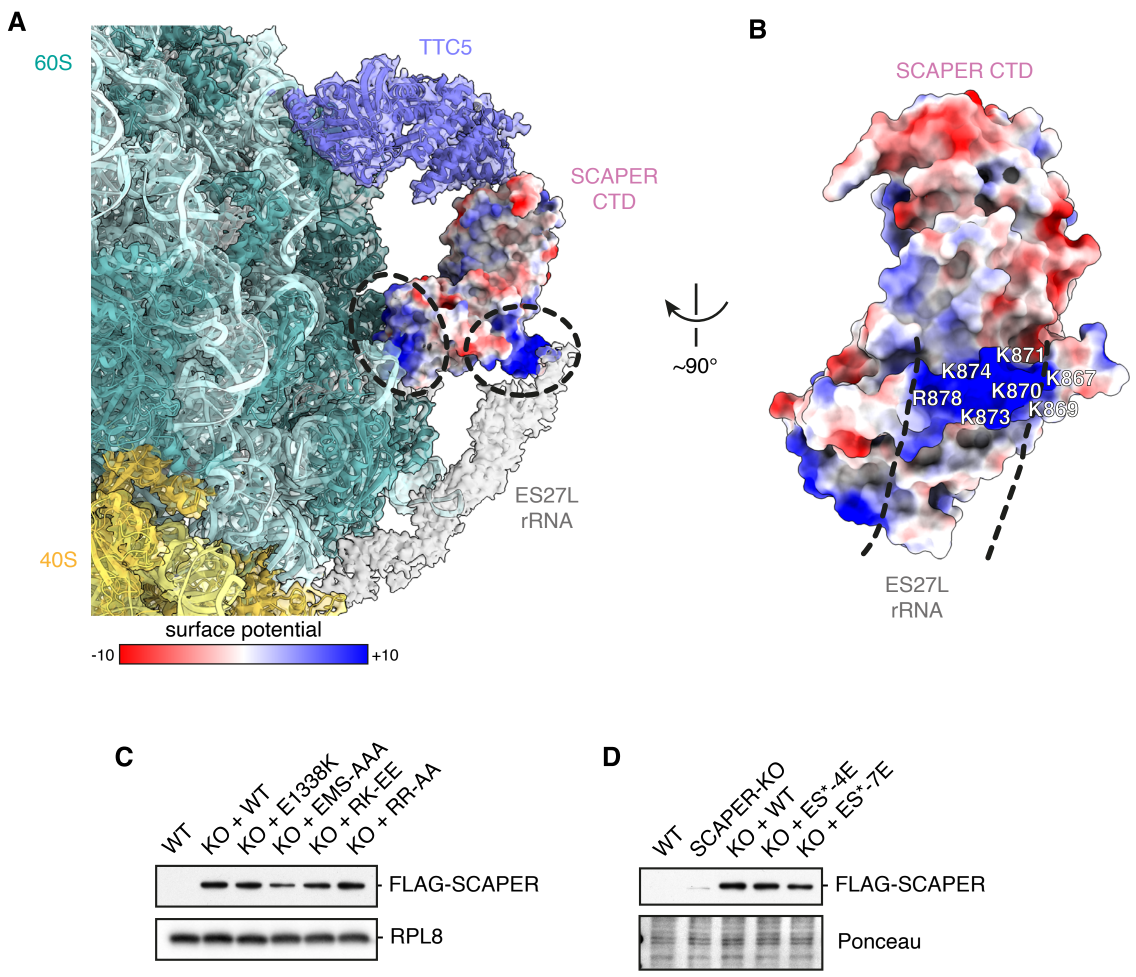

Figure S6. SCAPER contacts with 28S rRNA expansion segment ES27L, related to Fig. 3.
(A) View of the cryo-EM map with electrostatic surface potential [in kcal/(mol**e*)] displayed on the SCAPER CTD model. Note the two highly basic surface patches on SCAPER (dashed outlines) that contact the 60S body and ES27L rRNA. (B) Side-view of the SCAPER-CTD model from panel A with the path of the density for the rRNA expansion segment ES27L marked by dashed lines. Positively charged residues that were targeted by mutagenesis are indicated. (C, D) Western blot analysis of expression levels of FLAG-SCAPER constructs used in Fig. 3E and 3F, respectively. EMS-AAA: E1338A, M1339A, S1340A; RK-EE: R907E, K910E; RR-AA: R934A, R941A. SCAPER mutations targeting expansion segment contacts were as follows: ES*-4E: K867E, K870E, K873E and K874E; ES*-7E: as ES*-4E plus K869E, K871E and R878E.

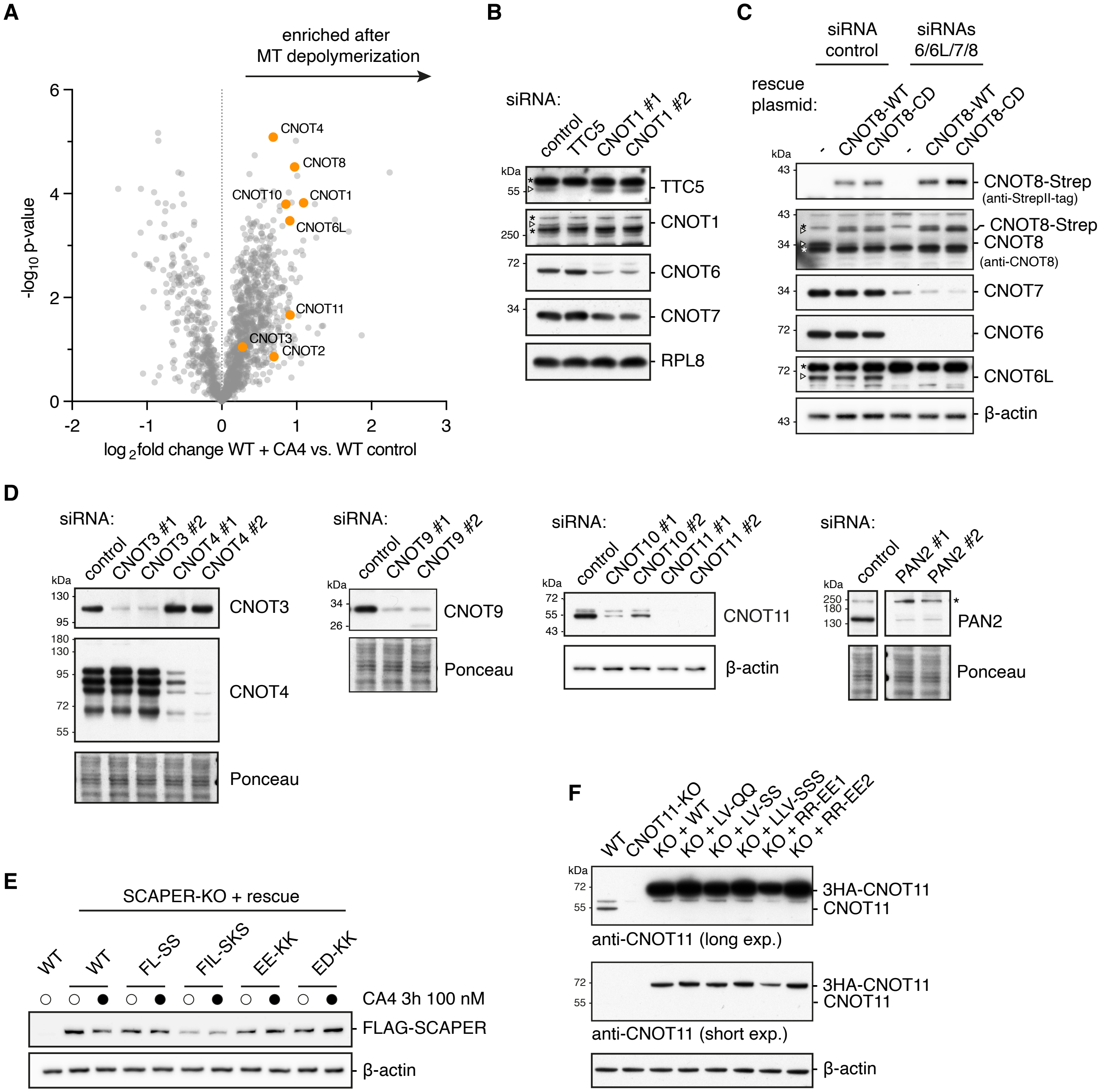
A
enriched after
MT depolymerization
-log10 p-value
log 2 fold change WT + CA4 vs. WT control
CNOT4
CNOT8
CNOT10
CNOT1
CNOT6L
CNOT11
CNOT3
CNOT2
B
siRNA:
control
TTC5
CNOT1 #1
CNOT1 #2
kDa
TTC5
CNOT1
CNOT6
CNOT7
RPL8
C
siRNA
control
siRNAs
6/6L/7/8
rescue
plasmid:
CNOT8-WT
CNOT8-CD
CNOT8-WT
CNOT8-CD
CNOT8-Strep
(anti-StrepII-tag)
CNOT8-Strep
CNOT8
(anti-CNOT8)
CNOT7
CNOT6
CNOT6L
β-actin
D
siRNA:
control
CNOT3 #1
CNOT3 #2
CNOT4 #1
CNOT4 #2
CNOT3
CNOT4
Ponceau
siRNA:
control
CNOT9 #1
CNOT9 #2
CNOT9
Ponceau
siRNA:
control
CNOT10 #1
CNOT10 #2
CNOT11 #1
CNOT11 #2
CNOT11
β-actin
siRNA:
control
PAN2 #1
PAN2 #2
PAN2
Ponceau
E
SCAPER-KO + rescue
WT
WT
FL-SS
FIL-SKS
EE-KK
ED-KK
CA4 3h 100 nM
FLAG-SCAPER
β-actin
F
WT
CNOT11-KO
KO + WT
KO + LV-QQ
KO + LV-SS
KO + LLV-SSS
KO + RR-EE1
KO + RR-EE2
3HA-CNOT11
CNOT11
anti-CNOT11 (long exp.)
3HA-CNOT11
CNOT11
anti-CNOT11 (short exp.)
β-actin

Figure S7. The CCR4-NOT complex is required for tubulin autoregulation, related to Fig. 4 and 5.
(A) Proximity labelling using HEK T-REx TurboID-SCAPER WT cells was performed in control conditions or after microtubule (MT) depolymerization with 100 nM Combretastatin A4 (CA4). Biotinylated proteins were enriched and analyzed by quantitative mass spectrometry. See also Supplementary Table S3. 3 replicates were analyzed for both conditions. (B) Western blot analysis of samples as used in Fig. 4D confirming knockdown of TTC5 and CNOT1. Note that depletion of the CNOT1 scaffolding subunit also leads to destabilization of other CCR4-NOT subunits (CNOT6, CNOT7). Specific bands are indicated by triangles for TTC5 and CNOT1; non-specific bands are indicated by asterisks. (C) Western blot analysis of samples used in Fig. 4E. Signals from blots with an antibody against endogenous CNOT8 (second panel) show both CNOT8 depletion after knockdown and expression of siRNA-resistant rescue constructs CNOT8-WT and CNOT8-CD. Specific bands are indicated by triangles for CNOT8 and CNOT6L; non-specific bands are indicated by asterisks. (D) Western blot analysis of knockdown efficiency for selected samples used in Fig. 5A. Although we did not have access to a functional CNOT10 antibody, we note that CNOT10-KD leads to destabilization of CNOT11, corroborating that they might act as a functional unit. (E) Western blot analysis of expression levels of FLAG-SCAPER constructs used in Fig. 5D. FL-SS: F628S, L632S; FIL-SKS: F628S, I629K, L632S; EE-KK: E618K, E625K; ED-KK: E633K, D640K. (F) Western blot analysis of expression levels of CNOT11 and rescue constructs used in Fig. 5E. Membranes were probed with an antibody raised against a peptide of endogenous CNOT11. The epitope was not affected by the indicated mutations. LV-QQ: L405Q, V454Q; LV-SS: L405S, V454S; LLV-SSS: L405S, L451S, V454S; RR-EE1: R447E, R450E; RR-EE2: R461E, R485E.

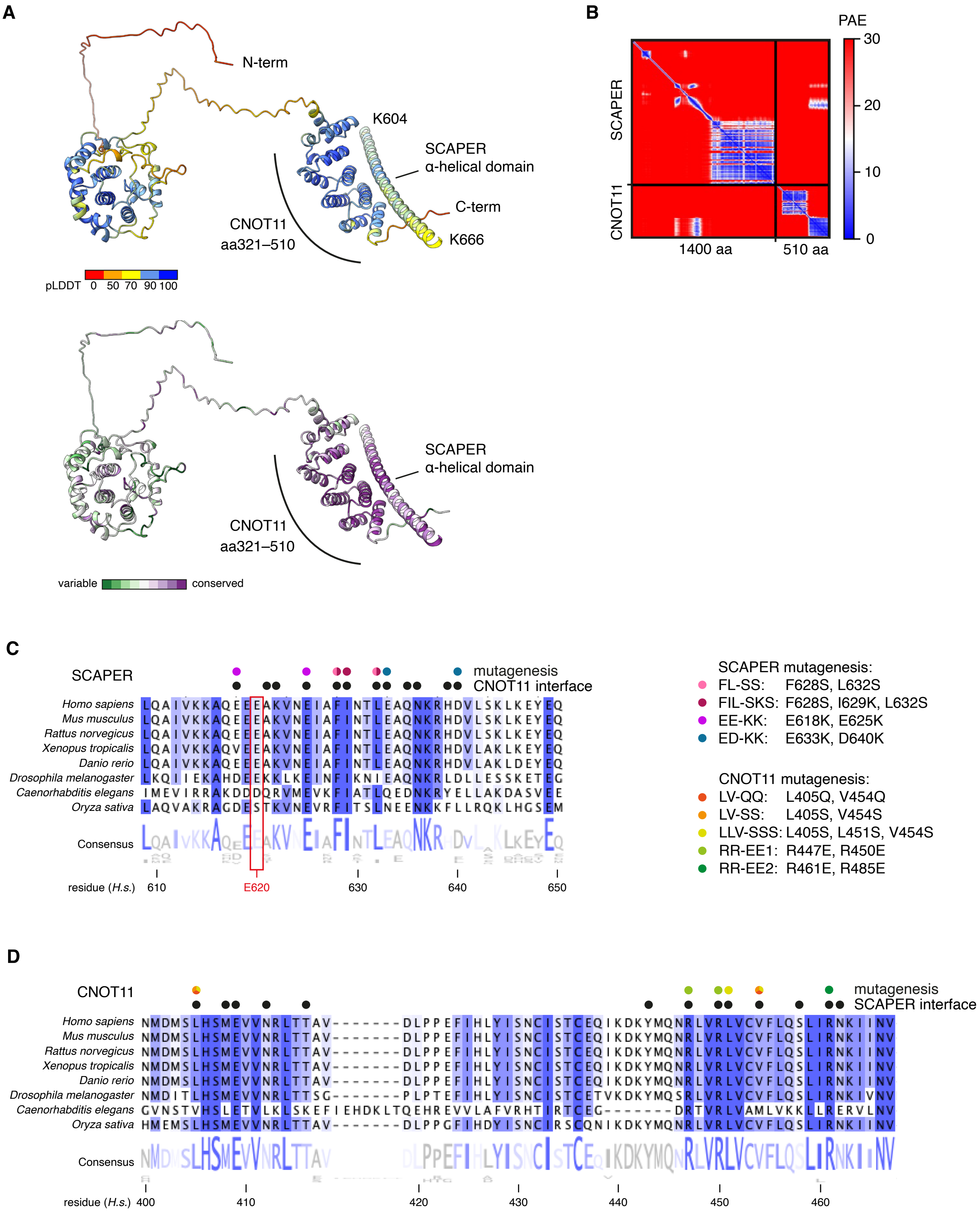

A
N-term
K604
SCAPER
α-helical domain
C-term
K666
CNOT11
aa321–510
pLDDT 0 50 70 90 100
SCAPER
α-helical domain
CNOT11
aa321–510
variable
conserved
B
PAE
30
20
10
0
SCAPER
CNOT11
1400 aa
510 aa
C
SCAPER
mutagenesis
CNOT11 interface
Homo sapiens LQAIVKKAQEEEAKVNEIAFINTLEAQNKRHDVLSKLKEYEQ
Mus musculus LQAIVKKAQEEEAKVNEIAFINTLEAQNKRHDVLSKLKEYEQ
Rattus norvegicus LQAIVKKAQEEEAKVNEIAFINTLEAQNKRHDVLSKLKEYEQ
Xenopus tropicalis LQAIVKKAQVEEAKVNEIAFINTLEAQNKRHDVLAKLKEYEQ
Danio rerio LQAIVKKAQEEEAKVNEIAFINTLEAQNKRHDVLAKLDEYEQ
Drosophila melanogaster LKQIIEKAHDEEKKLKEINFIKNIEAQNKRLDLLESSKETEG
Caenorhabditis elegans IMEVIRRAKDDDQRVMEVKFIATLQEDNKRYELLAKDASVEE
Oryza sativa LAQVAKRAGDESTKVNEVRFITSLNEENKKFLLRQKLHGSEM
Consensus
residue (H.s.) 610 E620 630 640 650
SCAPER mutagenesis:
FL-SS: F628S, L632S
FIL-SKS: F628S, I629K, L632S
EE-KK: E618K, E625K
ED-KK: E633K, D640K
CNOT11 mutagenesis:
LV-QQ: L405Q, V454Q
LV-SS: L405S, V454S
LLV-SSS: L405S, L451S, V454S
RR-EE1: R447E, R450E
RR-EE2: R461E, R485E
D
CNOT11
mutagenesis
SCAPER interface
Homo sapiens NMDMSLHSMEVVNRLTTAV-------DLPPEFIHLYISNCISTCEQIKDKYMQNRLVRLVCVFLQSLIRNKIINV
Mus musculus NMDMSLHSMEVVNRLTTAV-------DLPPEFIHLYISNCISTCEQIKDKYMQNRLVRLVCVFLQSLIRNKIINV
Rattus norvegicus NMDMSLHSMEVVNRLTTAV-------DLPPEFIHLYISNCISTCEQIKDKYMQNRLVRLVCVFLQSLIRNKIINV
Xenopus tropicalis NMDMSLHSMEVVNRLTTAV-------DLPPEFIHLYISNCISTCEQIKDKYMQNRLVRLVCVFLQSLIRNKIINV
Danio rerio NMDMSLHSMEVVNRLTTAV-------DLPPEFIHLYISNCISTCEQIKDKYMQNRLVRLVCVFLQSLIRNKIINV
Drosophila melanogaster NMDITLHSMEVVNRLTTSG-------PLPTEFIHLYISNCISTCETVKDKYMQSRLVRLVCVFLQSLIRNKIVNV
Caenorhabditis elegans GVNSTVHSLETVLKLSKEFIEHDKLTQEHREVVLAFVRHTIRTCEG-------DRTVRLVAMLVKKLLRERVLNV
Oryza sativa HMEMSLHSMEVVNRLTTAV-------DLPPGFIHDYISNCIRSCQNIKDKYMQNRLVRLVCVFLQSLIRNKIINV
Consensus
residue (H.s.) 400 410 420 430 440 450 460

Figure S8. Structural prediction of a putative SCAPER-CNOT11 complex, related to Fig. 5.
(A) Overview of an AF2 multimer predicted complex between CNOT11 and SCAPER coloured by predicted local distance difference test score (pLDDT, top, dark blue indicates high confidence), or per residue conservation (bottom). For SCAPER, only residues 604–666 of the α-helical domain are displayed for clarity. (B) PAE matrix of the model displayed in panel A. (C) Multiple sequence alignment of SCAPER homologs across the indicated species generated using Clustal Omega and displayed using Jalview software [S9, S10]. Highly conserved residues are shaded in blue. Residues in close proximity to CNOT11 in the AF2 predicted model are marked with black dots, and residues targeted in mutagenesis experiments are marked with color-coded dots. The E620 residue is highlighted by a red box. (D) Multiple sequence alignment of CNOT11 homologs generated as in panel C. Residues in close proximity to SCAPER in the AF2 model are marked by black dots, and residues targeted by mutagenesis are shown with coloured dots. Residue R485 is not shown in this alignment.

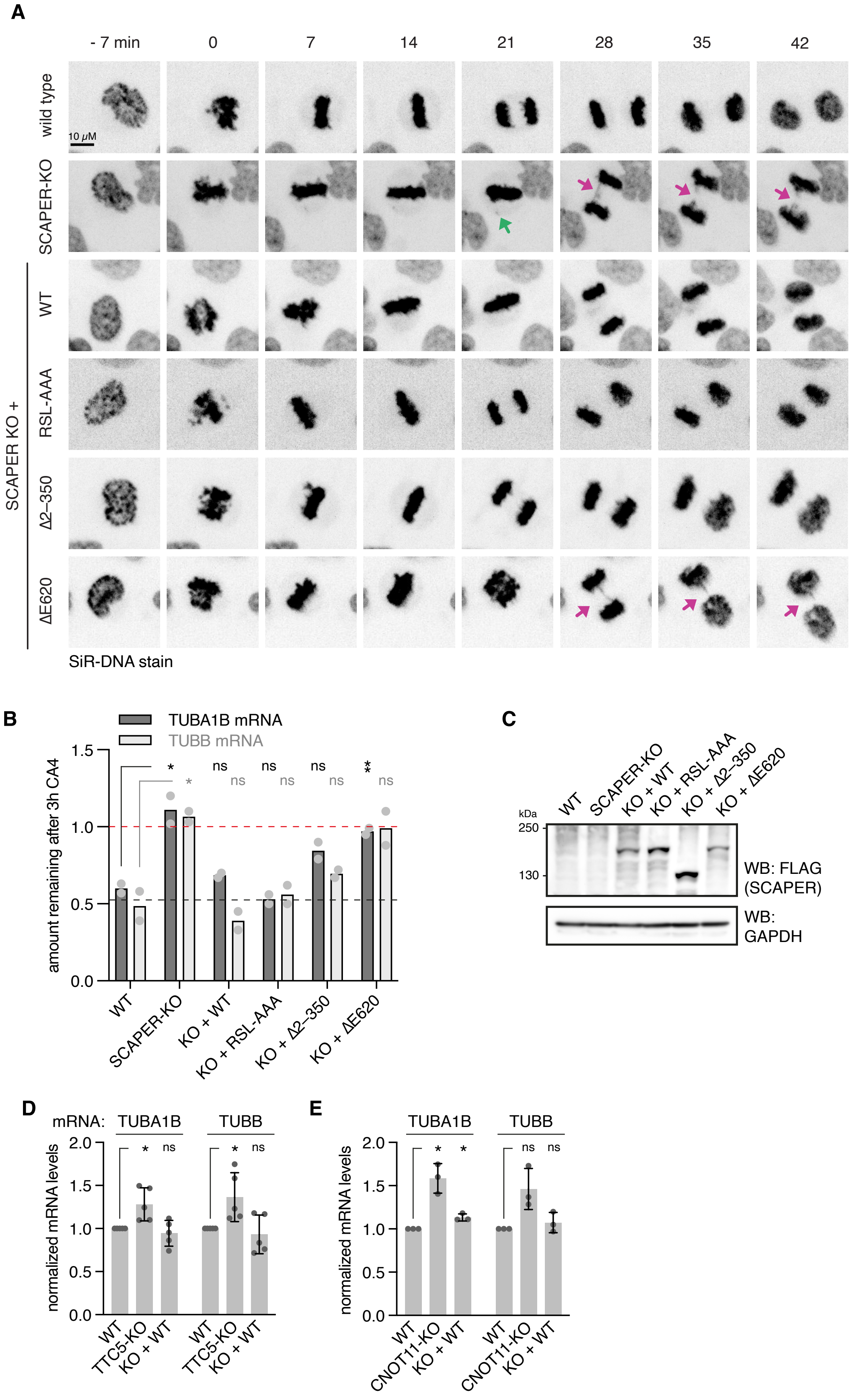
A
- 7 min
0
7
14
21
28
35
42
wild type
10 μM
SCAPER-KO
SCAPER KO +
WT
RSL-AAA
Δ2–350
ΔE620
SiR-DNA stain
B
TUBA1B mRNA
TUBB mRNA
amount remaining after 3h CA4
WT
SCAPER-KO
KO + WT
KO + RSL-AAA
KO + Δ2–350
KO + ΔE620
C
kDa
250
130
WT
SCAPER-KO
KO + WT
KO + RSL-AAA
KO + Δ2–350
KO + ΔE620
WB: FLAG (SCAPER)
WB: GAPDH
D
mRNA:
TUBA1B
TUBB
normalized mRNA levels
WT
TTC5-KO
KO + WT
E
TUBA1B
TUBB
normalized mRNA levels
WT
CNOT11-KO
KO + WT

Figure S9. SCAPER is required for faithful mitosis, related to Fig. 6.
(A) Examples from time-lapse imaging of HeLa T-REx cells of the indicated genotype going through mitosis. DNA was visualized using SiR-DNA stain and maximum intensity projections are shown. Frames were aligned to nuclear envelope breakdown (t = 0). Misaligned chromosomes and segregation errors are highlighted by green and magenta arrows, respectively. (B) Autoregulation assay for HeLa cell lines used in panel A and Fig. 6B and 6C. Data show the mean from 2 independent experiments. For statistical comparisons against the WT cell line for each tubulin isoform, single and double asterisks indicate $p<0.05$ and $p<0.01$, respectively; "ns" indicates not significant. See also Fig. 2D for data from HEK293 T-REx cells. (C) Total protein analysis by western blotting for FLAG-tagged SCAPER in the HeLa cell lines used in panels A, B, and Fig. 6B and 6C. (D–E) Quantification of steady state tubulin mRNA levels in the indicated HEK T-REx cell lines. Tubulin mRNA levels were normalized to a reference gene (RPLP1) and to the WT cell line, and data from all relevant experiments in the manuscript were compiled. Data show mean ± SD. Statistical analysis for KO and rescue cell lines was performed using a one-sample t-test. Values significantly different from 1 (WT levels) are indicated by single asterisks ($p<0.05$; "ns" indicates not significant). (D) n = 5, data reanalyzed from Fig. S1B and S1E. (E) n = 3, data reanalyzed from Fig. 5E.

Table S1. Cryo-EM data collection, processing, refinement and model statistics. Related to Fig. 3.

Data Collection	Dataset 1
Voltage (keV)	300
Pixel size (Å)	0.829
Detector	Gatan K3
Defocus range (µm)	-1.2 to -2.7
Electron dose (e^{-} $frame^{-1}$ $Å^{-2}$)	1
**Data Processing**	
Useable micrographs	19176 of 20932
Particles picked	1227269
Final particles	18949
Map sharpening B-factor ($Å^2$)	-20
Map resolution (Å)	2.8 (0.143 FSC)
EMDB accession code	EMD-16155
PDB accession code	8BPO
**Model Composition**	
Chains	62
Non-hydrogen atoms	143326
Protein residues	7633
RNA bases	3814
Metals (Mg^{2+}/Zn^{2+})	214/5
**Refinement**	
Model resolution (Å)	2.9 (0.5 FSC)<br>2.4 (0.143 FSC)
CC (mask)	0.85
**R.M.S deviations**	
Bond lengths (Å)	0.004
Bond angles (°)	0.851
**Validation**	
Molprobity score	1.40
Clashscore, all atoms	3.79
Rotamers outliers (%)	0.03
Cβ outliers (%)	0.03
**Ramachandran plot**	
Favored (%)	96.47
Allowed (%)	3.50
Outliers (%)	0.03

SUPPLEMENTAL REFERENCES

S1. Lin, Z., Gasic, I., Chandrasekaran, V., Peters, N., Shao, S., Mitchison, T.J., and Hegde, R.S. (2020). TTC5 mediates autoregulation of tubulin via mRNA degradation. Science *367*, 100–104. 10.1126/science.aaz4352.

S2. Ashkenazy, H., Abadi, S., Martz, E., Chay, O., Mayrose, I., Pupko, T., and Ben-Tal, N. (2016). ConSurf 2016: an improved methodology to estimate and visualize evolutionary conservation in macromolecules. Nucleic Acids Res *44*, W344–W350. 10.1093/nar/gkw408.

S3. Brinkman, E.K., Chen, T., Amendola, M., and van Steensel, B. (2014). Easy quantitative assessment of genome editing by sequence trace decomposition. Nucleic Acids Res *42*, e168–e168. 10.1093/nar/gku936.

S4. Yang, J., Anishchenko, I., Park, H., Peng, Z., Ovchinnikov, S., and Baker, D. (2020). Improved protein structure prediction using predicted interresidue orientations. Proc National Acad Sci *117*, 1496–1503. 10.1073/pnas.1914677117.

S5. Tatour, Y., Sanchez-Navarro, I., Chervinsky, E., Hakonarson, H., Gawi, H., Tahsin-Swafiri, S., Leibu, R., Lopez-Molina, M.I., Fernandez-Sanz, G., Ayuso, C., et al. (2017). Mutations in SCAPER cause autosomal recessive retinitis pigmentosa with intellectual disability. J Med Genet *54*, 698. 10.1136/jmedgenet-2017-104632.

S6. Fasham, J., Arno, G., Lin, S., Xu, M., Carss, K.J., Hull, S., Lane, A., Robson, A.G., Wenger, O., Self, J.E., et al. (2019). Delineating the expanding phenotype associated with SCAPER gene mutation. Am J Med Genet A *179*, 1665–1671. 10.1002/ajmg.a.61202.

S7. Wormser, O., Gradstein, L., Yogev, Y., Perez, Y., Kadir, R., Goliand, I., Sadka, Y., Riati, S.E., Flusser, H., Nachmias, D., et al. (2019). SCAPER localizes to primary cilia and its mutation affects cilia length, causing Bardet-Biedl syndrome. Eur J Hum Genet *27*, 928–940. 10.1038/s41431-019-0347-z.

S8. Wormser, O., Levy, Y., Bakhrat, A., Bonaccorsi, S., Graziadio, L., Gatti, M., AbuMadighem, A., McKenney, R.J., Okada, K., Riati, S.E., et al. (2021). Absence of SCAPER causes male infertility in humans and Drosophila by modulating microtubule dynamics during meiosis. J Med Genet *58*, 254–263. 10.1136/jmedgenet-2020-106946.

S9. Sievers, F., Wilm, A., Dineen, D., Gibson, T.J., Karplus, K., Li, W., Lopez, R., McWilliam, H., Remmert, M., Söding, J., et al. (2011). Fast, scalable generation of high-quality protein multiple sequence alignments using Clustal Omega. Mol Syst Biol *7*, 539–539. 10.1038/msb.2011.75.

S10. Waterhouse, A.M., Procter, J.B., Martin, D.M.A., Clamp, M., and Barton, G.J. (2009). Jalview Version 2—a multiple sequence alignment editor and analysis workbench. Bioinformatics *25*, 1189–1191. 10.1093/bioinformatics/btp033.
